# Acute activation of Gq signaling in pancreatic islet macrophages inhibits insulin secretion through AMPK/sphingolipid axis

**DOI:** 10.1172/JCI202344

**Published:** 2026-09-15

**Authors:** Simran Singh, Ashish Kumar, Sudipta Paul, Mriganka Sarkar, Santhosh Duraisamy, Ganesh Timalsina, Raashidha Farhath, Harender Yadav, Seema Kuldeep, Soumita Bhaumik, Kunj Kumar Prajapati, Saahiba Thaleshwari, Anuj Gargya, Tamojit Santra, Sonal Amit, Rashmi Parihar, Santosh K. Misra, Hamim Zafar, Luiz F. Barella, Michael A. Kalwat, Dharmaraja Allimuthu, Sai Prasad Pydi

**Affiliations:** 1 Molecular Metabolism & Cell Signaling Laboratory,; 2 Mehta Family Centre for Engineering in Medicine,; 3 Department of Chemistry, and; 4 CoSmic Lab, Department of Biological Sciences & Bioengineering, Indian Institute of Technology Kanpur, Kanpur, India.; 5 Autonomous State Medical College, Akbarpur, Kanpur, India.; 6 Central Experimental Animal Facility,; 7 Gangwal School of Medical Sciences and Technology, and; 8 Department of Computer Science & Engineering, Indian Institute of Technology Kanpur, Kanpur, India.; 9 Penguin Bio Corp., New York, New York, USA.; 10 Indiana Biosciences Research Institute, Indianapolis, Indiana, USA.; 11 Center for Diabetes and Metabolic Diseases, Indiana University School of Medicine, Indianapolis, Indiana, USA.

**Keywords:** Endocrinology, Metabolism, Beta cells, G protein-coupled receptors, Obesity

## Abstract

Obesity-associated inflammation impairs pancreatic β cell function, yet the mechanisms by which immune cells acutely regulate insulin secretion remain poorly defined. Here, we identify myeloid Gq signaling as an immunometabolic node linking macrophage lipid sensing to impaired insulin secretion. Using chemogenetic DREADD-mediated activation of myeloid Gq, we show that acute macrophage Gq activation impairs glucose-stimulated insulin secretion (GSIS) in vivo, whereas myeloid Gαq ablation enhances GSIS. Mechanistically, Gq activation rapidly induced AMPK phosphorylation and sphingolipid remodeling independently of canonical inflammatory cytokines. Macrophage-derived sphingolipids impaired β cell insulin signaling and GSIS through CD36/PKCζ, while inhibition of CD36, AMPK, or sphingolipid metabolism restored β cell function. We further identified GPR18, a Gq-coupled endocannabinoid-responsive GPCR, as an upstream regulator. GPR18 activation with *N*-arachidonoyl glycine (NAGly) recapitulated this phenotype, whereas myeloid *Gαq* deletion or *Gpr18/AMPK* silencing abolished it. GPR18 signaling predominantly engaged Gq rather than Gi pathways. In human tissues, *GPR18* was enriched in islet macrophages, and NAGly suppressed GSIS in primary human islets. Thus, a conserved macrophage GPR18/Gαq/AMPK/sphingolipid axis dynamically regulates β cell function and represents a potential therapeutic target in obesity and type 2 diabetes.

## Abstract 2

We show islet macrophages acutely suppress insulin secretion via a GPR18–Gq–AMPK–sphingolipid axis—revealing lipid-based immune–β-cell crosstalk as a target in obesity and diabetes.

## Graphical abstract

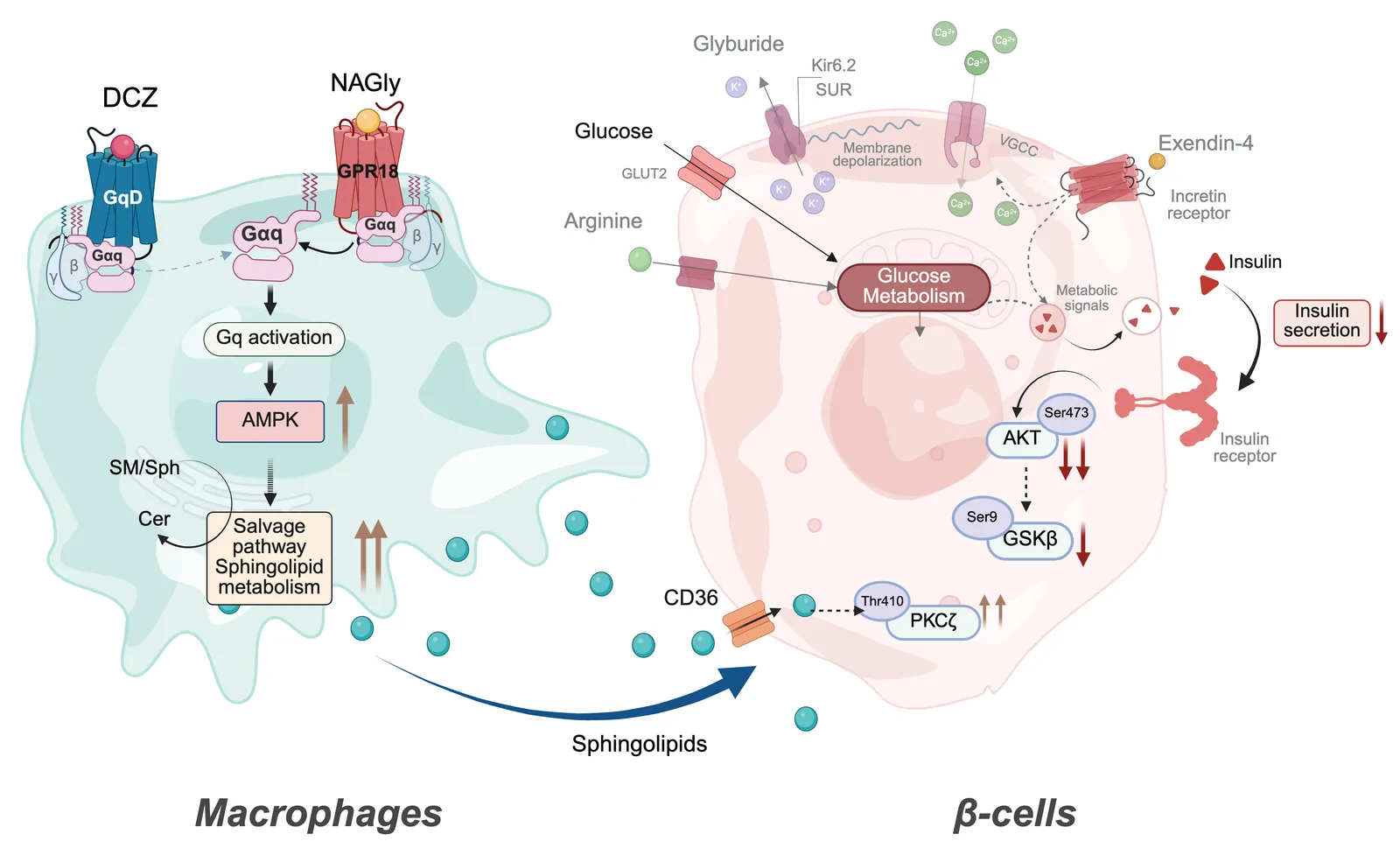

## Introduction

The pancreatic islet comprises a heterogeneous population of specialized cells functioning synergistically to carry out critical processes essential for insulin secretion and glucose homeostasis (1, 2). Within this microenvironment, β cells play a central role in glucose homeostasis by coupling glucose metabolism to insulin secretion through coordinated metabolic, mitochondrial, calcium, and exocytotic signaling (3, 4). These processes become progressively compromised during β cell failure associated with chronic metabolic disorders. Obesity is one of the major stressors compromising β cell function, where β cells initially compensate for obesity-associated insulin resistance by augmenting insulin output; however, prolonged metabolic overload leads to β cell exhaustion and failure. Importantly, obesity not only imposes direct metabolic stress on β cells but also profoundly alters the inflammatory and cellular composition of the pancreatic islet (5–7). Evidence from multiple investigations indicates an increased infiltration of myeloid lineage cells, mainly monocytes and macrophages, into the pancreatic islets in both obese animal models and humans (8–11).

Islet-resident macrophages exhibit significant functional plasticity: they impair β cell function via direct cell–cell interactions and engulfment of insulin granules; conversely, they promote β cell proliferation through PDGFR signaling (6). Emerging evidence suggests that blocking macrophage-derived exosomal miR-155 improves insulin sensitivity and glucose tolerance in obese and diabetic mice by enhancing β cell function through the miR-155/PDX1 axis (12). Following β cell injury, islet-resident macrophages adopt a reparative phenotype, characterized by increased production of IGF-1 and reduced expression of proinflammatory cytokines. This phenotypic shift supports insulin secretory capacity and contributes to metabolic homeostasis even under conditions of obesity and hyperglycemia (13). These studies underscore the potential role of macrophages in obesity-associated β cell dysfunction. Despite these advances, the intracellular signaling pathways that determine whether macrophages exert protective or detrimental effects on β cells remain poorly understood. Identifying the molecular mediators involved in this intercellular crosstalk will not only enhance our understanding of islet functioning but also unveil highly potent therapeutic targets.

Macrophages continuously integrate extracellular metabolic cues through membrane receptors, among which GPCRs represent an important class of nutrient-, lipid-, and metabolite-sensing receptors. Recent studies have shown that GPCRs are not only activated by classical ligands but also by various metabolic intermediates and secretory factors that play a central role in cellular metabolism. Despite their well-characterized role in other tissues, few studies have explored the role of GPCRs in pancreatic macrophages. Loss of GPR92 in islet macrophages can cause β cell dysfunction, disrupting glucose homeostasis (14), and activation of macrophage FFAR4 leads to IL-6 release and promotes β cell function (15). Moreover, studies in murine models suggest that Ccl27a is expressed by pancreatic β cells, where it interacts with chemokine receptors on islet macrophages; however, the signaling axis was disrupted upon prolonged high-fat diet (HFD) exposure (15, 16). Nevertheless, the function of macrophage GPCRs and the mechanisms governing their control of β cell activity remain to be elucidated. This is primarily because many GPCRs are expressed across diverse cell types and tissues, making it challenging to delineate macrophage-specific effects. Furthermore, the limited availability of highly selective agonists or antagonists for individual GPCRs has hindered mechanistic studies in vivo. Despite these challenges, comprehending the role of macrophage GPCRs in pancreatic islets is essential for advancing the development of innovative GPCR-targeted therapeutics for metabolic disorders. Specifically, understanding how acute and chronic activation of macrophage GPCR signaling affects β cell function under physiological and pathological conditions would help develop better drugs for treating obesity and type 2 diabetes.

Chemogenetics provides an effective means to circumvent the limitations associated with traditional receptor studies (17). This approach also facilitates the cell-specific expression of engineered receptors in a cell-specific manner, thereby allowing for precise temporal control of receptor activation. Previous studies have demonstrated that Gq signaling plays a diverse and crucial role in glucose and energy homeostasis within metabolically active tissues like pancreatic α and β cells, brown adipocytes, skeletal muscle, and white adipocytes (18–22). However, the role of myeloid cell Gq signaling, particularly under diet-induced obesity models, is not fully characterized. To address this gap, we expressed Gq DREADD in a cell-specific manner using the LysM-Cre model and characterized its metabolic role in healthy and obese states. Additionally, as proof of concept, we validated our findings using an endogenously expressed Gq-coupled receptor, GPR18, highlighting the relevance of this regulatory axis in both mice and humans. Insights from this study will provide guidance for developing better strategies to treat obesity and type 2 diabetes.

## Results

### Macrophage Gnaq expression is selectively upregulated during obesity in humans and mice.

To assess metabolic regulation of Gαq signaling in tissue-resident macrophages, we integrated public pancreatic islet scRNA-seq datasets from regular chow (RC), HFD, and db/db mice comprising 196,808 cells (23–26). The integrated murine pancreatic atlas identified 8 distinct cell populations, including a macrophage population defined by canonical markers (Figure 1A and Supplemental Figure 1, A and B; supplemental material available online with this article; https://doi.org/10.1172/JCI202344DS1). Consistent with previous observations (27), macrophages were the predominant immune cell in RC and HFD conditions, whereas db/db islets exhibited a distinct immune cell composition (Figure 1B). Our prior scRNA-seq study (16) demonstrated heterogeneity within the islet macrophage compartment, with a predominance of the proinflammatory (M1-like) phenotype. To determine whether Gq signaling is differentially regulated within this macrophage population under metabolic stress, we next examined *Gnaq* expression. Notably, *Gnaq* expression was increased in M1-like macrophages from HFD islets, suggesting diet-dependent regulation of Gq signaling (Figure 1C). Given the functional overlap between Gαq and Gα11 proteins, we next assessed *Gna11* expression in M1-like macrophages; interestingly, *Gna11* expression remained unchanged between RC and HFD conditions (Figure 1C).

To experimentally validate these findings, we assessed *Gnaq* expression in BMDMs from RC and HFD mice. Consistent with the scRNA-seq findings, *Gnaq* expression was significantly upregulated under HFD conditions and positively correlated with body weight (Figure 1D). In contrast, *Gna11* expression in BMDMs exhibited no significant difference or correlation with body weight across dietary conditions (Supplemental Figure 1, C and D). However, in peritoneal cells, we found that both *Gnaq* and *Gna11* were upregulated in HFD intervention (Supplemental Figure 1E). Body weight showed a positive, but nonsignificant, correlation, whereas blood glucose levels showed a significant positive correlation compared with the RC cohort (Supplemental Figure 1, F–I). To evaluate the translational relevance of these findings, we quantified *GNAQ* expression in human PBMC-derived macrophages and observed a positive correlation with BMI (Figure 1E). Additionally, a significant positive correlation was found between *GNAQ* mRNA levels and fasting glucose in human macrophages (Supplemental Figure 1J). However, *GNA11* expression showed no significant correlation with BMI or fasting glucose in the human samples (Supplemental Figure 1, K and L). Together, these findings suggest that macrophage *Gnaq/GNAQ* expression increases with obesity and may contribute to metabolic dysfunction.

### Acute activation of myeloid Gq signaling disrupts glucose homeostasis in lean and obese mice.

To evaluate the functional impact of myeloid Gq signaling on systemic metabolism, we generated mice expressing a Gq-coupled DREADD receptor under the control of LysM-Cre (LysM-GqD), targeting the myeloid lineage. From here on, we refer to Cre^+^ Gq-DREADD mice as LysM-GqD and Cre^–^ littermates carrying only the floxed allele as controls (Figure 1F). GqD expression in islets and BMDMs was confirmed by probing the HA tag using immunoblotting (Figure 1G and Supplemental Figure 1M). GqD expression was confirmed by qPCR in BMDMs, peritoneal cells, and islets, and by immunofluorescence in islet macrophages, confirming coexpression of the HA tag and F4/80^+^ (Supplemental Figure 1, N and O). GqD expression was also detected in liver, inguinal white adipose tissue, epididymal white adipose tissue, brown adipose tissue, and skeletal muscle of LysM-GqD mice but not in controls, consistent with LysM-Cre–mediated expression in tissue-resident myeloid cells (Supplemental Figure 1P). In the absence of deschloroclozapine (DCZ), body weight, glucose tolerance, and glucose-stimulated insulin secretion (GSIS) were comparable between genotypes, indicating no detectable constitutive DREADD activity (Supplemental Figure 2, A–C).

We first evaluated the systemic metabolic impact of acute myeloid Gq activation without assuming tissue specificity due to the widespread distribution of macrophages throughout metabolically active tissues. Following DCZ administration, LysM-GqD mice showed increased blood glucose and reduced plasma insulin at 30 min, with no change in plasma nonesterified free fatty acids (NEFAs) (Figure 1H). Based on these findings, subsequent metabolic assays were performed 45 min after DCZ administration (Figure 1I). Dose optimization identified 30 μg/kg DCZ as the most consistent dose (Supplemental Figure 2D), which was used thereafter. Under RC conditions, myeloid Gq activation impaired glucose and pyruvate tolerance but did not affect insulin tolerance, indicating impaired glucose clearance without altered insulin sensitivity (Figure 1, J and K, and Supplemental Figure 2E). The oral glucose tolerance test (OGTT) was also unchanged, suggesting preserved intestinal glucose handling (Figure 1L). Next, to determine whether myeloid Gq activation exerts similar effects under diet-induced obesity, control and LysM-GqD mice were placed on HFD for 12 weeks prior to metabolic experiments. Despite no difference in body weight between groups (Supplemental Figure 2F), Gq activation under HFD conditions resulted in a metabolic profile comparable with that of lean mice, including higher blood glucose levels, decreased insulin secretion, and no difference in plasma NEFAs (Figure 2A). Furthermore, glucose tolerance was compromised in LysM-GqD mice (Figure 2B), but insulin tolerance was unaffected (Figure 2C). To exclude potential confounding effects of differences in body composition, we performed Dual-energy X-ray absorptiometry (DEXA) body composition analyses and observed no significant differences in lean mass percentage or fat mass percentage between groups (Supplemental Figure 2G). We next performed glucose and insulin tolerance tests using dosing normalized to lean body mass. Consistent with our initial findings, lean mass–adjusted glucose tolerance testing confirmed significant impairments in glucose handling in LysM-GqD mice, whereas insulin sensitivity remained unchanged (Supplemental Figure 2, H and I). These findings demonstrate that the observed defects in glucose homeostasis are independent of body composition. To further investigate the underlying metabolic alterations, we performed pyruvate tolerance tests, which revealed impaired pyruvate handling in LysM-GqD mice (Supplemental Figure 2J). In contrast, OGTT showed no significant differences between groups (Supplemental Figure 2K).

To determine whether acute myeloid Gq activation affects adipose lipolysis, we performed β3-adrenergic receptor–stimulated lipolysis assays using CL 316,243 hydrate. Lipolytic responses were comparable between control and LysM-GqD mice, indicating that acute Gq activation does not promote adipose lipolysis (Figure 2D). Serum biochemical profiling 45 min after DCZ administration showed no differences in triglycerides, HDL, LDL, or liver injury markers (alanine aminotransferase, aspartate aminotransferase, and alkaline phosphatase), indicating no overt hepatic metabolic or functional alterations (Figure 2E and Supplemental Figure 2L). Furthermore, energy expenditure and respiratory exchange ratio were also unchanged under RC and HFD conditions, indicating that acute myeloid Gq activation selectively impairs glucose clearance without broadly affecting whole-body energy metabolism or substrate utilization (Supplemental Figure 3, A–X).

### Acute activation of myeloid Gq signaling suppresses β cell insulin secretion in both lean and obese mice.

To test whether the impaired glucose tolerance observed in myeloid Gq is due to changes in insulin secretion, we evaluated β cell response to different insulin secretagogues under RC and HFD conditions (Figure 2F). First, we administered glucose to the LysM-GqD and control groups maintained on the RC diet and measured plasma insulin levels at regular intervals. Interestingly, plasma insulin was significantly reduced in the LysM-GqD cohort, indicating a defect in GSIS (Figure 2G). Next, we tested glyburide and arginine to determine whether the suppression of insulin secretion is specific to glucose or to other insulinotropic stimuli. These secretagogues bypass glucose metabolism and stimulate insulin release through direct β cell depolarization. In response to glyburide, LysM-GqD mice showed a significant drop in insulin levels at 5 min after injection with an insignificant change in AUC (Figure 2H). Additionally, arginine causes reduced insulin secretion compared with the control group, indicating that activating Gq in myeloid cells hampers the β cell’s ability to secrete insulin (Figure 2I). Surprisingly, insulin secretion in response to exendin-4, a GLP-1 receptor agonist that enhances insulin secretion via cAMP-dependent pathways independently of glucose transport, was not altered between LysM-GqD and control group mice under RC conditions (Figure 2J).

In HFD-fed mice, activating myeloid Gq similarly reduced insulin secretion when responding to glucose, glyburide, and arginine, indicating a broad β cell secretory impairment (Figure 3, A–C). Remarkably, insulin secretion stimulated by exendin-4 was also decreased, suggesting that obesity increases a β cell’s sensitivity to myeloid Gq-related suppression, even with GLP-1 receptor activation (Figure 3D). Despite impaired exendin-4–stimulated insulin secretion, oral GSIS remained largely preserved in DCZ-treated GqD HFD-fed mice (Figure 3E). This is likely because oral glucose administration engages multiple compensatory mechanisms, including incretin hormone release like gastric inhibitory polypeptide (GIP) neural inputs, and gastric emptying, that collectively preserve insulin secretion independent of myeloid Gq signaling (28). These findings demonstrate that acute activation of myeloid Gq signaling reduces β cell insulin secretion across various stimuli, and HFD exposure further exacerbates this dysfunction.

As we observed impaired insulin secretion in vivo, to determine whether the myeloid cells in the islet’s microenvironment are sufficient to produce these effects, we isolated the islets from the LysM-GqD and control mice and treated them with various insulin secretagogues in the presence of DCZ (Figure 3F). In agreement with our hypothesis, DCZ-induced activation of Gq signaling in myeloid cells significantly decreased insulin secretion from isolated islets in response to high glucose, glyburide, arginine, and exendin-4 (Figure 3, G–J). These results establish that local macrophage-islet crosstalk is sufficient to impair β cell insulin secretion downstream of myeloid Gq activation.

To determine whether the impaired insulin secretion following myeloid Gq activation was dependent on intra-islet macrophages, we selectively depleted macrophages using clodronate liposomes. Clodronate- and PBS-loaded liposomes were prepared according to the protocol previously described (29) and administered intravenously to LysM-GqD and littermate control mice. Efficient macrophage depletion was confirmed by immunofluorescence and qPCR (Supplemental Figure 4, A–C). GSIS was significantly impaired in LysM-GqD islets, whereas macrophage depletion completely rescued insulin secretory function (Figure 3K), establishing intra-islet macrophages as the principal mediators of the β cell dysfunction elicited by acute myeloid Gq activation.

Because pancreatic islets are composed of multiple cell types, to further dissect the direct macrophage–β cell crosstalk, we employed a coculture system using BMDMs and INS-1 β cells at a 1:5 ratio, reflecting the relative abundance of macrophages to β cells in islets from HFD-fed mice (Figure 3L) (6). Conditioned media from DCZ-stimulated LysM-GqD BMDMs robustly suppressed insulin secretion from INS-1 cells in response to high glucose, exendin-4, glyburide, and arginine, mirroring the in vivo and ex vivo findings (Figure 3, M and N). In contrast, stimulation with FPL64176, a voltage-gated calcium channel activator that directly bypasses upstream signaling to trigger insulin granule exocytosis, elicited comparable insulin secretion between groups (Figure 3N). These results suggest that β cell calcium-dependent secretory machinery remains intact and that Gq-induced macrophage signals act upstream of calcium entry.

To assess the relative abundance of myeloid subpopulations within the islet microenvironment, we analyzed our previously published scRNA-seq dataset from C57BL/6 mice under RC and HFD conditions (16). Consistent with prior reports (30), neutrophils, monocytes, and dendritic cells were present at low abundance within pancreatic islets, whereas macrophages constituted the predominant immune cell population under both dietary conditions (Supplemental Figure 4, D and E), suggesting that macrophages are the principal myeloid population regulating islet function and β cell responses. To further exclude the contribution of neutrophils, we assessed macrophage and neutrophil marker expression following clodronate liposome treatment. Clodronate selectively reduced macrophage markers (*Cd68*, *F4/80*) without significantly altering the neutrophil markers *Ly6g* and *Retnlg* (Supplemental Figure 4F), consistent with the established selectivity of clodronate for macrophage depletion (31, 32). The observed trend toward increased Ly6g^+^ cells likely reflects compensatory neutrophil mobilization from bone marrow following rapid macrophage loss (33).

Next, we asked whether neutrophil Gq signaling can contribute to β cell dysfunction; we isolated primary bone marrow–derived neutrophils, stimulated them with DCZ, and transferred conditioned media to INS-1 β cells. In contrast to BMDM conditioned media, neutrophil conditioned media failed to alter insulin secretion following DCZ stimulation (Supplemental Figure 4G), indicating that the observed β cell dysfunction is driven by macrophages rather than neutrophils.

### Myeloid-specific deletion of Gαq leads to improved glucose clearance.

To determine whether loss of myeloid Gq signaling enhances insulin secretion, we generated LysM-Cre–mediated *Gnaq*-knockout mice (LysM-Gq–KO; Figure 4A) and confirmed reduced Gnaq expression in BMDMs (Supplemental Figure 5A). Body weight, fasting, fed blood glucose, and plasma insulin were comparable between genotypes (Figure 4, B–D). LysM-Gq KO mice showed improved glucose clearance during glucose tolerance tests (GTTs), while insulin tolerance tests (ITTs), pyruvate tolerance tests, and OGTTs were unchanged (Supplemental Figure 5, B–E). GSIS was increased at 5 min after glucose stimulation, whereas responses to glyburide, arginine, and exendin-4 were comparable between groups (Figure 4, E–H). Circulating triglycerides, NEFAs, lean mass, fat mass, and body fat percentage were unchanged (Supplemental Figure 5, F–H). Moreover, lean mass–normalized GTT and ITT showed no differences between genotypes (Supplemental Figure 5, I and J), indicating that myeloid Gαq deletion primarily enhances glucose-stimulated β cell secretion without altering insulin sensitivity or body composition.

### Myeloid Gq activation impairs β cell function via macrophage-derived paracrine lipid signals.

To determine whether acute myeloid Gq activation induces systemic inflammatory responses, we performed multiplex cytokine profiling in blood plasma from HFD-fed control and LysM-GqD mice 45 min after DCZ administration. Circulating levels of proinflammatory cytokines (IL-1β, TNF-α, IFN-γ, and IL-6) and antiinflammatory cytokines (IL-10 and IL-17A) did not differ significantly between groups (Figure 5A). Consistent with this, transcript levels of *IL-1β*, *IL-6*, *TNF-α*, *IFN-γ*, and *IL-10* in BMDMs isolated from control and LysM-GqD mice following 45 min of DCZ stimulation were similarly unaltered (Figure 5B). These findings indicate that acute Gq-mediated β cell dysfunction occurs independently of detectable classical cytokine signaling.

Given the established role of Gq signaling in lipid homeostasis, we next investigated how lipid dysregulation in macrophages contributes to β cell dysfunction. We performed untargeted cell lysate lipidomic analysis to determine whether macrophage-derived lipid mediators contribute to β cell dysfunction, stimulating BMDMs from control and LysM-GqD mice with DCZ for 0, 60, or 120 min (Figure 5C). Principal component analysis revealed no distinct separation between unstimulated control and LysM-GqD BMDMs. However, following DCZ treatment, control and LysM-GqD BMDMs formed 2 distinct clusters (Supplemental Figure 6A).

We next analyzed differentially regulated lipids at 0, 60, and 120 min and found that multiple sphingolipid species were significantly increased upon DCZ-mediated Gq activation at 60 and 120 min compared with untreated cells (Supplemental Figure 6B). Differentially abundant lipid species were predominantly sphingolipids. Acute Gq activation with DCZ resulted in significant remodeling of the sphingolipid landscape, characterized by a broader repertoire of sphingolipid subclasses at both 60 and 120 min compared with the 0 min time point (Figure 5D and Supplemental Figure 6, C and D). The overall sphingolipid fraction increased by approximately 1.46-fold at 60 min relative to baseline (Figure 5E). In contrast, comparable increases in lipid species number or overall lipid class abundance were not observed in other major lipid classes, including fatty acids, glycerolipids, and glycerophospholipids (Figure 5, E and F). Furthermore, enrichment analysis identified highly enriched metabolic pathways related to sphingolipid metabolism within 60 and 120 min of activation (Figure 5G and Supplemental Figure 6E). However, none of these pathways showed statistical significance.

We next asked whether these lipids were secreted and available to act as paracrine signaling molecules. To test this, we isolated lipids from the cell culture supernatants of DCZ-treated macrophages (Figure 6A). Partial least squares discriminant analysis (PLS-DA) revealed a clear separation between DCZ-treated control and LysM-GqD BMDMs (Supplemental Figure 6F). Several sphingolipid species, including different subtypes of ceramide (Cer) and sphingomyelin (SM) (Figure 6B and Supplemental Figure 6G), were significantly enriched in extracellular media from the LysM-GqD group, indicating active secretion accompanied by a highly enriched sphingolipid metabolic pathway (Supplemental Figure 6H).

### Macrophage-derived sphingolipid secretion is mediated by the AMPK signaling axis downstream of Gq activation.

As activation of Gq signaling in macrophages induces sphingolipid secretion and leads to β cell dysfunction, next we sought to delineate the molecular mechanism underlying this lipid release. Given the role of AMPK in lipid metabolism (34), we examined the activation status following acute Gq activation. As expected, upon Gq activation with DCZ, phosphorylation of AMPK at Thr^172^ increased within 5 to 15 min (Figure 6, C and D). To determine whether AMPK mediates the observed phenotype, we treated LysM-GqD BMDMs with the AMPK pharmacological inhibitor compound C (CpdC), followed by DCZ stimulation for 60 min. PLS-DA revealed a clear separation between control and LysM-GqD stimulated with CpdC and DCZ (Supplemental Figure 6I). Lipidomic analysis of cell-free supernatants revealed that AMPK inhibition altered the sphingolipid subspecies like Cer and SM (Figure 6E, Supplemental Figure 6J) compared with without inhibitor treatment (Figure 6B). Additionally, sphingolipid metabolism was not highly enriched (Supplemental Figure 6K).

To determine whether macrophage-derived lipid alterations were reflected within the islet microenvironment, we performed lipidomic profiling of whole islets isolated from control and LysM-GqD mice following DCZ administration. We observed increased levels of Cer, sphingosine (Sph), and SM species in the whole-islet lipidome of LysM-GqD mice (Figure 6F), consistent with the lipidomic alterations detected in LysM-GqD BMDMs following DCZ treatment (Figure 5F and Figure 6B). To further investigate whether these lipid mediators act through systemic circulation, we treated INS-1 β cells with blood plasma collected from DCZ stimulated control and LysM-GqD mice. In contrast to the effects observed with macrophage-conditioned media, blood plasma transfer did not significantly alter insulin secretion in INS-1 cells (Supplemental Figure 7A), suggesting that plasma circulating factors alone are insufficient to recapitulate the β cell dysfunction phenotype.

Together, these findings support a model where a myeloid Gq/AMPK axis drives local sphingolipid remodeling within the islet microenvironment, rather than eliciting a dominant systemic effect through circulating lipids.

### Macrophage-derived sphingolipids impair β cell insulin signaling via the CD36/PKCζ axis.

To determine how macrophage-derived sphingolipids impair β cell function and insulin signaling, we examined their functional effects on β cells. INS-1 cells were cultured in conditioned media from either control or LysM-GqD macrophages, and signaling was assessed following glucose stimulation. Cells exposed to LysM-GqD–derived supernatants showed a significant reduction in phosphorylation of AKT at Ser^473^ and GSK3β at Ser^9^ (Figure 7, A–C), indicating compromised intracellular insulin signaling in β cells. CD36 is known to transport lipid species such as Cers into cells (34). We next tested whether blocking CD36 could prevent signaling disruption. INS-1 cells were pretreated with the CD36 inhibitor sulfosuccinimidyl-oleate (SSO; 0.1 and 0.5 mmol/L) prior to exposure to conditioned media from LysM-GqD macrophages. Both concentrations restored insulin secretion, confirming that the observed effects are CD36 dependent (Figure 7D and Supplemental Figure 7B). To investigate downstream signaling events associated with CD36 activation, we assessed phosphorylation of PKCζ at Thr^410^. Exposure to conditioned media from GqD-activated BMDMs increased PKCζ phosphorylation while reducing AKT phosphorylation at Ser^473^. SSO treatment decreased pPKCζ (Thr^410^) and restored pAKT (Ser^473^) at both concentrations tested (Figure 7, E–G), further supporting involvement of the CD36/PKCζ axis in mediating the observed defects in insulin signaling and secretion. Consistent with previous reports, Cer accumulation activated PKCζ and suppressed AKT signaling (35). To rule out potential off-target effects of pharmacological CD36 inhibition, we performed siRNA-mediated knockdown of *CD36* in INS-1 cells. CD36 silencing restored insulin secretion in response to conditioned media from GqD-activated macrophages (Figure 7H), confirming the CD36-mediated mechanism.

To identify specific sphingolipids mediating macrophage-induced β cell dysfunction, we analyzed our lipidomic dataset and observed a coordinated increase in SM, Sphs, and multiple Cers following acute Gq activation (Figure 8, A–C). To determine whether sphingolipid remodeling contributed functionally, we pharmacologically targeted distinct metabolic steps (Figure 8D). Inhibition of Cer synthase with fumonisin B1 or neutral sphingomyelinase with GW4869 restored insulin secretion in β cells exposed to conditioned media from Gq-activated macrophages (Figure 8E). In contrast, inhibiting de novo sphingolipid synthesis with myriocin or *Sptlc1* siRNA (Supplemental Figure 7C) did not rescue the impaired insulin secretion (Figure 8, E and F). These findings indicate that macrophage Gq activation impairs β cell function primarily through SM turnover and downstream Cer remodeling rather than de novo sphingolipid synthesis. These findings align with human islet scRNA-seq analyses (36) where Sphingomyelin phosphodiesterase (SMPD)-associated signatures correlated positively with BMI, while CERS-associated pathways correlated positively with glucose and negatively with BMI (Supplemental Figure 7, D and E). These associations were not observed in granulocytes, suggesting relative specificity to islet macrophages (Supplemental Figure 7, F and G). Together, the data implicate Cer–SM remodeling in macrophage-mediated β cell dysfunction rather than de novo sphingolipid synthesis alone.

### GPR18 activation in macrophages suppresses insulin secretion via Gαq-dependent signaling.

To identify an endogenous Gαq-coupled GPCR that could recapitulate the LysM-GqD phenotype, we screened murine macrophage scRNA-seq datasets and validated the top candidates by qPCR. GPR18 was consistently expressed in BMDMs and enriched in M1 macrophages (Supplemental Figure 8A). qPCR further confirmed Gpr18 expression in macrophages and islets, but not in INS-1 cells (Figure 9A). scRNA-seq analysis revealed that Gpr18 was among the most highly expressed genes in immune cells compared with non-immune cells (Supplemental Figure 8B). Gpr18 expression increased in peritoneal cells from HFD-fed mice and correlated with body weight and blood glucose (Figure 9, B–D). Finally, *N*-arachidonoyl glycine (NAGly) induced dose-dependent IP1 accumulation in BMDMs, confirming GPR18-mediated Gq signaling (Supplemental Figure 8C).

To assess the functional relevance of this pathway in vivo, HFD-fed C57BL/6NJ mice received NAGly (36.15 μg/kg) followed by glucose, arginine, or glyburide challenge (Figure 9E). NAGly reduced insulin secretion in response to all 3 stimuli, with reduced AUCs for glucose and glyburide (Figure 9, F–H). NAGly failed to suppress insulin secretion in LysM-Gq KO mice, demonstrating a requirement for myeloid Gq signaling (Figure 9, I–L). Given previous reports that Gpr18 may also signal through Gαi, we next sought to rule out the involvement of inhibitory G proteins. Disruption of myeloid Gi signaling in LysM-PTX mice did not alter the NAGly response, supporting Gq- rather than Gi-dependent signaling (Figure 9, M and N). Body weight was comparable across genotypes (Supplemental Figure 8, D–F). GPR18 activation inhibited GSIS in islets from WT but not LysM-Gq KO mice, demonstrating a role of myeloid Gq signaling (Figure 10A). Similarly, clodronate-mediated macrophage depletion abolished NAGly-induced inhibition of GSIS in HFD islets, implicating role of resident macrophages (Figure 10B). In coculture experiments, NAGly-treated conditioned media from WT, but not LysM-Gq KO, BMDMs suppressed insulin secretion from INS-1 β cells (Figure 10C). However, glyburide-, arginine-, and exendin-4–stimulated insulin secretion was unaffected (Supplemental Figure 8, G–I).

### Macrophage GPR18 activates the Gq/AMPK pathway to promote sphingolipid release.

To dissect the mechanism by which GPR18 mediates its effects, we first tested whether its activation recapitulates the lipid signaling observed with chemogenetic acute Gq activation. BMDMs from WT mice were treated with the GPR18 agonist NAGly or vehicle, and cell lysate was collected after 60 min for lipidomic analysis (Figure 10D). PLS-DA revealed a clear separation between vehicle- and NAGly-treated WT BMDMs as well as between NAGly-treated WT and LysM-Gq KO BMDMs (Supplemental Figure 9, A and B). Consistent with the lipidomic profile of LysM-GqD mice (Figure 5F and Figure 6, B and E), NAGly treatment promoted the accumulation of Cer, SM, and Sph species in WT BMDMs (Figure 10D). This response was abolished in NAGly-treated LysM-Gq KO BMDMs (Supplemental Figure 9C), indicating that NAGly-induced sphingolipid remodeling requires myeloid Gq signaling. Pathway analysis further confirmed that sphingolipid metabolism was one of the highly enriched pathways in NAGly-treated WT cells (Figure 10E).

To delineate the downstream signaling cascade, we examined AMPK phosphorylation (Thr^172^) by immunoblotting. In WT macrophages, NAGly induced a rapid increase in AMPK phosphorylation within 5–15 min, an effect completely abolished in LysM-Gq KO cells (Figure 10, F and G). To further validate the role of GPR18 in this signaling axis, we assessed AMPK phosphorylation in scramble control and Gpr18-silenced BMDMs following NAGly stimulation (Supplemental Figure 9D). NAGly increased pAMPK (Thr^172^) in scramble control BMDMs; however, this response was abolished upon Gpr18 knockdown, confirming that GPR18 regulates AMPK activation (Figure 10, H and I). NAGly-stimulated conditioned media from control BMDMs impaired INS-1 insulin secretion, whereas Gpr18-silenced BMDM media did not, demonstrating that this effect depends on macrophage GPR18 signaling (Figure 10J).

We next examined β cell insulin signaling by assessing AKT phosphorylation at Ser^473^ following treatment with conditioned media from scramble control or Gpr18-knockdown BMDMs with or without NAGly stimulation. Conditioned media from NAGly-treated scramble control BMDMs markedly reduced pAKT (Ser^473^), a key mediator of β cell autocrine insulin signaling, whereas this reduction was abolished using conditioned media from Gpr18-deficient BMDMs, further supporting a GPR18-dependent mechanism (Figure 10, K and L). To determine whether AMPK mediates the effects of GPR18 activation on macrophage–β cell communication, BMDMs were transfected with scramble control or *AMPKα1/2*-targeting siRNA prior to NAGly stimulation, and conditioned media was transferred to INS-1 cells for GSIS assessment by insulin ELISA (Supplemental Figure 9E). Conditioned media from NAGly-treated scramble control BMDMs significantly impaired insulin secretion, whereas conditioned media from AMPK-silenced BMDMs restored insulin secretion, indicating that AMPK activation downstream of GPR18 is required for release of the macrophage-derived factors that suppress β cell function (Figure 10M).

Collectively, these findings establish GPR18 as an endogenous upstream activator of the macrophage Gαq/AMPK/sphingolipid signaling axis identified through chemogenetic Gq activation.

### The GPR18/Gαq/AMPK axis is conserved in human macrophages and impairs human islet insulin secretion.

To determine whether this signaling axis is conserved in human immune cells, we searched human islet scRNA-seq data for *GPR18* expression, and *GPR18* was highly expressed in immune cells, including macrophages (Supplemental Figure 9F). Next, to confirm these observations, we differentiated human PBMCs into macrophages and assessed *GPR18* expression. *GPR18* levels positively correlated with BMI (Figure 11A), and *GPR18* expression significantly increased in proinflammatory M1 macrophages compared with M0 controls (Figure 11B). To further evaluate the translational relevance of our findings, we performed static GSIS assays using isolated human islets (Figure 11C). Activation of GPR18 signaling with NAGly significantly suppressed insulin secretion compared with untreated control islets, indicating that NAGly/GPR18 signaling negatively regulates β cell function in human islets (Figure 11D). To further establish the clinical relevance of this signaling cascade, we examined the relationship between *GPR18* and *GNAQ* gene expression in human macrophages and observed a strong positive correlation, supporting coordinated expression of the receptor and its downstream G protein signaling partner in human immune cells (Supplemental Figure 9G). Finally, to validate the involvement of the GPR18/Gαq/AMPK signaling axis in human immune cells, human PBMC-derived macrophages were treated with the Gαq inhibitor UBO-QIC (FR900359) and the AMPK inhibitor CpdC prior to NAGly stimulation (Figure 11E). NAGly induced phosphorylation of AMPK at Thr^172^, whereas treatment with either UBO-QIC (FR900359) (1 μM) or CpdC (10 μM) attenuated this response, supporting a model in which GPR18 activates AMPK through Gαq signaling in human macrophages (Figure 11, F and G). To further investigate the lipidomic profile of human PBMC-derived macrophages, we activated GPR18 using NAGly and inhibited downstream signaling through Gαq (1) and AMPK1/2 (CpdC) (Figure 11E). PLS-DA revealed distinct clustering of untreated and NAGly-treated PBMC-derived macrophages as well as of NAGly-treated cells in the presence or absence of UBO or CpdC (Supplemental Figure 9, H–J). Specifically, NAGly treatment led to a significant increase in sphingolipid subspecies; however, this effect was abolished when GPR18 activation occurred concurrently with inhibition of Gαq or AMPK (Figure 11H). Consistent with these findings, pathway enrichment analysis revealed that sphingolipid metabolic pathways were highly enriched upon GPR18/NAGly axis activation, while inhibition of these intermediate signaling nodes prevented this metabolic shift (Figure 11H). To further establish the relevance of this pathway in humans, we analyzed the correlation between *GPR18* expression and the expression of ceramide synthase genes (*CERS1–6*) in human macrophages. We observed positive correlations between *GPR18* and each of the *CERS* family genes, supporting a close association between GPR18 signaling and the Cer biosynthetic program in human immune cells (Supplemental Figure 9K). Collectively, these findings demonstrate that the GPR18/Gαq/AMPK pathway is conserved in human immune cells and negatively regulates insulin secretion in human islets, highlighting the translational relevance of our mechanistic observations.

## Discussion

Most tissue-resident macrophages originate during embryogenesis and act as highly specialized immunometabolic sentinels, integrating nutritional, lipid, and metabolic signals to regulate both immune tone and local metabolic programs (6, 31, 37). Beyond producing cytokines, macrophages generate bioactive metabolites that influence stress adaptation and cellular homeostasis. Although several lipid mediators have been implicated in chronic β cell dysfunction (38–40), the upstream immune signals that drive their release, particularly under acute conditions, remain poorly understood.

We identified macrophage Gq signaling as a regulator of β cell function and glucose homeostasis. Acute Gq activation suppresses β cell insulin secretion through macrophage-derived sphingolipids, including Cers, whereas myeloid Gαq deletion enhances GSIS and glucose tolerance. We further identified GPR18 as an upstream Gq-coupled receptor whose agonist NAGly suppresses insulin secretion in vivo in a macrophage Gq-dependent manner, defining a GPR18/Gq/sphingolipid axis in immune–islet crosstalk. Our chemogenetic LysM-GqD model showed that acute myeloid Gq activation impaired glucose tolerance and GSIS without affecting insulin sensitivity, suggesting a preferential effect on insulin secretion (Figures 1–3). β Cell suppression occurred across glucose, glyburide, and arginine stimulation, while exendin-4 responses were preserved under RC but blunted under HFD conditions, indicating obesity-dependent sensitization (Figure 3). Mechanistically, inhibition occurred upstream of Ca^2+^ entry, as FPL64176 bypassed the defect, indicating intact β cell Ca^2+^ influx and exocytotic machinery and implicating a macrophage-derived factor in suppressing depolarization-driven insulin secretion (41). Our LysM-GqD model demonstrated that activating a single signaling node in macrophages is sufficient to phenocopy these effects acutely. Conversely, LysM-Gq–KO mice had enhanced glucose clearance and a trend toward improved GSIS without changes in weight or insulin sensitivity (Figure 4). Thus, both gain and loss of function of macrophage Gq produce reciprocal effects on insulin secretion and glucose tolerance, underscoring a causative role.

Indirect coculture of murine macrophages with rat insulinoma cells reproduced the inhibitory effect, implicating soluble mediators. This rapid suppression occurred without changes in circulating or macrophage cytokines, indicating a mechanism distinct from canonical inflammatory signaling. Lipidomics revealed increased sphingolipid secretion within 1 hour of Gq activation, which was AMPK dependent, as CpdC blocked sphingolipid production and preserved β cell insulin secretion. Functionally, these sphingolipids traversed β cell membranes and inhibited AKT phosphorylation at Ser^473^, a known molecular mechanism for Cer-induced insulin resistance (42, 43). Notably, while previous work implicated adipose, liver, and muscle Cer pools in systemic insulin resistance (44, 45), our data positioned islet macrophages as a rapid, local source of sphingolipids capable of suppressing β cell function independent of chronic inflammation. Pharmacological inhibition of Cer synthase or neutral sphingomyelinase restored insulin secretion, whereas inhibition of de novo sphingolipid synthesis with myriocin or Sptlc1 silencing was ineffective. These findings indicate that acute macrophage Gq activation primarily promotes SM turnover and downstream Cer remodeling rather than de novo sphingolipid synthesis. Such rapid lipid remodeling may impair β cell function before transcriptional or metabolic adaptations associated with chronic lipid overload. Cers can further impair insulin signaling through PP2A-mediated AKT dephosphorylation and PKCζ-dependent inhibition of AKT membrane translocation (42). Our findings identified CD36 as a mediator of macrophage-derived sphingolipid uptake in β cells. CD36 inhibition or knockdown restored insulin secretion and normalized PKCζ and AKT phosphorylation, supporting a model in which lipid uptake drives β cell dysfunction via the Cer/PKCζ/AKT pathway (42). While Cer accumulation is typically considered a chronic process during overnutrition, our findings showed that acute Gq signaling activation releases Cers and influences the β cell function. Previous studies showed that Cers can signal through FPR2 in adipocytes (46), raising a possibility of β cell inhibition; however, scRNA-seq detected no FPR2 expression in β cells, suggesting that alternative pathways mediate Cer effects in the islet microenvironment.

We identified GPR18 as a physiological receptor mediating this pathway. GPR18 activation with NAGly reproduced Gq-DREADD effects, which were absent in LysM-Gq–KO mice, confirming a requirement for myeloid Gq signaling. These findings support rapid macrophage secretome remodeling through Gq-dependent Ca^2+^ and lipid signaling (47). Our findings extend prior work on islet macrophage GPCRs, where FFAR4, another Gq-coupled receptor, promotes GSIS via IL-6 secretion under physiological conditions (15), while GPR92 deficiency impairs insulin secretion and enhances islet inflammation through cytokine-mediated mechanisms (14). In contrast, Gq/GPR18 signaling suppresses GSIS via lipid mediators, revealing an immune cell–islet communication axis. Our identification of GPR18 as a Gq-coupled receptor that suppresses β cell insulin secretion extends its known roles in islet functions. In cardiovascular and liver systems, activation of myeloid GPR18 by the specialized proresolving mediator Resolvin D2 promotes efferocytosis, increases reparative Arg1^+^ macrophages, and limits plaque necrosis and fibrosis (48, 49). These effects arise alongside an antiinflammatory lipid profile enriched in specialized proresolving mediators. In contrast, our work showed that acute Gq-biased activation of GPR18 in islet macrophages triggers rapid AMPK-dependent release of sphingolipids, including Cers. Whereas specialized proresolving mediators promote repair and metabolic balance, Cers impair insulin signaling and blunt insulin secretion. This highlights a ligand- and context-dependent switch in GPR18 outputs. Our study identified a macrophage–to–β cell communication axis mediated by Gq signaling and lipid secretion, expanding the concept of metaflammation in diabetes. Beyond cytokines and cell–cell contact, our findings implicate Cers as paracrine mediators linking macrophage Gq activation to β cell dysfunction. Increased fatty acids and endocannabinoid metabolites such as NAGly in obesity and type 2 diabetes may chronically engage this pathway, whereas targeting the GPR18/Gq axis could preserve β cell function.

Human data support the translational relevance of the GPR18/Gαq/AMPK/sphingolipid axis. GPR18 expression in human islet macrophages correlated with BMI and increased with proinflammatory polarization. GPR18 activation suppressed insulin secretion in human islets and induced Gαq-dependent AMPK activation and sphingolipid remodeling in human macrophages, suggesting a conserved immune/endocrine pathway contributing to β cell dysfunction in obesity and type 2 diabetes. A previous study reported that arachidonic acid–containing lipids are enriched in obesity, likely reflecting alterations in lipid metabolic pathways (50). By establishing macrophage Gq signaling as a central regulator of β cell function, our study provides a conceptual framework for understanding immune cell–islet crosstalk in obesity and diabetes.

There are limitations to our study. Although our data support a causal role for macrophage-derived sphingolipids in β cell dysfunction, the contribution of individual species remains unclear. The acute chemogenetic model may also not fully capture chronic macrophage activation during obesity, warranting long-term manipulation of the GPR18/Gαq pathway in islet macrophages. We also acknowledge that LysM-Cre targets multiple myeloid populations. Although mechanistic studies used purified BMDMs and clodronate-mediated macrophage depletion rescued the phenotype in vivo, contributions from other LysM-expressing cells cannot be excluded.

## Methods

Additional details are provided in the supplemental material.

### Sex as a biological variable

For the human studies, samples from both male and female individuals were studied. For the animal and cell culture studies, samples from both male and female animals were included. However, metabolic tests were carried out separately in male and female mice to eliminate baseline sex differences.

### Drugs, reagents, commercial kits, and antibodies

The sources of all drugs, reagents, commercial kits, antibodies, and mouse strains are detailed in the resource table in the supplemental material.

### BMDM culture

BMDMs were derived from male and female mice aged between 8 and 12 weeks and were maintained on either RC or HFD, per established protocols (4). Bone marrow was flushed from femurs and tibias with ice-cold DMEM, filtered through a 100 μm strainer, and centrifuged at 500*g* for 5 min at 4°C. Cells were cultured in complete medium with 20 ng/mL M-CSF for 6 days, with fresh M-CSF medium added on day 4, and used for downstream assays from day 6 onward.

### Static GSIS

After islet isolation, islets were incubated in islet culture medium (RPMI-1640 supplemented with 10% FBS, 1% penicillin-streptomycin, and 5.5 mM glucose) at 37°C in a humidified 5% CO_2_ atmosphere for 6 hours. After 6 hours of incubation, the islets were incubated in KRBH buffer (128.8 mM NaCl, 4.8 mM KCl, 1.2 mM KH_2_PO_4_, 1.2 mM MgSO_4_, 2.5 mM CaCl_2_, 5 mM NaHCO_3_, 10 mM HEPES, and 0.1% BSA) without glucose for 30 min and stimulated with DCZ (30 nM)/NAGly (10 μM) for 45 min in 2.8 mM glucose. After 45 min, supernatant was collected and stimulated in 16.8 mM glucose for 30 min (51). Supernatant was collected and ELISA was performed using the ALPCO STELLUX chemiluminescence kit. Data were normalized with the total DNA content of the islets.

### BMDM and INS-1 cell culture and coculture

Prosenjit Mondal (IISER Berhampur, Odisha, India) provided the INS-1 cells. INS-1 cells (6 × 10^4^/well) and BMDMs (1 × 10^4^/well) were seeded in 96-well plates. On day 4, BMDMs were starved for 60 min in KRBH buffer and INS-1 cells for 15 min in basal RPMI containing 1 mM glucose. BMDMs were treated with DCZ (30 nM) for 45 min, after which conditioned medium was transferred to INS-1 cells. Cells were then stimulated with glucose or test secretagogues, and insulin secretion was measured by ELISA (STELLUX chemiluminescence kit). For CD36 inhibition, INS-1 cells were pretreated with SSO (0.5 mM) for 30 min at 37°C, washed twice, and subjected to GSIS.

### Clodronate liposomes and delivery

Clodronate liposomes were prepared as previously described (29). Clodronate liposomes were prepared by dissolving phosphatidylcholine (86 mg) and cholesterol (8 mg) in chloroform, evaporating the solvent, and hydrating the lipid film with 10 mL of 0.6 M clodronate. The suspension was sonicated, incubated under nitrogen, and centrifuged to remove unencapsulated drug. Liposomes were washed 2–3 times with sterile PBS and resuspended in 4 mL PBS. Mice received clodronate liposomes intravenously at 10 μL/g body weight for macrophage depletion.

### siRNA-mediated knockdown of Gpr18, AMPK, and CD36 in BMDMs

BMDMs were seeded at 2 × 10^5^ cells/well in 6-well plates and transfected at 60%–80% confluency with siRNAs targeting *Gpr18*, *Ampk*, or *Cd36* or with nontargeting siRNA using lipofectamine RNAiMAX (Thermo Fisher Scientific) according to the manufacturer’s instructions. siRNA–lipid complexes were prepared in Opti-MEM (Gibco, 31985062) and added to cells, followed by incubation at 37°C and 5% CO_2_. Medium was replaced after 24 hours, and cells were cultured for an additional 24–48 hours before experiments. Knockdown efficiency was confirmed by RT-qPCR, with scrambled siRNA serving as the negative control.

### Untargeted lipidomics

#### Cell lysates and islets.

BMDMs (0.5 × 10^6^ cells/well) and 100 islets per time point were treated with DCZ (30 nM) for 60 or 120 min. Cells were washed twice and scraped into PBS. Lipids were extracted with chloroform/methanol (2:1, v/v), vortexed, and centrifuged at 2,800*g* for 5 min. The lower organic phase was collected, supplemented with 50 μL formic acid, dried under N_2_, and stored for analysis.

#### Cell supernatants.

Supernatant (500 μL) was mixed with methanol (1.5 mL) and MTBE (5 mL), incubated for 1 hour at 40°C with shaking, and phase-separated by addition of 1.25 mL MS-grade water. After centrifugation (1,000*g*, 10 min), the upper organic phase was collected and the aqueous phase reextracted with MTBE/methanol/water (10:3:2.5, v/v/v). Combined organic extracts were dried under N_2_.

#### LC-MS/MS.

Untargeted lipidomics was performed using an Agilent 1290 Infinity II UHPLC coupled to an Agilent 6546 QTOF mass spectrometer. Extracts were reconstituted in methanol/isopropanol (1:1, v/v) and maintained at 4°C, and 5 μL was injected onto an Agilent ZORBAX Eclipse Plus C18 column (2.1 × 50 mm, 1.8 μm) at 60°C.

### Data analysis

#### Data conversion and preprocessing.

Raw files (.d) were converted to mzXML using MSConvert (ProteoWizard v3.0.25163-1e83dfc) (52, 53) with centroid peak picking. Data were processed in MZmine v4.7.8 for mass detection, chromatogram building, Savitzky-Golay smoothing, isotope filtering, feature alignment, and gap filling using defined mass-to-charge ratio and retention time tolerances.

#### Feature annotation.

Unified peak lists (CSV, MZmine 2 format) were generated for each dataset. LipidFinder (54) was used in positive ion mode against the COMP_DB database with all positive ion adducts. Pathway enrichment analysis was performed using functional analysis (LC-MS) in MetaboAnalyst 6.0 (54).

#### Quality control and normalization.

Sample normalization (normalization by sum), data transformation (log_10_), and scaling (Pareto scaling), along with missing value imputation (1/5 of the minimum positive value), were performed using MetaboAnalyst 6.0.

#### Statistical analysis.

Replicates were averaged per biological sample. Differential features, principal component analysis (PCA), and PLS-DA were analyzed using MetaboAnalyst 6.0. Volcano plots used thresholds of |log_2_FC| ≥ 0.25 and *P* < 0.05. Weighted correlation network analysis was used for lipid–lipid network analysis. Heatmaps and plots were generated using pheatmap, ggplot2, EnhancedVolcano, and webR in R v4.4.1.

### Integration of publicly available scRNA-seq datasets for islet cells

To characterize Gpr18 expression in immune cells, we analyzed publicly available scRNA-seq datasets GSE329967, GSE203376, GSE211799, and GSE162512 (Gene Expression Omnibus [GEO]), encompassing RC, HFD, and db/db conditions. The integrated atlas contained 196,808 cells and 34,220 features. Data were preprocessed and cell types annotated using established marker genes. Datasets were integrated using scDREAMER (56), with condition treated as the batch variable, followed by clustering and UMAP visualization using Seurat (57). Gpr18 expression across immune cell populations was assessed by differential expression and violin plots. Expression of GPR18 in human pancreatic immune cells was validated using healthy samples from GSE229413 (GEO).

### Human islet static GSIS

Cadaveric human islets were obtained through the Integrated Islet Distribution Program and cultured in complete CMRL-1066 medium upon receipt. Hand-picked islets (100/tube) were washed twice with KRBH and preincubated for 30 min in glucose-free KRBH with or without NAGly (10 μM). Islets were then incubated for 60 min in 2.8 mM glucose ± NAGly, followed by stimulation with 16.8 mM glucose ± NAGly for an additional 60 min. Supernatants were collected, centrifuged, and stored at −80°C. Total insulin was extracted from islets using RIPA buffer. Secreted and total insulin was quantified using the U-Plex mouse insulin assay (Meso Scale Discovery).

### Statistics

Data are presented as the mean ± SEM. Statistical analyses were performed using 2-tailed Student’s *t* test or 2-way ANOVA with Bonferroni’s correction, as appropriate, in GraphPad Prism. *P* < 0.05 was considered significant.

### Study approval

The research methods described in this manuscript adhere to all pertinent ethical regulations. All animal experiments were approved by the Institutional Animal Ethics Committee at the Indian Institute of Technology Kanpur.

### Data availability

All data values supporting the findings of this manuscript are provided in the Supporting Data Values file. All materials generated in this study are available upon request from the corresponding author. Uncropped blots and gels are available in the supplemental material. This paper does not report any original code.

## Manuscript information

### Author contributions.

SS, AK, LFB, DA, and SPP designed the experiment. SS conducted Gq-DREADD and GPR18 in vivo studies. AK conducted Gq-KO in vivo studies. GT, SS and SD performed lipidomics, with analysis by MS, AG, DA, and SPP. AK performed islet isolation, with assays performed by SS. AK, TS, and SKM performed liposome studies. AK and ST performed immunostaining. HY performed cytokine multiplexing. SS, AK, HY, SK, SB, RP, and SA performed human sample collection and analyses. MS, SS, and HZ performed scRNA-seq analysis. SP, KKP, and RF assisted with mouse plasma and immunoblot studies. MAK performed human islet studies. SPP conceptualized the project. SS, AK, and SPP wrote the manuscript with input and approval from all authors. SS and AK contributed equally to the study; SS is listed first based on the overall contribution to the final manuscript. This order does not diminish the equal contribution of either author.

### AI disclosure.

ChatGPT (GPT-5.6, OpenAI) and Grammarly (Grammarly Inc.) were used for language refinement during manuscript preparation from September 2025 through May 2026.

### Conflict of interest.

The authors have declared that no conflict of interest exists.

### Funding.

This work was supported by the following:

- Department of Science and Technology–Science and Engineering Research Board grant CRG/2021/004502 (to SPP).
- Department of Biotechnology (DBT)/Wellcome Trust India Alliance grant IA/I/21/1/505613 (to SPP).
- DBT grant B1/PR44526/MED/30/2376/2021 (to SPP).
- Indian Council of Medical Research (ICMR) grant 5/4/8-18/Obs/SPP/2022-NCD-II (to SPP).
- Initiation grant from the Indian Institute of Technology Kanpur (to SPP).
- DBT Junior Research Fellowship (DBTHRDPMU/JRF/BET/-20/1/2020/AL/352; to SS).
- Ministry of Human Resource Development fellowship from the Indian Institute of Technology Kanpur (IITK/21118264; to AK).
- Breakthrough T1D award (3-SRA-2025-1630-S-B; to MKK).
- Indiana Biosciences Research Institute (to MAK).
- Human pancreatic islets were provided by Prodo Laboratories and the National Institute of Diabetes and Digestive and Kidney Diseases–funded Integrated Islet Distribution Program at City of Hope (RRID), supported by NIH grant 2UC4DK098085.
- The confocal facility at the Institute Animal House was established through the ICMR Centre of Excellence grant 5/3/8/20/2019-ITR.
- The central microscopy facility and the central histology facility–Department of Biological Sciences and Bioengineering, Indian Insitute of Technology were established through Department of Pharmaceuticals grant 31026/64/2024-MD.

This work is the result of NIH funding, in whole or in part, and is subject to the NIH Public Access Policy. Through acceptance of this federal funding, the NIH has been given a right to make the work publicly available in PubMed Central.

## Supplementary Material

Supplemental data

Unedited blot and gel images

Supporting data values

## Figures and Tables

**Figure 1:**
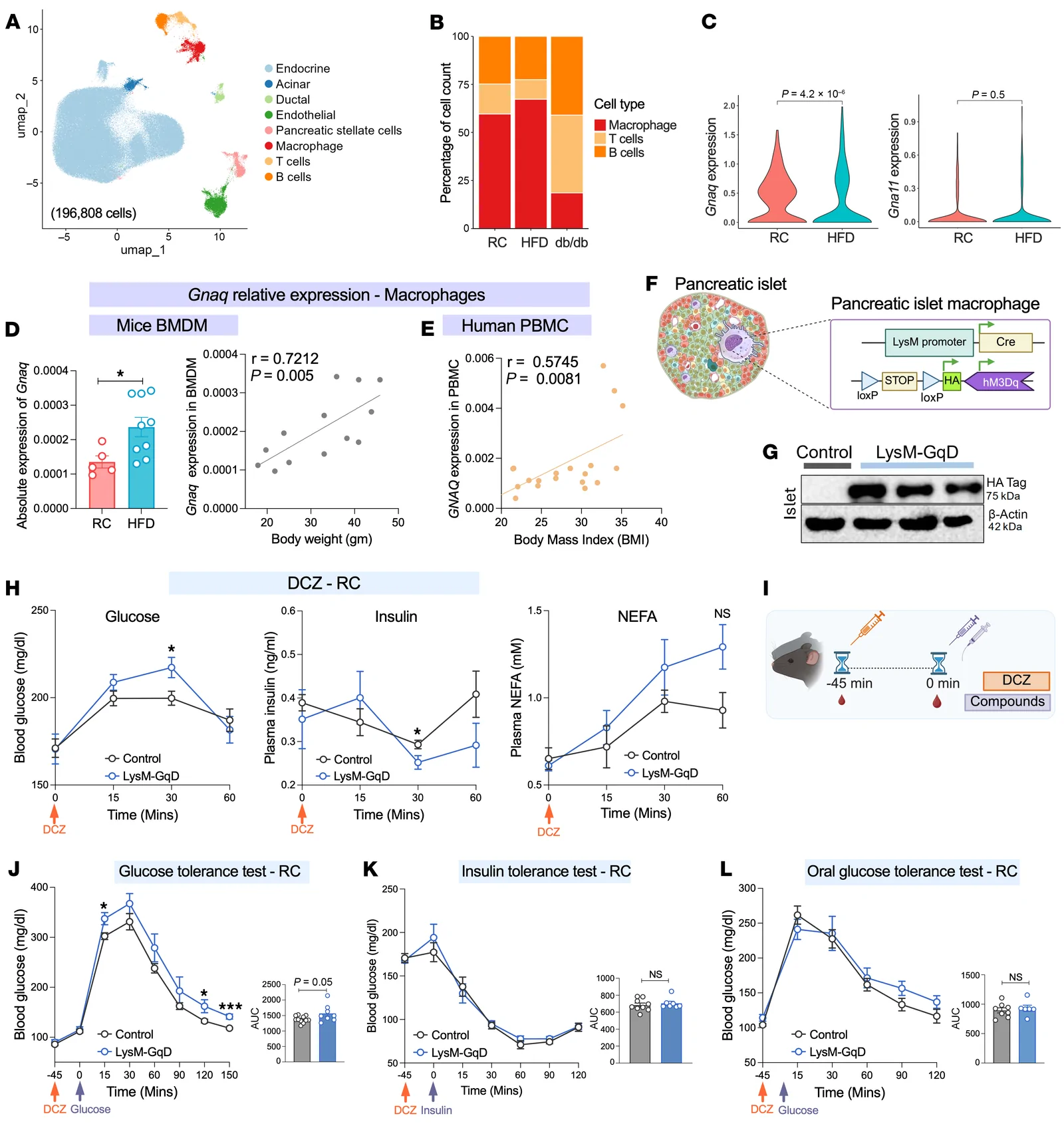
Acute myeloid Gq activation perturbs glucose homeostasis in lean and obese mice. (**A**) UMAP of murine pancreatic islet cell types from RC- and HFD-fed mice (8–24 weeks) and db/db mice (196,808 cells). (**B**) Distribution of islet immune cell types across conditions. (**C**) *Gnaq* and *Gna11* expression in scRNA-seq of islet macrophages from RC- and HFD-fed mice. (**D**) *Gnaq* expression in BMDMs and its correlation with body weight in RC-fed (*n* = 5) and HFD-fed (8–16 weeks; *n* = 13) mice. (**E**) Correlation of human PBMC *GNAQ* expression with BMI (*n* = 20). (**F**) Schematic of the LysM-GqD model. (**G**) GqD expression in islets by probing HA tag by immunoblotting control and LysM-GqD islets (*n* = 2–3). (**H**) Blood glucose, plasma insulin, and NEFAs following DCZ administration (30 μg/kg, i.p.; *n* = 6–9). (**I**) Metabolic testing workflow. (**J**–**L**) GTT (2 g/kg, i.p.; *n* = 8–11) (**J**), ITT (0.75 IU/kg; *n* = 7–9) (**K**), and OGTT (2 g/kg, gavage; *n* = 6–9) (**L**) following DCZ treatment. Data are mean ± SEM; 2-tailed unpaired *t* test or 2-way ANOVA with Bonferroni’s correction. *P < 0.05, ***P < 0.001.

**Figure 2:**
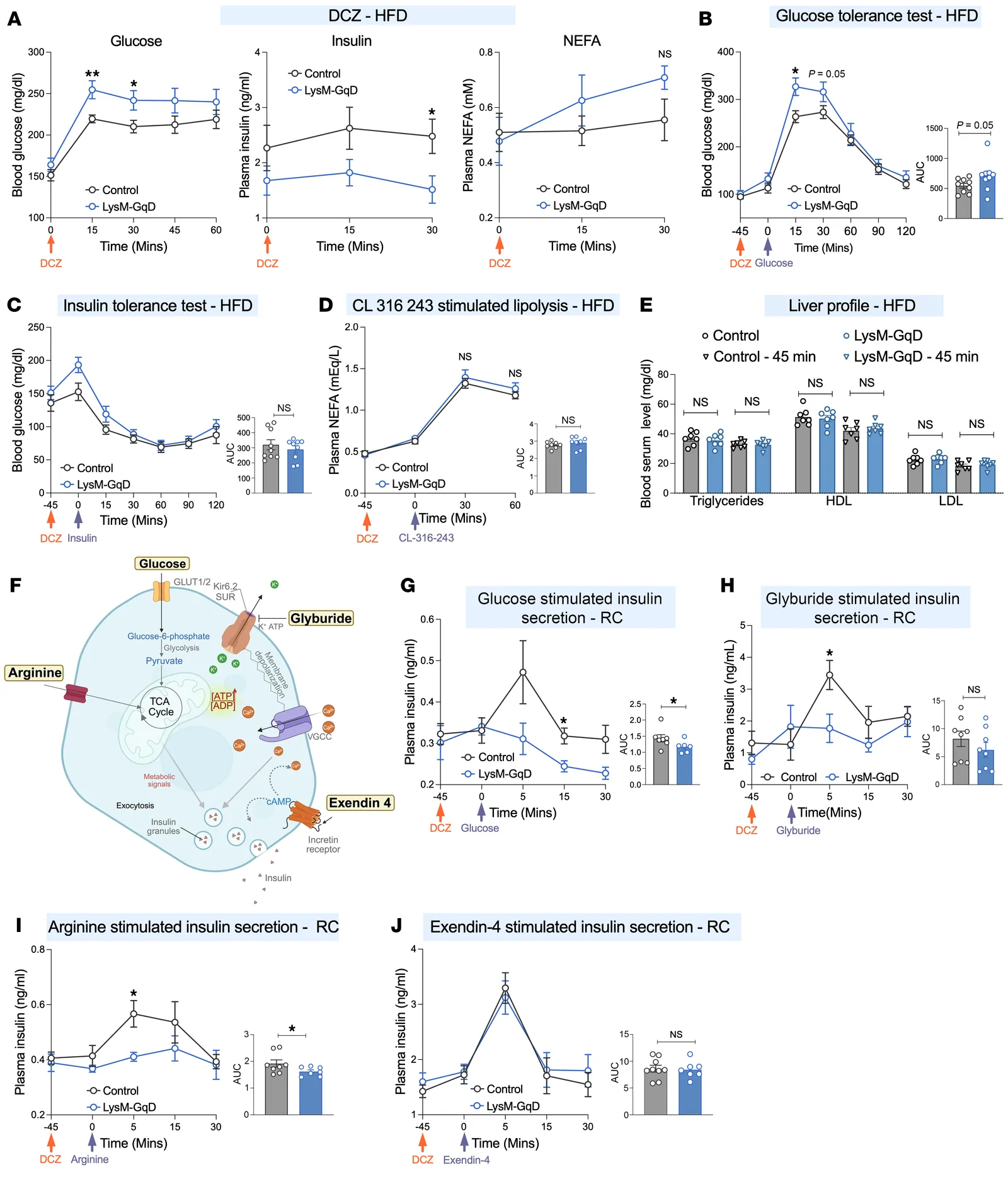
Myeloid GqD activation impairs insulin secretion in vivo and ex vivo. (**A**) Blood glucose, plasma insulin, and NEFAs in HFD-fed (8–12 weeks) LysM-GqD and control mice following DCZ treatment (30 μg/kg, i.p.; *n* = 6–10). (**B**) GTT (1 g/kg; *n* = 9) and (**C**) ITT (1 IU/kg; *n* = 9) with AUC analysis following DCZ treatment. (**D**) CL 316,243 hydrate–stimulated plasma NEFAs after DCZ treatment (*n* = 8). (**E**) Serum triglycerides, HDL, and LDL following DCZ treatment in HFD-fed mice at 8–10 weeks (*n* = 7). (**F**) Schematic of secretagogues used to assess β cell function. (**G**–**J**) Plasma insulin levels in RC-fed control and LysM-GqD mice following acute DCZ administration (30 μg/kg, i.p., 45 min) in response to glucose (2 g/kg, i.p.; *n* = 6–7) (**G**), glyburide (5 mg/kg, i.p.; *n* = 7–8) (**H**), l-arginine (1 g/kg, i.p.; *n* = 7–8) (**I**), or exendin-4 (12 nmol/kg, i.p.; *n* = 7–10) (**J**). Data are mean ± SEM; 2-tailed unpaired *t* test or 2-way ANOVA with Bonferroni’s correction. *P < 0.05, **P < 0.01.

**Figure 3:**
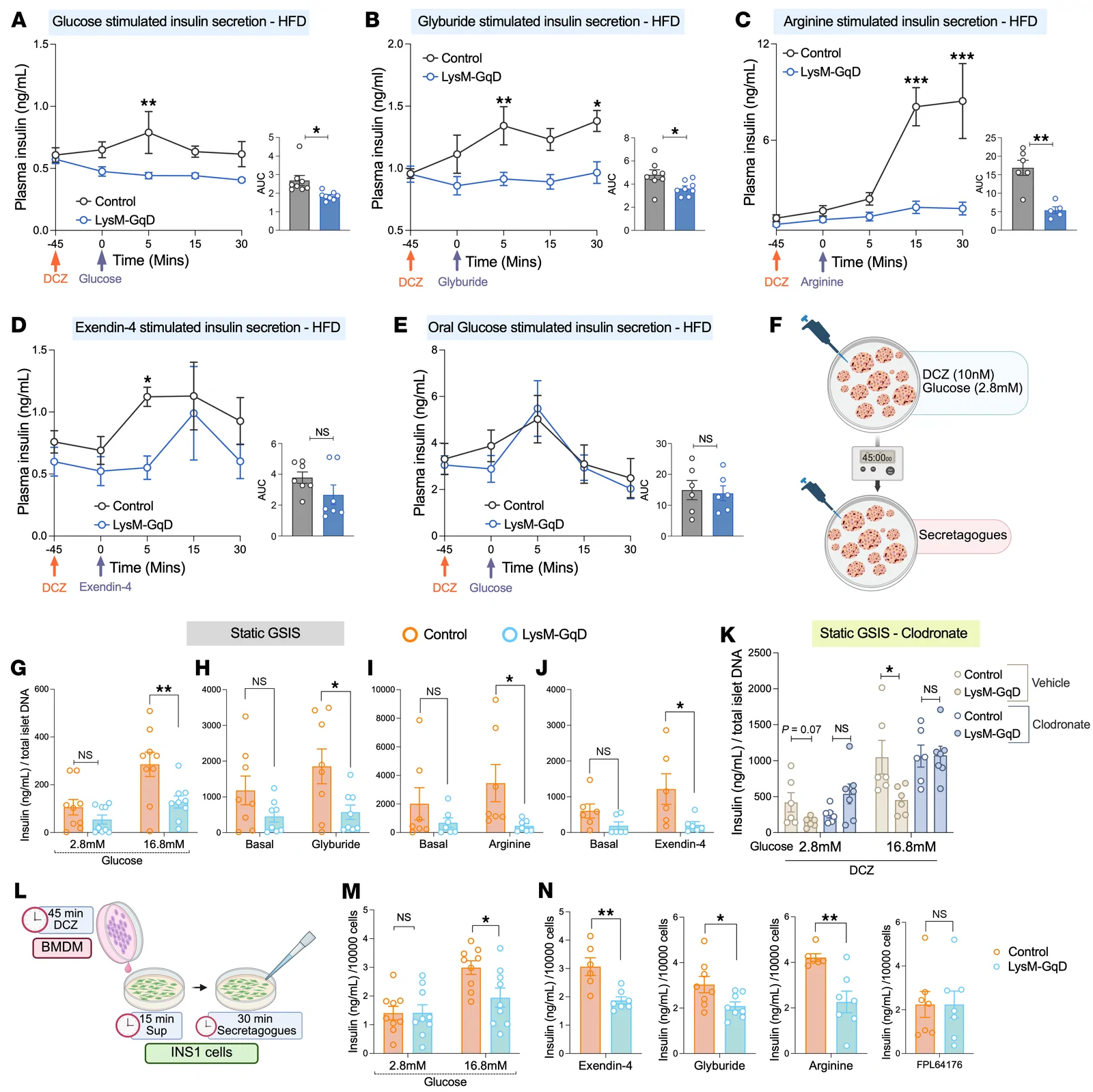
Myeloid Gq activation impairs insulin secretion through macrophage–β cell communication. (**A**–**E**) Plasma insulin levels in HFD-fed (14–16 weeks) control and LysM-GqD mice after acute DCZ stimulation in response to glucose (1 g/kg, i.p.; *n* = 8) (**A**), glyburide (10 mg/kg, i.p.; *n* = 8) (**B**), l-arginine (1 g/kg, i.p.; *n* = 5–6) (**C**), exendin-4 (4 nmol/kg, i.p.; *n* = 7) (**D**), or oral glucose (1 g/kg, *n* = 6) (**E**) with corresponding AUC analyses (right). (**F**) Schematic of the ex vivo islet insulin secretion assay. (**G**–**J**) Static insulin secretion from isolated islets treated with DCZ (30 nM) under low (2.8 mM) and high (16.8 mM) glucose conditions (*n* = 9) (**G**), glyburide (100 nM; *n* = 8) (**H**), l-arginine (30 mM; *n* = 8) (**I**), or exendin-4 (*n* = 6) (**J**), normalized to total islet DNA content. (**K**) Static GSIS in macrophage-depleted islets following clodronate liposome treatment in control and LysM-GqD mice (*n* = 6–7). (**L**) Schematic of indirect BMDM–INS-1 coculture. (**M** and **N**) Insulin secretion from INS-1 cells cocultured with control or LysM-GqD BMDM supernatants in response to DCZ treatment (30 nM) under low (2.8 mM) and high (16.8 mM) glucose (*n* = 9) (**M**), or under exendin-4 (12 nM; *n* = 6), glyburide (100 nM; *n* = 8), l-arginine (30 mM; *n* = 6), or FPL64176 (0.1 μM; *n* = 7) (**N**). Data are mean ± SEM; 2-tailed unpaired *t* test or 2-way ANOVA with Bonferroni’s correction. *P < 0.05, **P < 0.01, ***P < 0.001.

**Figure 4:**
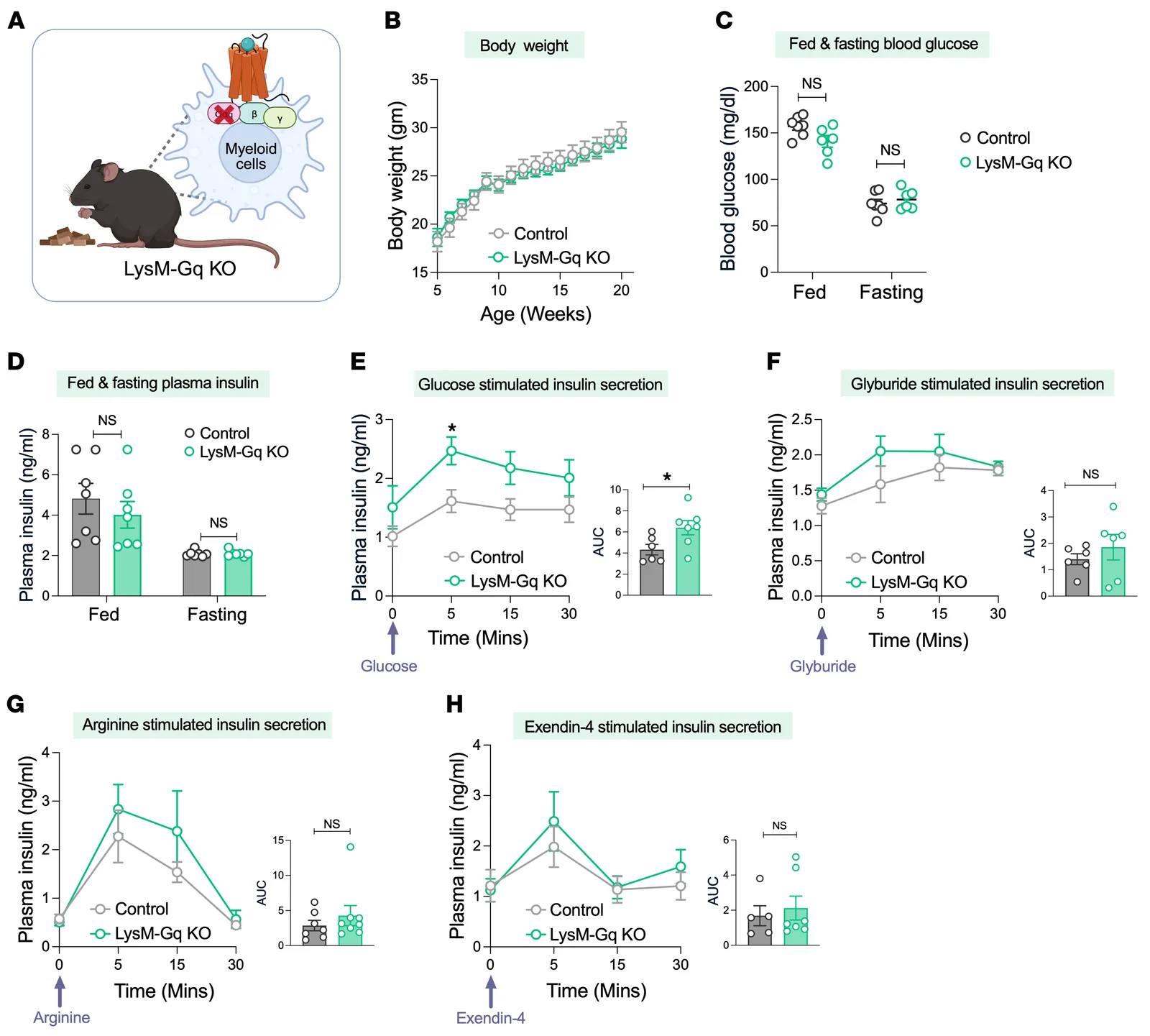
LysM-Gq deletion improves glucose tolerance and insulin secretion. (**A**) Schematic of the LysM-Gq KO model. (**B**–**D**) Body weight (**B**), fed/fasting blood glucose (**C**), and plasma insulin levels (**D**) in RC-fed control and LysM-Gq–KO littermates (*n* = 6–10). (**E**–**H**) Plasma insulin responses and corresponding AUCs following glucose (2 g/kg; *n* = 6–7) (**E**), glyburide (10 mg/kg; *n* = 6) (**F**), l-arginine (1 g/kg; *n* = 8) (**G**), or exendin-4 (12 nmol/kg; *n* = 5–7) (**H**) stimulation in RC-fed control and LysM-Gq–KO mice. Data are mean ± SEM; 2-tailed unpaired *t* test or 2-way ANOVA with Bonferroni’s correction. *P < 0.05.

**Figure 5:**
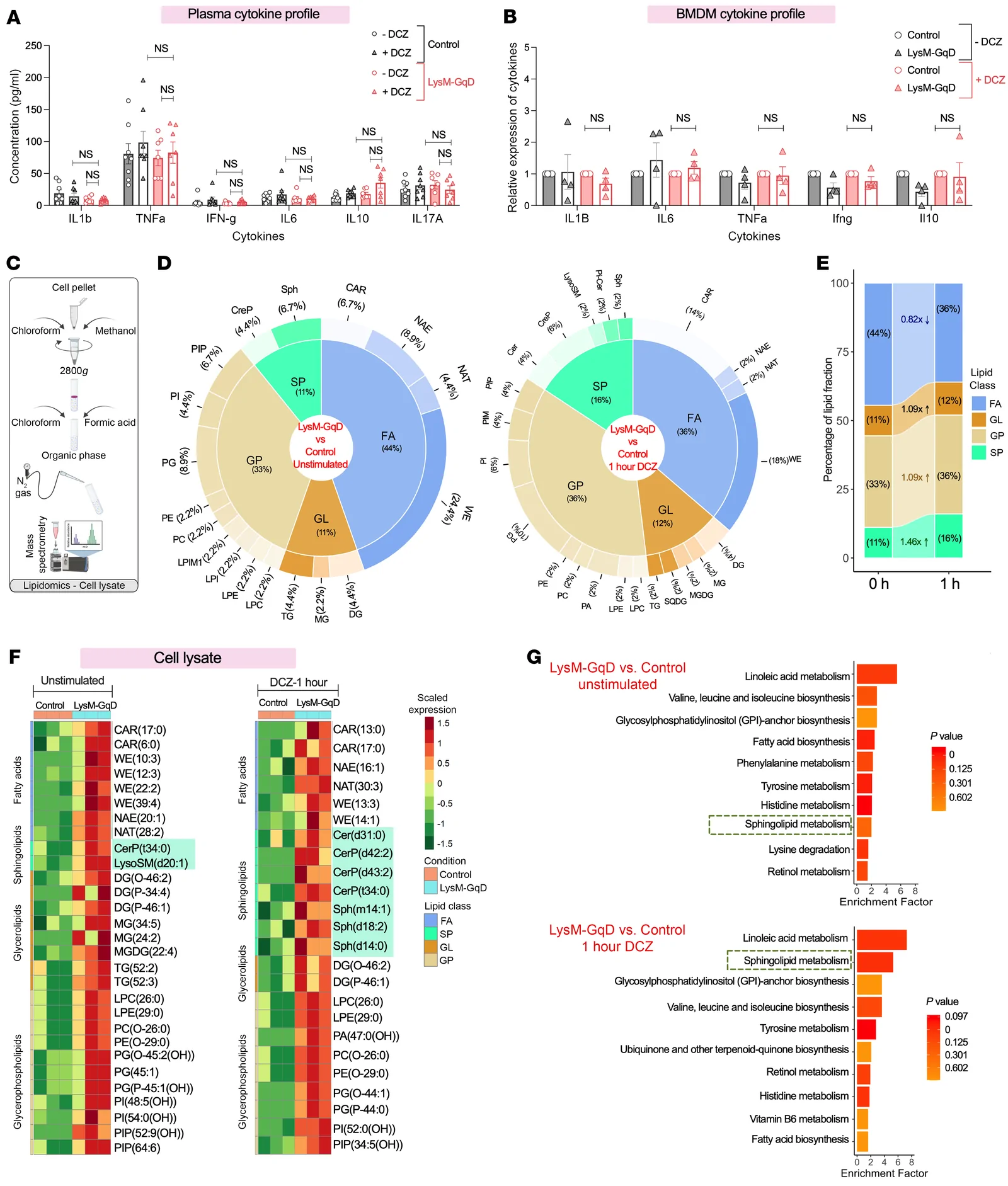
Myeloid Gq activation alters macrophage lipid signaling. (**A** and **B**) Plasma cytokine levels (*n* = 8) (**A**) and BMDM expression (*n* = 4) (**B**) of pro- and antiinflammatory cytokines in control and LysM-GqD mice following 45 min DCZ treatment. (**C**) Workflow for untargeted BMDM lipidomics. (**D**–**F**) Differentially abundant and proportionally increased lipid species (**D** and **E**) and heatmap (**F**) in control and LysM-GqD BMDMs under unstimulated (left) and 1 hour DCZ-stimulated (right) conditions (*n* = 3). (**G**) Pathway enrichment analysis of control and LysM-GqD BMDMs under basal (top) and DCZ-stimulated (bottom) conditions. Data are mean ± SEM; 2-tailed unpaired *t* test or 2-way ANOVA with Bonferroni’s correction. Lipidomic data were analyzed using Student’s *t* test and fold-change analysis in MetaboAnalyst (3 biological × 2 technical replicates). SP, sphingolipids; FA, fatty acids; GP, glycerophospholipids; GL, glycerolipids.

**Figure 6:**
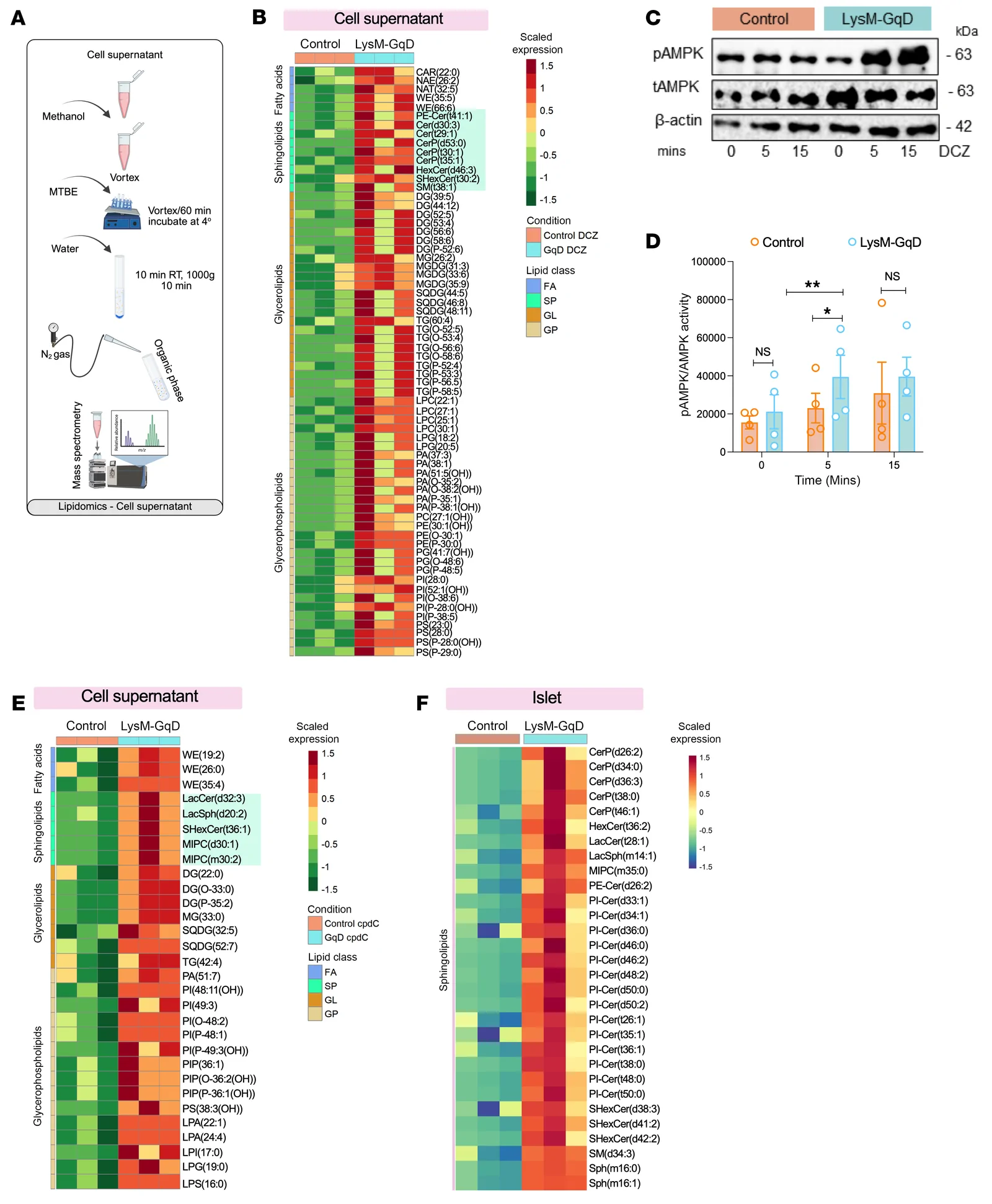
Gq/AMPK signaling drives macrophage lipid remodeling. (**A**) Workflow for untargeted lipidomic analysis of BMDM supernatants. (**B**) Lipidomic heatmaps of control and LysM-GqD BMDM supernatants following 45 min DCZ stimulation (*n* = 3). (**C** and **D**) AMPK phosphorylation following acute GqD activation with DCZ (*n* = 4). (**E**) Lipidomic heatmaps following AMPK inhibition with CpdC in BMDMs. (**F**) Differentially enriched sphingolipid subspecies in DCZ-treated islets from control and LysM-GqD islets (100 islets) (*n* = 3). Data are mean ± SEM; 2-tailed unpaired *t* test or 2-way ANOVA with Bonferroni’s correction. *P < 0.05, **P < 0.01. Lipidomic data were analyzed using Student’s *t* test and fold-change analysis in MetaboAnalyst (3 biological × 2 technical replicates). SP, sphingolipids; FA, fatty acids; GP, glycerophospholipids; GL, glycerolipids.

**Figure 7:**
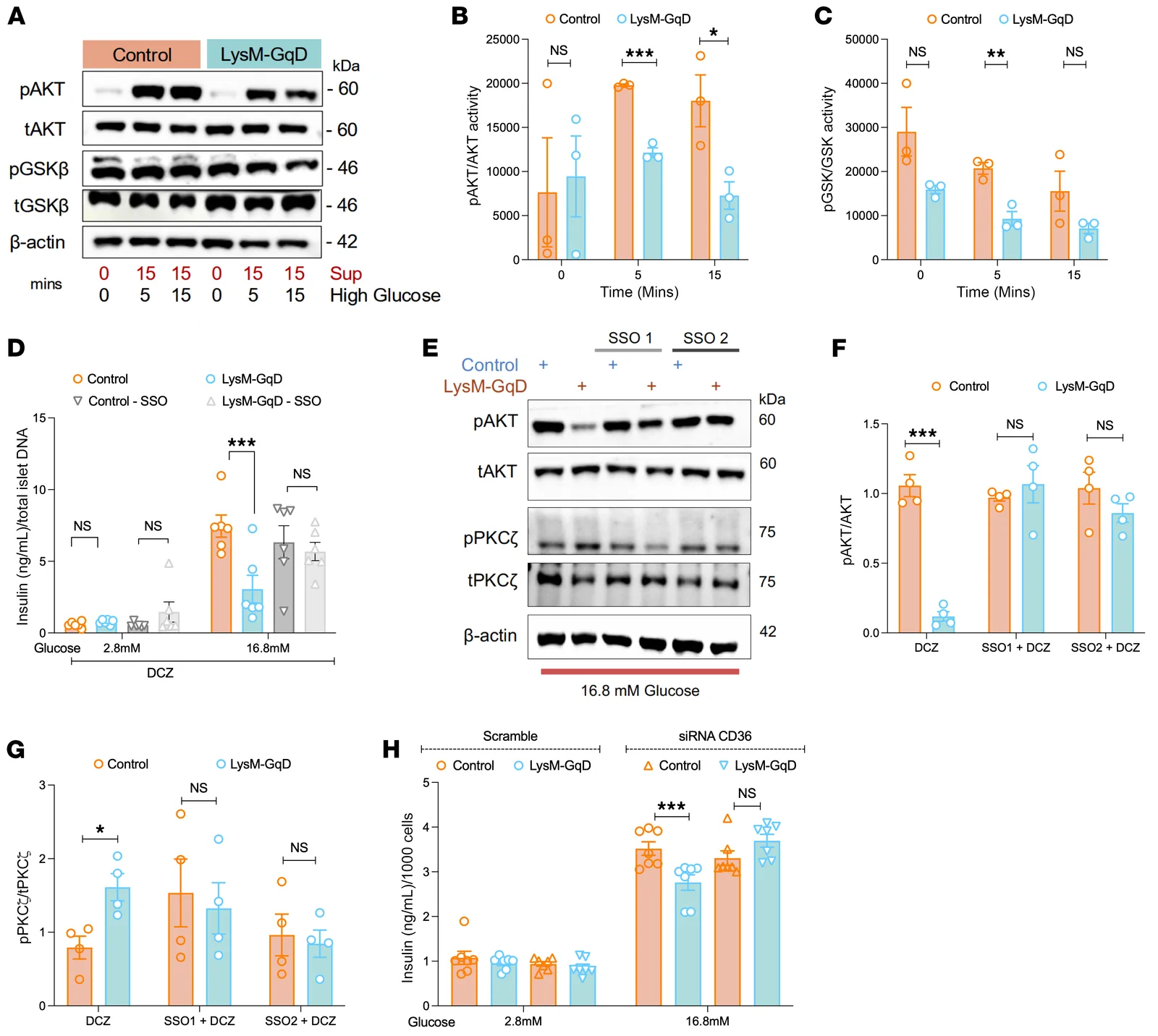
Macrophage-derived sphingolipids impair β cell insulin signaling via CD36. (**A**–**C**) AKT and GSK3-β phosphorylation in INS-1 cells exposed to supernatants from DCZ-stimulated control or LysM-GqD BMDMs under high-glucose conditions (*n* = 3). (**D**) Insulin secretion following exposure to BMDM supernatants with or without the CD36 inhibitor SSO (0.5 mmol/L; *n* = 6). (**E**–**G**) AKT and PKCζ phosphorylation in INS-1 cells following SSO1 (0.1 mmol/L) or SSO2 (0.5 mmol/L) treatment, with corresponding quantification (*n* = 4). (**H**) Insulin secretion following exposure to supernatants from control or LysM-GqD BMDMs with *CD36* siRNA (*n* = 6). Data are mean ± SEM; 2-tailed unpaired *t* test or 2-way ANOVA with Bonferroni’s correction. *P < 0.05, **P < 0.01, ***P < 0.001.

**Figure 8:**
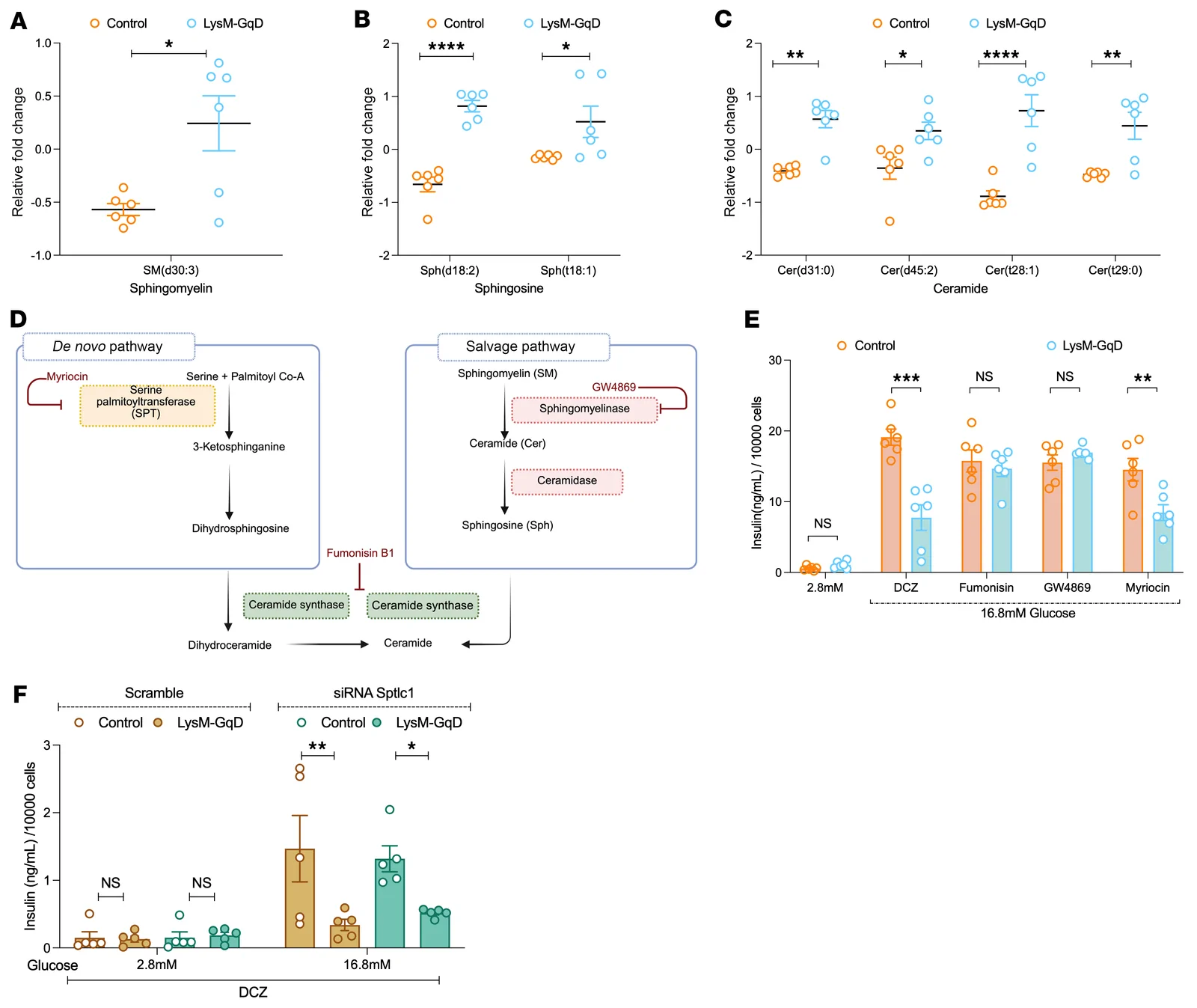
Macrophage-derived sphingolipids suppress β cell insulin secretion. (**A**–**C**) Lipidomic profiling of SM (**A**), Sph (**B**), and Cer (**C**) species in BMDM lysates following 1 hour DCZ treatment. (**D**) Schematic of de novo and salvage sphingolipid biosynthesis pathways. (**E**) Insulin secretion in INS-1 cells under low (2.8 mM) and high (16.8 mM) glucose after transfer of supernatants from control or LysM-GqD BMDMs prestimulated for 24 h with fumonisin B1 (25 μg/mL), GW4869 (10 μM), or myriocin (5 μM) with DCZ (30 nM) (*n* = 6). (**F**) Insulin secretion following exposure to supernatants from DCZ-stimulated BMDMs transfected with scramble or *Sptlc1* siRNA (*n* = 5). Data are mean ± SEM; 2-tailed unpaired *t* test or 2-way ANOVA with Bonferroni’s correction. *P < 0.05, **P < 0.01, ****P < 0.0001

**Figure 9:**
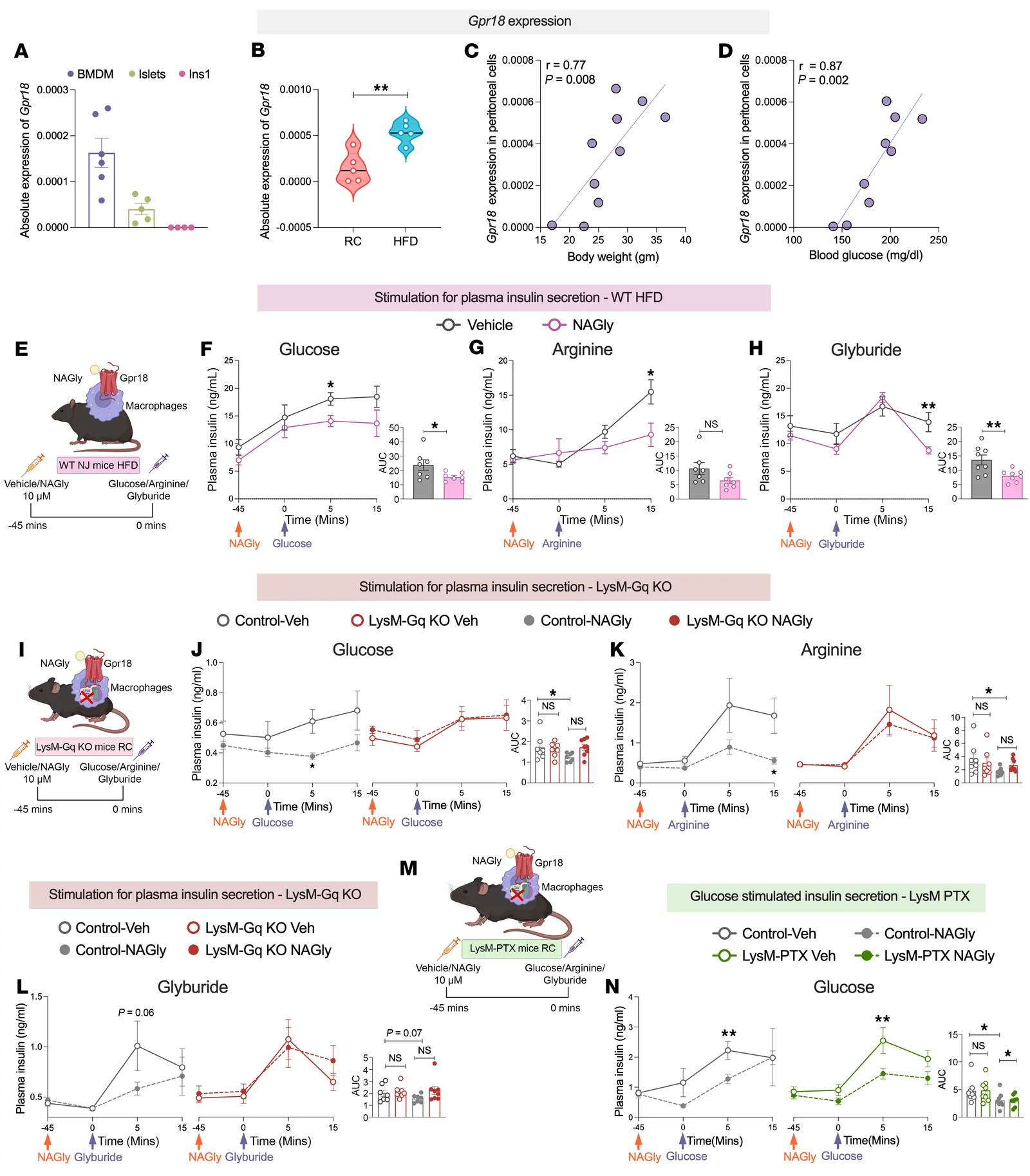
GPR18 suppresses insulin secretion through myeloid Gq signaling. (**A**–**D**) *Gpr18* expression in BMDMs, islets, INS-1 cells (**A**), and peritoneal macrophages (**B**) and its correlation with body weight (**C**) and blood glucose in RC- and HFD-fed mice (**D**). (**E**) Workflow for NAGly injection in NJ mice. (**F**–**H**) Plasma insulin responses to glucose (*n* = 7) (**F**), l-arginine (*n* = 7) (**G**), or glyburide (*n* = 8) (**H**) following GPR18 activation with NAGly (36.15 μg/kg) in HFD-fed WT mice, with corresponding AUC analyses. (**I**) Workflow for NAGly injection in LysM-Gq–KO mice. (**J**–**L**) Insulin responses to NAGly (36.15 μg/kg) in RC-fed control and LysM-Gq–KO mice following glucose (*n* = 7–8) (**J**), l-arginine (*n* = 7–8) (**K**), or glyburide (*n* = 7–8) (**L**) stimulation. (**M**) Workflow for NAGly injection in LysM-PTX (**N**) GSIS following NAGly treatment in RC-fed control and LysM-PTX mice (*n* = 8). Data are mean ± SEM; 2-tailed unpaired *t* test and 2-way ANOVA with Bonferroni’s correction. *P < 0.05, **P < 0.01.

**Figure 10:**
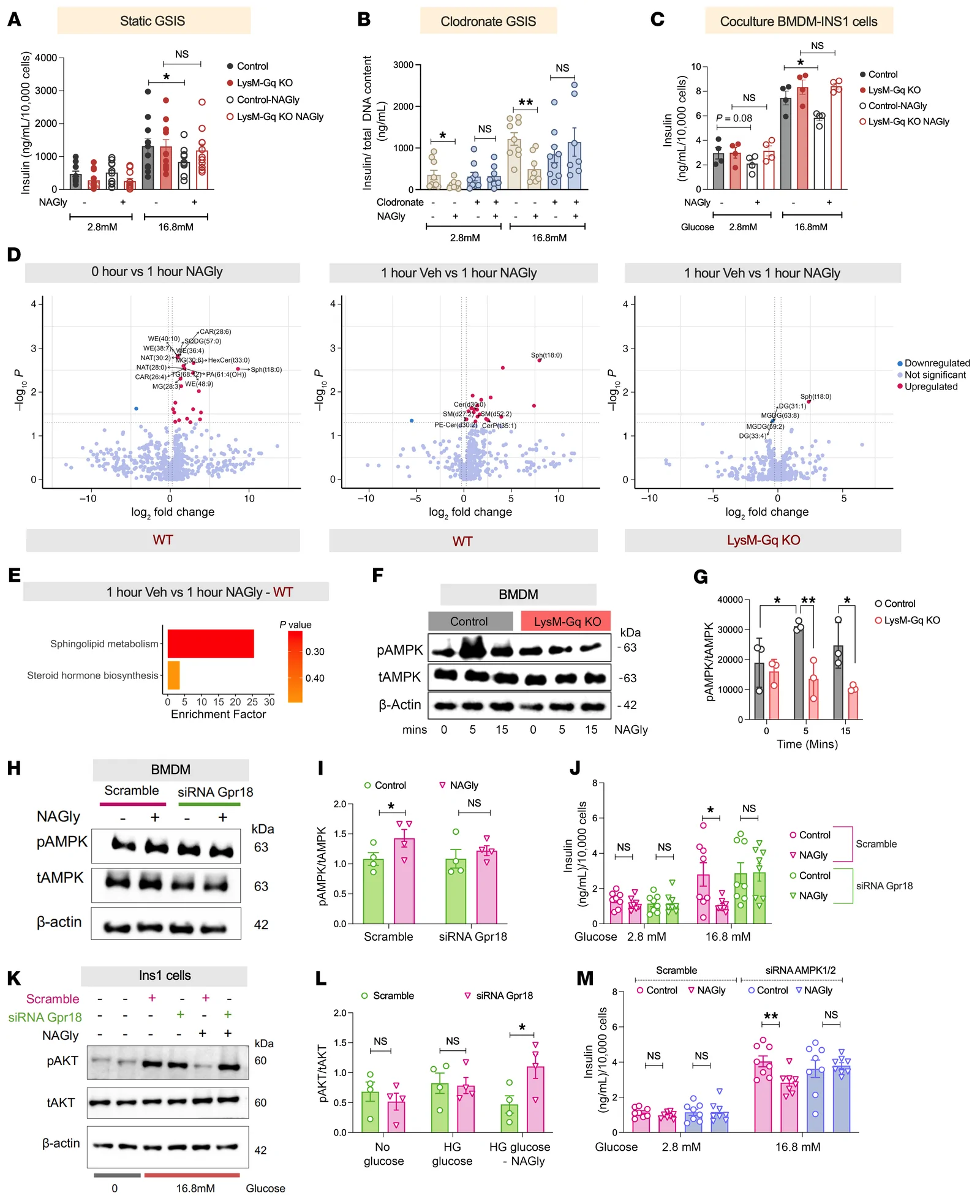
GPR18/Gq/AMPK signaling drives macrophage-mediated β cell dysfunction. (**A**–**C**) GSIS in control and LysM-Gq–KO (*n* = 11–13) (**A**), macrophage-depleted islets (*n* = 7–9) (**B**), and BMDM–INS-1 cocultures (*n* = 4) (**C**) with or without NAGly (10 μM). (**D**–**E**) Lipidomic profiling of WT and LysM-Gq–KO BMDMs following NAGly (10 μM) treatment (**D**), with differential lipid species and pathway enrichment (**E**) highlighting sphingolipid metabolism. (**F** and **G**) NAGly-induced AMPK phosphorylation in control and LysM-Gq–KO BMDMs (*n* = 3). (**H** and **I**) AMPK in BMDMs upon NAGly activation following *Gpr18* siRNA-mediated knockdown (*n* = 4). (**J**) Insulin secretion following NAGly treatment in BMDM–INS-1 cocultures with *Gpr18* knockdown (*n* = 8). (**K** and **L**) AKT phosphorylation in INS-1 cells exposed to BMDM supernatants following NAGly and *Gpr18* knockdown (*n* = 4). (**M**) Insulin secretion in INS-1–BMDM coculture following NAGly activation in AMPK1/2-knockdown BMDMs (*n* = 8). Data are mean ± SEM; 2-tailed unpaired *t* test or 2-way ANOVA with Bonferroni’s correction. *P < 0.05, **P < 0.01. Lipidomic data were analyzed in MetaboAnalyst using unpaired *t* test, Benjamini-Hochberg FDR correction, and fold-change analysis (3 biological × 2 technical replicates).

**Figure 11:**
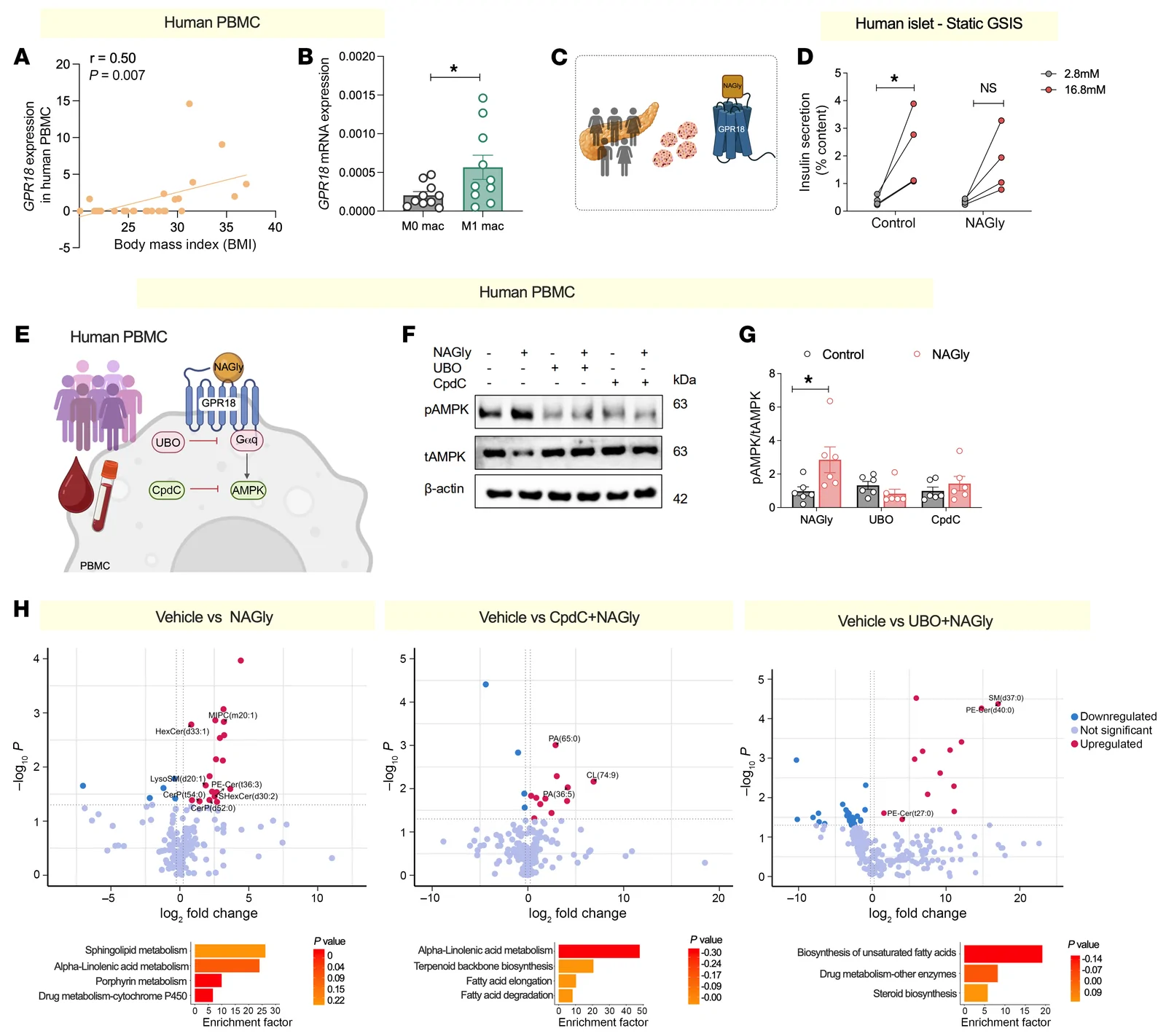
GPR18/Gαq/AMPK signaling regulates sphingolipid metabolism in human macrophages and islets. (**A** and **B**) *GPR18* expression in human macrophages and its association with BMI (*n* = 27) (**A**) and M1 polarization (*n* = 10) (**B**). (**C**) Schematic of human islets and GPR18 activation. (**D**) NAGly-mediated GPR18 activation and low (2.8 mM) and high (16.8 mM) GSIS in human islets (*n* = 4). (**E**) Schematic of Gαq inhibition and AMPK inhibition (CpdC) upon NAGly/Gpr18 axis signaling. (**F** and **G**) Effects of Gαq inhibition and AMPK inhibition (CpdC) on NAGly-induced AMPK phosphorylation in human macrophages (*n* = 6). (**H**) Differential lipid profiling and pathway enrichment following NAGly treatment with or without CpdC or UBO treatment (*n* = 3). Data are mean ± SEM; 2-tailed unpaired *t* test or 2-way ANOVA with Bonferroni’s correction. *P < 0.05. Lipidomic data were analyzed using Student’s *t* test, fold-change analysis, and Benjamini-Hochberg FDR correction (3 biological × 2 technical replicates).

## Version 1 09/15/2026

In-Press Preview

## Version 2 11/16/2026

Electronic publication
