# Supplementary material for: Acute activation of Gq signaling in pancreatic islet macrophages inhibits insulin secretion through AMPK/sphingolipid axis
Source: J Clin Invest. 2026 Sep 15;136(22):e202344. doi: 10.1172/JCI202344 (PMC13767642; doi:10.1172/JCI202344)
Supplement: Supplementary file 1 [file jci-136-202344-s126.pdf]

Acute activation of Gq-signaling in pancreatic islet macrophage inhibits insulin secretion through AMPK-sphingolipid axis

Singh, Kumar et al.
*Corresponding author. Email: pydi@iitk.ac.in

This PDF file includes:

Materials and Methods

Supplemental figure 1 to 9

Resource table 1

Materials and Methods:

Generation and maintenance of mutant mice:

Mutant mice expressing a Gq-coupled DREADD (hM3Dq)(1) was selectively expressed in myeloid cells (LysM-GqD mice) by crossing LysM-Cre mice (The Jackson Laboratory, stock # 018956) with LSL-hM3Dq mice (The Jackson Laboratory, stock # 026220). Mice carrying one copy of the LSL-hM3Dq gene but not the LysM-Cre transgene served as control animals. To generate myeloid-cell-specific Gaq/11 KO mice (LysM-Gq KO), Gna11$^{-/-}$ Gnaq$^{fl/fl}$ mice (gifted by Dr. Stefan Offermanns at University of Heidelberg, Germany) (2, 3) were crossed with LysM-Cre mice, resulting in the experimental cohort of LysM-Cre-Gna11$^{-/-}$Gnaq$^{fl/fl}$ mice. Cre-negative Gna11 $^{-/-}$ Gnaq$^{fl/fl}$ mice were utilized as control mice in all knockout experiments. For selective disruption of Gαi signaling in myeloid cells, ROSA26-PTX$^{fl/fl}$ (RRID:MMRRC_030678-UCD) mice (4, 5), carrying a floxed-stop cassette upstream of the gene encoding the catalytic subunit of pertussis toxin (S1-PTX), were crossed with *LysM-Cre* mice. The resulting *LysM-Cre ROSA26-PTX*$^{flox/+}$ offspring are referred to as *LysM-PTX* mice, with Cre-negative *ROSA26-PTX*$^{flox/+}$ littermates serving as controls. All mouse strains were maintained on a C57BL/6NJ background.

Mice were housed at 23°C under a 12-hour light/dark cycle with ad libitum access to water and regular chow (RC; Safe Diet D131, 12.6 kcal% fat). For HFD studies, 6–8-week-old mice were fed HFD (F3282; 60 kcal% fat, 5.5 kcal/g; Bio-Serv) for ≥8 weeks before metabolic testing.. Body weight and food intake were monitored weekly. Ages and sample sizes are provided in the figure legends.

In vivo metabolic test:

Deschloroclozapine (DCZ) treatment of LysM-GqD mice

LysM-GqD and control littermates maintained on RC or HFD were fasted for 6 hours (8:00 AM–2:00 PM) before receiving DCZ (30 μg/kg, i.p.). Blood glucose was measured from tail-vein blood at 0, 15, 30, 60, 90, and 120 minutes. For plasma insulin and NEFA measurements, blood was collected at 0, 15, 30, and 60 minutes after DCZ administration, and analytes were measured according to the manufacturers' protocols.

Glucose, insulin, and pyruvate tolerance test

GTT, ITT, and PTT were performed in mice ≥8 weeks old maintained on regular chow (RC) or high-fat diet (HFD) for ≥8 weeks. DCZ (30 μg/kg, i.p.) was administered 45 minutes before each test. For RC-fed mice, GTT was also performed with DCZ at 10 μg/kg. GTT was conducted after an overnight fast using glucose (2 g/kg for RC; 1 g/kg for HFD) administered i.p. or by oral gavage. ITT and PTT were performed after a 6-hour fast using insulin (0.75 U/kg

RC; 1 U/kg HFD) or sodium pyruvate (2 g/kg RC; 1 g/kg HFD), respectively. Blood glucose was measured from tail-vein blood using a Contour Next glucometer (Ascensia Diabetes Care) at −45, 0, 15, 30, 60, 90, and 120 minutes.

Glucose/ Glyburide/ Exendin-4/ Arginine/Oral glucose stimulated insulin secretion

All experiments were conducted on mice fasted for six hours (8:00 AM–2:00 PM). DCZ was administered intraperitoneally at a dose of 30 µg/kg body weight 45 minutes prior to the administration (i.p.) of glucose, glyburide, exendin-4, or arginine. For glucose-stimulated insulin secretion (GSIS) and oral GSIS (O-GSIS) assays, RC-fed mice received 2 g/kg glucose and HFD-fed mice received 1 g/kg glucose (6). Glyburide was administered i.p. at 5 mg/kg and 10 mg/kg for RC and HFD groups, respectively(7, 8) . Exendin-4 was given at 12 nmol/kg and 4 nmol/kg for RC and HFD, respectively. Arginine was administered at 1 g/kg body weight for both RC and HFD groups (9). Blood samples were collected from the tail vein into heparin-coated tubes. Plasma was separated by centrifugation at 10,000 × g for 10 minutes at 4 °C. Plasma insulin levels were measured using commercial ELISA kits (Crystal Chem, and ALPCO, USA).

Primary neutrophil culture:

Bone marrow cells were isolated from mouse femurs and tibias by flushing with ice-cold HBSS (Gibco), dissociated, and filtered through a 70-µm cell strainer. Neutrophils were enriched by discontinuous Percoll density-gradient centrifugation using 62% and 75% Percoll at 750 × g for 20 minutes at room temperature without brake. Cells collected from the interface were washed twice and resuspended in HBSS containing 10% fetal bovine serum and 1% penicillin-streptomycin. Cells were incubated for up to 2 hours at 37°C and 5% CO_2 before 45-minute stimulation with DCZ or NAGly for co-culture experiments.

Immunostaining:

Pancreata were harvested following 0.9% saline perfusion and fixed in 4% paraformaldehyde (PFA) at 4°C for 12 hours. Fixed tissues were dehydrated through a graded ethanol series (25%, 50%, 70%, 90%, and 100%), cleared in xylene, and embedded in paraffin. Serial sections (10 µm thick) were cut using a microtome (Leica RM2255) for histological evaluation. For immunofluorescence staining, sections were deparaffinized, rehydrated, and subjected to antigen retrieval as per standard protocols (10). To assess GqD localization in macrophages, sections were stained for HA (GqD) and F4/80, followed by Alexa Fluor 488–anti-rabbit and Alexa Fluor 594–anti-rat secondary antibodies, respectively. Sections were counterstained with DAPI (1:1000) for 2 min at room temperature, washed twice with PBS, and mounted with

ProLong™ Gold antifade reagent (Thermo Fisher Scientific). Images were acquired using a Nikon ECLIPSE Ti2-E inverted fluorescence microscope.

Plasma multiplexed cytokine quantification:

Blood was collected from the tail vein into K2-EDTA tubes and centrifuged at 4°C to obtain plasma. Cytokines were quantified using the Bio-Plex Pro Mouse Th17 Cytokine Panel A 6-Plex assay (Bio-Rad) according to the manufacturer’s instructions. Samples were analyzed on the Luminex xMAP platform using Bio-Plex Manager software, with concentrations calculated from standard curves.

Neutrophil and INS-1 cells culture and coculture:

INS-1 cells (6×10^4/well) and primary neutrophils (0.2×10^4/well) were seeded in 96-well plates. After 6 hours, neutrophils were starved for 60 minutes in KRBH buffer (2.54 mM $CaCl_2$, 1.19 mM $MgSO_4$, 1.199 mM KH_2PO_4, 4.64 mM KCl, 25 mM $NaHCO_3$, and 10 mM HEPES), while INS-1 cells were starved for 15 minutes in basal RPMI containing 1 mM glucose. Neutrophils were treated with DCZ (30 nM) for 45 minutes, after which 75 µL conditioned medium was transferred to INS-1 cells for 15 minutes. Cells were then stimulated for 30 minutes with 2.8 or 16.8 mM glucose in KRBH, and insulin secretion was measured in undiluted supernatants using the ALPCO STELLUX Chemiluminescence ELISA kit.

Western blot analysis:

Islets and cells were lysed in RIPA buffer supplemented with protease and phosphatase inhibitors (Roche 4906845001). Lysates were centrifuged at 15,000 rpm at 4°C for 15 minutes, and protein concentrations were determined using the Pierce BCA assay (Thermo Fisher Scientific). Proteins were separated by SDS-PAGE, transferred to PVDF membranes, blocked with 5% BSA, and incubated overnight at 4°C with primary antibodies (1:1,000), followed by HRP-conjugated secondary antibodies (1:10,000). Blots were developed using Clarity Max ECL (Bio-Rad) and imaged on an Azure system. Densitometry was performed using ImageJ/Fiji.

DEXA scan for mice:

Body composition was determined by dual-energy X-ray absorptiometry (DEXA) using the InAlyzer system (Medikors Inc., Seongnam-si, Republic of Korea). Mice were anesthetized with a ketamine (100 mg/kg) and xylazine (10 mg/kg) cocktail administered intramuscularly, and whole-body scans were acquired according to the manufacturer's protocol. Lean mass and fat mass were quantified using the manufacturer's analysis software.

Indirect calorimetry and energy expenditure measurements:

Energy expenditure measurements were carried out with mice at 23°C using an Oxymax/CLAMS monitoring system (Columbus Instruments). Mice consuming RC and HFD were acclimatized in metabolic chambers for 24 hours and then injected with two injections of DCZ (30 μg/kg, ip) at 8:00 a.m. and 8:00 p.m. the next day. After a single DCZ injection, mice were monitored for one day.

IP1 assay:

Receptor-mediated intracellular changes in IP1 second messenger were measured using IP-One Gq kit (Cisbio). Briefly, BMDM cells were differentiated in 12-well plates as described before. Cells were serum starved for 1 h and treated with NAGly (10 μM) in stimulation buffer (pH 7.4) containing 50 mM LiCl. After 45 min of stimulation, cells were lysed using 200 μL of lysis buffer (HEPES buffer 1×, 1.5% Triton, O.2% BSA). 14 μL of cell lysate was added to the 384-well low volume white microplates (NEST). Working solutions of IP1-d2 followed by Anti-IP1-Cryptate (3 μL) were added to the samples. Plate was sealed and incubated for 1 hour at room temperature. Fluorescence was measured at 620 nm and 665 nm with the BMG plate reader.

PBMC culture:

Human peripheral blood was obtained from healthy donors following Institutional Ethics Committee guidelines at IIT Kanpur. Blood (~20 mL) was collected in heparinized tubes, diluted 1:1 with PBS, and PBMCs were isolated using Histopaque PLUS density-gradient centrifugation (1250 × g, 15 minutes, room temperature, no brake). PBMCs were washed twice with PBS and cultured in RPMI-1640 (Gibco) for Western blotting and lipidomic analyses.

Treatment of mice with a selective Gpr18 receptor agonist:

Mice were fasted for 6 hours (8:00 AM–2:00 PM). NAGly (36.15 μg/kg, i.p.) was administered 45 minutes before glucose, glyburide, exendin-4, or arginine challenge. RC- and HFD-fed mice received glucose at 2 and 1 g/kg, respectively; glyburide at 5 and 10 mg/kg; exendin-4 at 12 and 4 nmol/kg; and arginine at 1 g/kg. Blood was collected into heparin-coated tubes, and plasma was separated (10,000 × g, 10 minutes, 4°C) and stored at −80°C. Plasma insulin was measured using commercial ELISA kits (Crystal Chem and ALPCO, USA).

1. Armbruster BN, Li X, Pausch MH, Herlitze S, and Roth BL. Evolving the lock to fit the key to create a family of G protein-coupled receptors potently activated by an inert ligand. *Proc Natl Acad Sci U S A.* 2007;104(12):5163–8.

2. Regard JB, Kataoka H, Cano DA, Camerer E, Yin L, Zheng Y-W, et al. Probing cell type–specific functions of Gi in vivo identifies GPCR regulators of insulin secretion. *The Journal of Clinical Investigation.* 2007;117(12):4034–43.
3. Offermanns S, and Simon MI. Genetic analysis of mammalian G-protein signalling. *Oncogene.* 1998;17(11 Reviews):1375–81.
4. Regard JB, Kataoka H, Cano DA, Camerer E, Yin L, Zheng YW, et al. Probing cell type-specific functions of Gi in vivo identifies GPCR regulators of insulin secretion. *J Clin Invest.* 2007;117(12):4034–43.
5. Rossi M, Zhu L, McMillin SM, Pydi SP, Jain S, Wang L, et al. Hepatic Gi signaling regulates whole-body glucose homeostasis. *J Clin Invest.* 2018;128(2):746–59.
6. Kimura T, Pydi SP, Wang L, Haspula D, Cui Y, Lu H, et al. Adipocyte Gq signaling is a regulator of glucose and lipid homeostasis in mice. *Nature Communications.* 2022;13(1):1652.
7. Aamodt KI, and Powers AC. Signals in the pancreatic islet microenvironment influence β-cell proliferation. *Diabetes Obes Metab.* 2017;19 Suppl 1(Suppl 1):124–36.
8. Barella LF, Rossi M, Zhu L, Cui Y, Mei FC, Cheng X, et al. β-Cell-intrinsic β-arrestin 1 signaling enhances sulfonylurea-induced insulin secretion. *J Clin Invest.* 2019;129(9):3732–7.
9. Cheng K, Delghingaro-Augusto V, Nolan CJ, Turner N, Hallahan N, Andrikopoulos S, et al. High Passage MIN6 Cells Have Impaired Insulin Secretion with Impaired Glucose and Lipid Oxidation. *PLOS ONE.* 2012;7(7):e40868.
10. Liu L, El K, Dattaroy D, Barella LF, Cui Y, Gray SM, et al. Intra-islet α-cell Gs signaling promotes glucagon release. *Nature Communications.* 2024;15(1):5129.

Resource Table: 1

**Reagent or Resource**	**Source**	**Cat #**
Antibodies		
Rabbit anti-pAMPK Ab	Cell Signaling Technology	2535 (1:1000)
Rabbit anti-AMPK Ab	Cell Signaling Technology	2532(1:1000)
Rabbit anti-pAKT Ab	Cell Signaling Technology	9271(1:1000)
Rabbit anti-AKT Ab	Cell Signaling Technology	9272(1:1000)
Rabbit anti-pGSKβ Ab	Cell Signaling Technology	5558(1:1000)
Rabbit anti-GSKβ Ab	Cell Signaling Technology	9315(1:1000)
Rabbit anti-pPKCZ Ab	Cell Signaling Technology	32450(1:1000)
Rabbit anti-PKCZ Ab	Invitrogen	PA5-78129(1:1000)
Rabbit anti-β actin Ab	Proteintech	81115-1-RR(1:1000)
Rabbit anti-HA Ab	Cell Signaling Technology	3724 (1:1000)
Rat-F4/80 Ab	Abcam	ab6640
Rabbit anti-Ki67 Ab	Cell Signaling Technology	9129
Goat Anti-Rabbit Alexa Fluor 488	Invitrogen	A32731
Anti-rabbit IgG, HRP-linked secondary Ab	Cell Signaling Technology	7074 (1:10,000)
DAPI	SRL	80145
Compound		
Deschloroclozapine (DCZ)	Hello Bio Inc	HB8555
Human insulin (HUMINSULIN 30/70 100IU)	Eli Lilly	HI0719
Disodium Clodronate Tetrahydrate	Tokyo Chemical Industry Co. LTD.	D4160
Glyburide (potassium salt)	Cayman Chemical	15009
Exendin-4 (48-86) amide (acetate)	Cayman Chemical	11096
L-Arginine	Sigma Aldrich	A5006
FPL-6417	Cayman Chemical	20122
CL-316-243	Sigma Aldrich	C5976
RPMI 1640 media, no glucose	Gibco	11879020
Recombinant Human M-CSF	PeproTech	300-25-100UG

cOmplete, EDTA-free protease inhibitor cocktail	MilliporeSigma	11697498001
Sulfosuccinimidyl Oleate (Sodium salt)	Cayman Chemical	11211
UBO-QIC	Cayman Chemical	33666
Arachidonoyl Glycine (NAGly)	Cayman Chemical	90051
Dorsomorphin(hydrochloride) - CompoundC	Cayman Chemical	21207
Collagenase	Sigma Aldrich	C7657
Kits		
Ultra-Sensitive Mouse Insulin ELISA kit (Plasma)	Crystal Chem	90082
Rodent Insulin Chemiluminescence ELISA (*Ex vivo*)	ALPCO	80-INSMR-CH01
U-Plex Mouse Insulin assay	Meso Scale Discovery	K1526HK-2
ReliaPrep™ RNA Miniprep Systems	Promega	Z6010
PrimeScript 1st strand cDNA Synthesis Kit	Takara	6110A
HTRF IP-One Gq Detection Kit	Revvity	62IPAPEB
BCA protein assay kit	Thermo Fisher Scientific	23225
LabAssayTM NEFA（FFA）	Fujifilm	299-94301
Quant-iT™ PicoGreen™ dsDNA Assay Kits and dsDNA Reagents	Thermofisher Scientific	P11496
TUNEL Apoptosis Detection Kit	MedChemExpress	HY-K1078
Experimental Models: Cell Lines		
INS-1 cells	Gifted by Dr. Prosenjit Mondal	
Experimental Models: Mouse Strains		
C57BL/6N mice (WT mice)	The Jackson Laboratory	005304

R26-hM3Dq/mCitrine	The Jackson Laboratory	026220
B6N.129P2(B6)-Lyz2tm1(cre)Ifo/J	The Jackson Laboratory	018956
Gnaq $^{flox/flox}$;Gna11$^{-/-}$	Dr. Stefan Offermanns (University of Heidelberg, Germany)	
ROSA26-PTX$^{fl/fl}$	MMRRC-UCD	MMRRC_030678-UCD
Oligomers		
hM3Dq_Mutant Forward	CGCCACCATGTACCCATC	Eurofins
hM3Dq_Mutant Reverse	GTGGTACCGTCTGGAGAG GA	Eurofins
hM3Dq_ Wild type Forward	AAGGGAGCTGCAGTGGAGTA	Eurofins
hM3Dq_ Wild type Reverse	CCGAAAATCTGTGGGAAGTC	Eurofins
Lyz2tm1(cre)Ifo/J_ Mutant Reverse	CCCAGAAATGCCAGATTACG	Eurofins
Lyz2tm1(cre)Ifo/J_Common	CTTGGGCTGCCAGAATTTCTC	Eurofins
Lyz2tm1(cre)Ifo/J_ Wild type Reverse	TTACAGTCG GCCAGGCTG AC	Eurofins
Genotyping primers		
hm3Dq_Forward	AGAAGAACGGCATCAAGGTG	Eurofins
hm3Dq_Reserve	GAACTCCAGCAGGACCATGT	Eurofins
Gnaq $^{flox/flox}$; _Forward	AGCTTAGTCTGGTGACAGAAGC	Eurofins
Gnaq $^{flox/flox}$_ Reverse	GCATGCGTGTCCTTTATGTGAG	Eurofins
qPCR primers		
Gnaq _Forward (Mouse)	TGGACCGTGTAGCCGACCCT	Eurofins
Gnaq _ Reverse (Mouse)	GGCCCCCTACATCGACCATTCTGA	Eurofins
Gnaq _Forward (Human)	TGGGTCAGGATACTCTGATGAAG	Eurofins
Gnaq _ Reverse (Human)	TGTGCATGAGCCTTATTGTGC	Eurofins
Gna11 _Forward (Mouse)	CTACTTGGCACTGGCGAGAG	Eurofins
Gna11 _ Reverse (Mouse)	GTCACTGGACAGCGTGTTGA	Eurofins
Gna11 _Forward (Human)	TCATCGAGTACCCTTTCGACC	Eurofins
Gna11 _ Reverse (Human)	GGTACTCTCGGGATCTGTTGAAA	Eurofins
Gpr18 _Forward (Mouse)	CACCCTGAGCAATCACAACCA	Eurofins
Gpr18 _ Reverse (Mouse)	AGTGACATTAACAAACAGCCCA	Eurofins
GPR18_Forward (Human)	CGCCACCTGCCTCAAGATTT	Eurofins
GPR18 _ Reverse (Human)	TGACCAAGTAGCACCCAATCAT	Eurofins

siRNA		
AMPK1/2 siRNA - Mouse	Santa Cruz	sc-45313
Gpr18 siRNA - Mouse	Medchem Express	HY- 17979
Sptlc1 siRNA - Mouse	Medchem Express	HY-RS21943
CD36 siRNA - Rat	Santa Cruz	sc-270644
Software and algorithms		
Prism 10	Graph Pad	https://www.graphpad.com/scientificsoftware/prism
R		RRID: SCR_001905
Seurat		RRID: SCR_007322

Figure S1

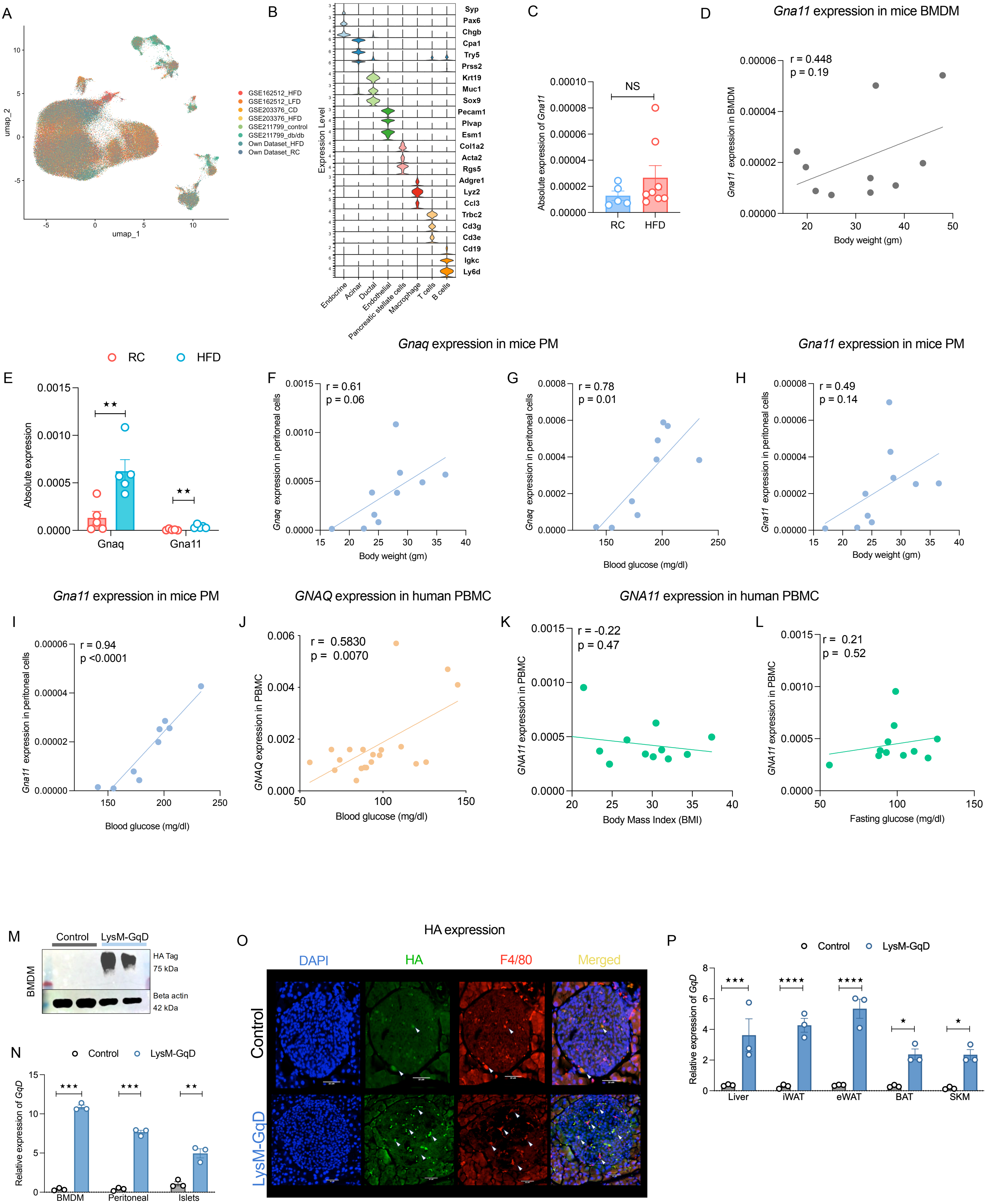
A
umap_1
umap_2
GSE162512_HFD
GSE162512_LFD
GSE203376_CD
GSE203376_HFD
GSE211799_control
GSE211799_db/db
Own Dataset_HFD
Own Dataset_RC
B
Expression Level
Syp
Pax6
Chgb
Cpa1
Try5
Prss2
Krt19
Muc1
Sox9
Pecam1
Plvap
Esm1
Col1a2
Acta2
Rgs5
Adgre1
Lyz2
Ccl3
Trbc2
Cd3g
Cd3e
Cd19
Igkc
Ly6d
Endocrine
Acinar
Ductal
Endothelial
Pancreatic stellate cells
Macrophage
T cells
B cells
C
Absolute expression of Gna11
NS
RC
HFD
D
Gna11 expression in mice BMDM
r = 0.448
p = 0.19
Gna11 expression in BMDM
Body weight (gm)
E
RC
HFD
Absolute expression
Gnaq
Gna11
Gnaq expression in mice PM
F
r = 0.61
p = 0.06
Gnaq expression in peritoneal cells
Body weight (gm)
G
r = 0.78
p = 0.01
Gnaq expression in peritoneal cells
Blood glucose (mg/dl)
Gna11 expression in mice PM
H
r = 0.49
p = 0.14
Gna11 expression in peritoneal cells
Body weight (gm)
Gna11 expression in mice PM
I
r = 0.94
p <0.0001
Gna11 expression in peritoneal cells
Blood glucose (mg/dl)
GNAQ expression in human PBMC
J
r = 0.5830
p = 0.0070
GNAQ expression in PBMC
Blood glucose (mg/dl)
GNA11 expression in human PBMC
K
r = -0.22
p = 0.47
GNA11 expression in PBMC
Body Mass Index (BMI)
L
r = 0.21
p = 0.52
GNA11 expression in PBMC
Fasting glucose (mg/dl)
M
Control
LysM-GqD
BMDM
HA Tag
75 kDa
Beta actin
42 kDa
N
Control
LysM-GqD
Relative expression of GqD
BMDM
Peritoneal
Islets
O
HA expression
DAPI
HA
F4/80
Merged
Control
LysM-GqD
P
Control
LysM-GqD
Relative expression of GqD
Liver
iWAT
eWAT
BAT
SKM

Supplemental Figure 1. ***Gαq/11*** **expression in mouse macrophages and human PBMCs.** (**A**) Integrated UMAP of scRNA-seq data from murine islets under RC, HFD, and db/db conditions. (**B**) Major immune cell subsets (macrophages, T cells, B cells) across RC, HFD, and db/db mice, represented as percentage of population. (**C**) *Gna11* expression in bone marrow–derived macrophages (BMDMs) from RC and HFD mice (n=5-8). (**D**) Correlation of *Gna11* expression in BMDM with body weight (n=10). (**E**) Expression of *Gnaq* and *Gna11* in PMs from RC versus HFD mice (n=5). (**F-G**) Correlation of *Gnaq* expression in PMs with (**F**) body weight and (**G**) blood glucose (n=9-10). (**H-I**) Correlation of *Gna11* expression in PMs with (**H**) body weight and (**I**) blood glucose (n=9-10). (**J-L**) Human PBMC analysis: *GNAQ* expression correlated with blood glucose (n=20)(**J**), and *GNA11* expression with body mass index (BMI) (**K**) and fasting glucose (**L**) (n=11-12). (**M**) GqD expression in islets by immunoblot (n=2–3). (**N**) GqD expression in BMDMs, peritoneal macrophages, and islets by qPCR. (**O**) Immunofluorescence of pancreatic islets showing HA-tagged GqD in F4/80$^+$ macrophages (DAPI, blue; F4/80, red; HA, green), scale bar = 20μm (n=3). (**P**) Relative expression of *GqD* in liver, iWAT (inguinal white adipose tissue), eWAT (epididymal white adipose tissue), BAT (brown adipose tissue), SKM (skeletal muscle) isolated from control and LysM-GqD on 8 weeks of HFD (n=3). Data represented as mean ± s.e.m.; Pearson's correlation, two-tailed unpaired t-test and two-way ANOVA Bonferroni multiple comparison test. *p < 0.05, **p < 0.01, ***p < 0.001; NS = not significant.

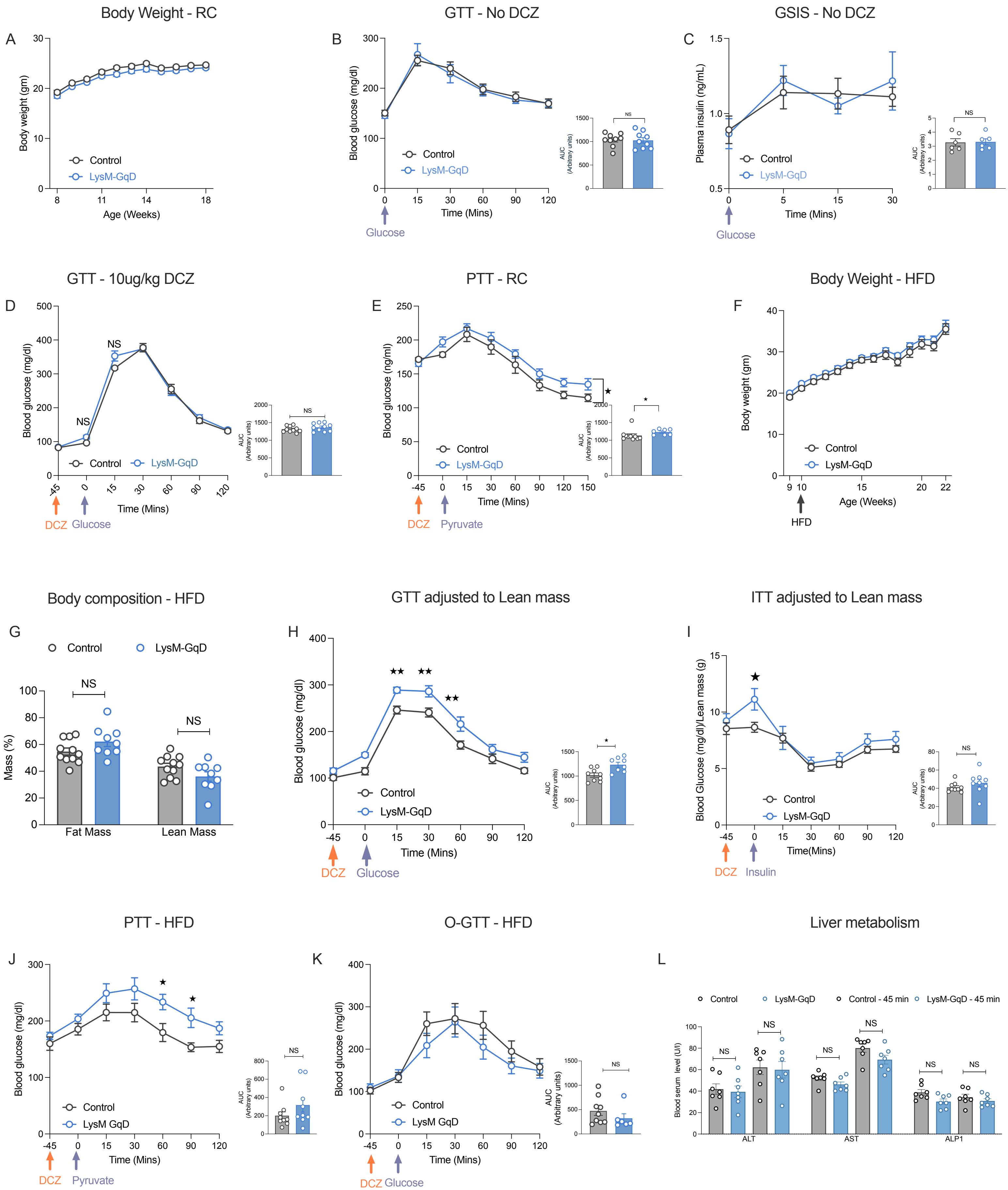

Body Weight - RC
A
Body weight (gm)
Control
LysM-GqD
Age (Weeks)
GTT - No DCZ
B
Blood glucose (mg/dl)
Control
LysM-GqD
Time (Mins)
Glucose
AUC (Arbitrary units)
NS
GSIS - No DCZ
C
Plasma insulin (ng/mL)
Control
LysM-GqD
Time (Mins)
Glucose
AUC (Arbitrary units)
NS
GTT - 10ug/kg DCZ
D
Blood glucose (mg/dl)
NS
NS
Control
LysM-GqD
Time (Mins)
DCZ
Glucose
AUC (Arbitrary units)
NS
PTT - RC
E
Blood glucose (ng/ml)
Control
LysM-GqD
Time (Mins)
DCZ
Pyruvate
AUC (Arbitrary units)
*
Body Weight - HFD
F
Body weight (gm)
Control
LysM-GqD
Age (Weeks)
HFD
Body composition - HFD
G
Control
LysM-GqD
Mass (%)
NS
NS
Fat Mass
Lean Mass
GTT adjusted to Lean mass
H
Blood glucose (mg/dl)
Control
LysM-GqD
Time (Mins)
DCZ
Glucose
AUC (Arbitrary units)
*
ITT adjusted to Lean mass
I
Blood Glucose (mg/dl)/Lean mass (g)
Control
LysM-GqD
Time(Mins)
DCZ
Insulin
AUC (Arbitrary units)
NS
PTT - HFD
J
Blood glucose (mg/dl)
Control
LysM GqD
Time (Mins)
DCZ
Pyruvate
AUC (Arbitrary units)
NS
O-GTT - HFD
K
Blood glucose (mg/dl)
Control
LysM GqD
Time (Mins)
DCZ
Glucose
AUC (Arbitrary units)
NS
Liver metabolism
L
Control
LysM-GqD
Control - 45 min
LysM-GqD - 45 min
Blood serum level (U/l)
NS
ALT
AST
ALP1

Supplemental Figure 2. Glucose homeostasis and hormone regulation in LysM-GqD and control mice under regular chow (RC) and high-fat diet (HFD) conditions. (**A**) Body weight of LysM-GqD and control mice maintained on RC (n=7-11). (**B**) Intraperitoneal glucose tolerance test (GTT) (n=9) and (**C**) Glucose-stimulated insulin secretion (GSIS) in RC-fed LysM-GqD and control mice without DCZ stimulation (n=6). (**D**) GTT in RC-fed LysM-GqD and control mice after acute DCZ (10 μg/kg) stimulation (n=10). (**E**) Pyruvate tolerance test (PTT) in RC-fed LysM-GqD and control mice with DCZ (30 μg/kg) stimulation (n=7-11). (**F**) Body weight progression of LysM-GqD and control mice under 8-12 weeks HFD (n=9-11). (**G**) Body composition analysis of control and LysM-GqD mice after 12 weeks of high-fat diet (HFD) feeding. Lean mass and fat mass are expressed as percentages of total body weight. (**H**) Intraperitoneal GTT in control and LysM-GqD mice after 12 weeks of HFD feeding. Glucose was administered based on lean body mass. (**I**) ITT in control and LysM-GqD mice following 12 weeks of HFD feeding. Insulin (1 U/kg, i.p.) was administered with dosing normalized to lean body mass. (**J**) PTT in HFD-fed LysM-GqD and control mice with DCZ (30 μg/kg) stimulation (n=9). (**K**) Oral glucose tolerance test (OGTT) in 8-10 weeks HFD-fed LysM-GqD and control mice with DCZ (30 μg/kg) stimulation (n=6-9). (**L**) Serum ALT, AST and ALP1 levels in HFD-fed (12 weeks) LysM-GqD and control mice following acute DCZ administration (30 μg/kg, i.p.; n = 7). Data are shown as mean ± s.e.m. using two-tailed unpaired t-test and two-way ANOVA Bonferroni multiple comparison test. *p < 0.05, **p < 0.01, ***p < 0.001; NS = not significant.

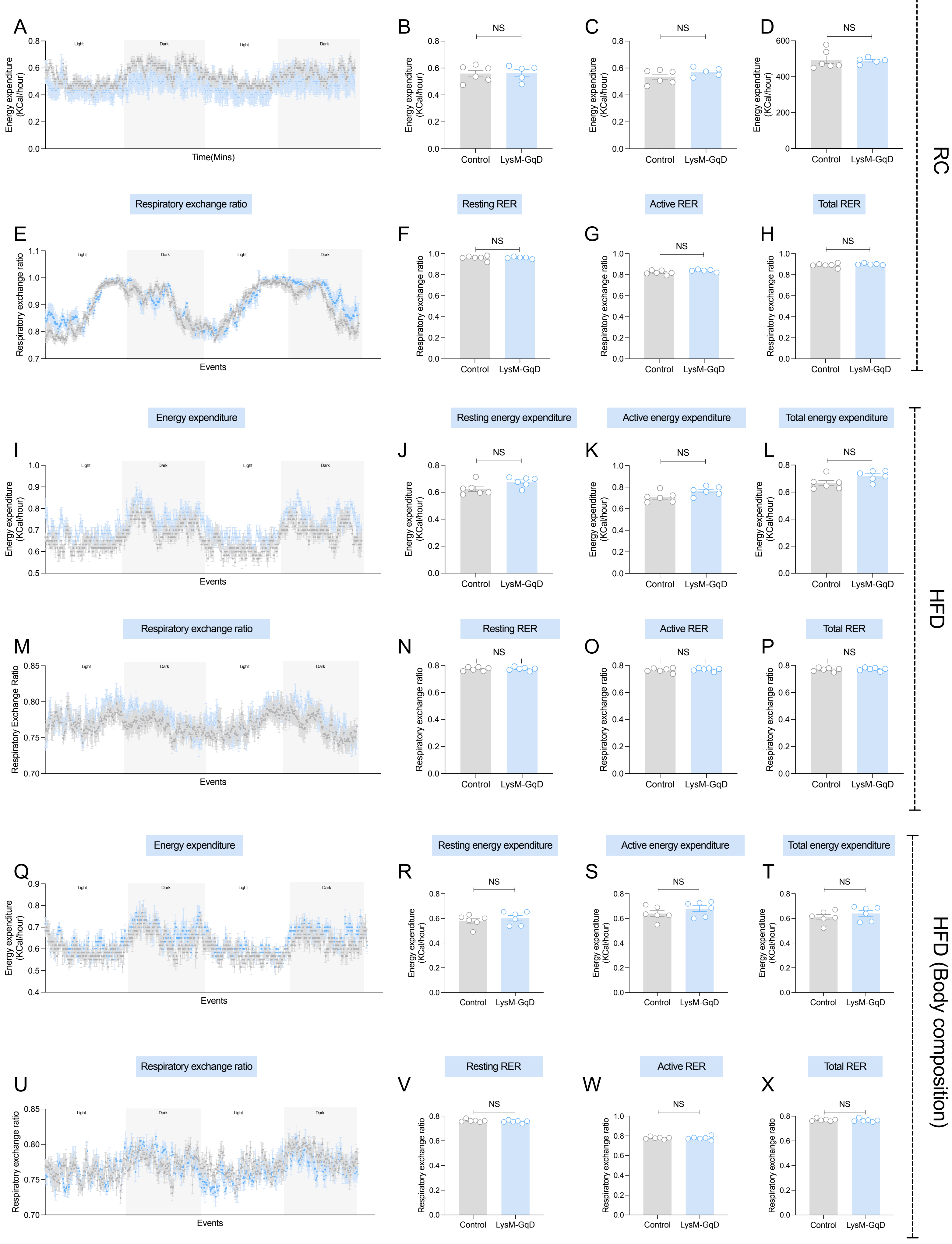

A
B
C
D
Energy expenditure (KCal/hour)
Time(Mins)
Light
Dark
NS
Control
LysM-GqD
RC
Respiratory exchange ratio
Resting RER
Active RER
Total RER
E
F
G
H
Events
Energy expenditure
Resting energy expenditure
Active energy expenditure
Total energy expenditure
I
J
K
L
HFD
M
N
O
P
Respiratory Exchange Ratio
Q
R
S
T
HFD (Body composition)
U
V
W
X

Supplemental Figure 3. Myeloid Gq activation does not alter whole-body energy expenditure or substrate utilization both in RC and HFD condition. (**A–D**) Energy expenditure in RC-fed mice: (**A**) Time-course of total energy expenditure across light and dark cycles (n=5-6); (**B–D**) Quantification of resting, active, and total energy expenditure showing no significant differences between genotypes (n=5-6). (**E–H**) RER in RC-fed mice: (**E**) RER traces across light and dark cycles; (**F–H**) Quantification of resting, active, and total RER, indicating comparable substrate utilization between groups. (**I–L**) Energy expenditure in HFD-fed mice: (**I**) Time-course of total energy expenditure; (**J–L**) Quantification of resting, active, and total energy expenditure showing no genotype-dependent differences. (**M–P**) RER in HFD-fed mice (n=6): (**M**) RER time-course across light and dark cycles; (**N–P**) Quantification of resting, active, and total RER, with no significant differences between groups. (**Q–T**) Energy expenditure in HFD-fed mice with no difference in fat and lean mass: (**Q**) Time-course of total energy expenditure; (**R–T**) Quantification of resting, active, and total energy expenditure showing no genotype-dependent differences. (**U–X**) RER in HFD-fed mice (n=6): (**U**) RER time-course across light and dark cycles; (**V–X**) Quantification of resting, active, and total RER, with no significant differences between groups. Data are shown as mean ± s.e.m. using two-tailed unpaired t-test and two-way ANOVA Bonferroni multiple comparison test. $^{*}p < 0.05$, $^{**}p < 0.01$, $^{***}p < 0.001$; NS = not significant.

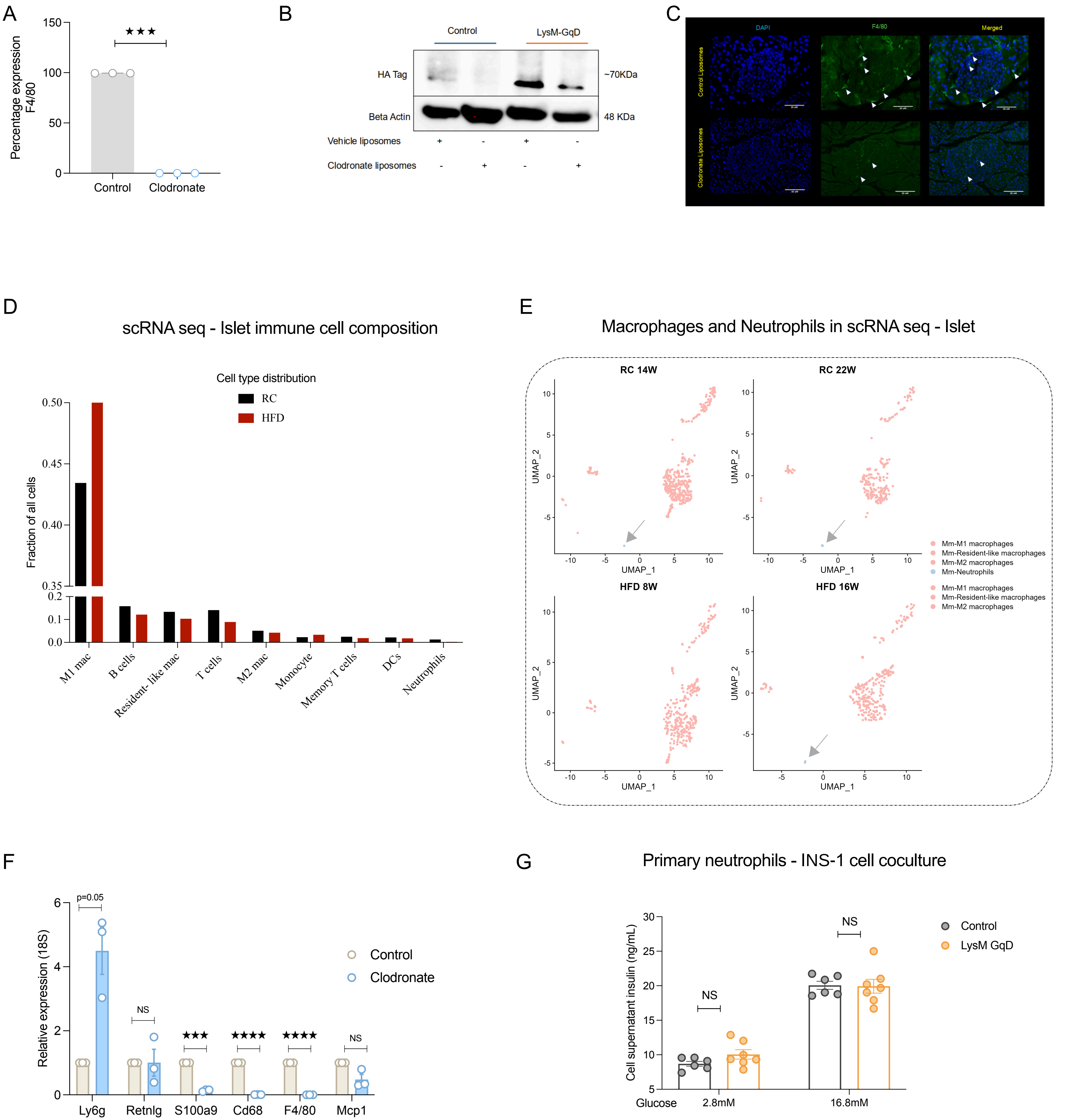

A
Percentage expression F4/80
150
100
50
0
★★★
Control
Clodronate
B
Control
LysM-GqD
HA Tag
~70KDa
Beta Actin
48 KDa
Vehicle liposomes
Clodronate liposomes
C
DAPI
F4/80
Merged
Control Liposomes
Clodronate Liposomes
D
scRNA seq - Islet immune cell composition
Cell type distribution
RC
HFD
Fraction of all cells
M1 mac
B cells
Resident- like mac
T cells
M2 mac
Monocyte
Memory T cells
DCs
Neutrophils
E
Macrophages and Neutrophils in scRNA seq - Islet
RC 14W
RC 22W
HFD 8W
HFD 16W
UMAP_1
UMAP_2
Mm-M1 macrophages
Mm-Resident-like macrophages
Mm-M2 macrophages
Mm-Neutrophils
F
Relative expression (18S)
p=0.05
NS
★★★
★★★★
Control
Clodronate
Ly6g
Retnlg
S100a9
Cd68
F4/80
Mcp1
G
Primary neutrophils - INS-1 cell coculture
Cell supernatant insulin (ng/mL)
NS
Control
LysM GqD
Glucose
2.8mM
16.8mM

Supplemental Figure 4. Clodronate liposome treatment effectively depletes islet macrophages. (**A**) Quantification of F4/80 mRNA expression in islets after clodronate liposome treatment compared with control liposomes (n = 3), (**B**) Immunoblot for HA-tag confirming expression of LysM-GqD in macrophages; β-actin serves as loading control. (**C**) Representative immunofluorescence images of pancreatic islets stained with DAPI (blue) and F4/80 (green) in control versus clodronate liposomes–treated mice. White arrowheads indicate F4/80$^+$ macrophages(n=3) (DAPI, blue; F4/80, green), scale bar=20μm. (**D**) Islet-derived immune cell distribution across RC and HFD intervention. This is derived from Singh et al., 2026 (GSE329967). (**E**) UMAP representation of islet immune cells showing macrophage and neutrophil populations across 14- and 22-week regular chow (RC) and 8- and 16-week high-fat diet (HFD) interventions. The grey arrow indicates the neutrophil cluster. This is derived from Singh *et al.,* 2026 (GSE329967). (**F**) Relative expression of *Ly6g, Retnlg* (neutrophil markers), *S100a9* (myeloid marker), *Cd68* and *F4/80* (macrophage markers), and *Mcp1* (monocyte marker) in islets after 48 h treatment with vehicle or clodronate (n = 3). (**G**) Supernatant insulin concentration in INS-1 cells at 2.8 or 16.8 mM glucose cocultured with isolated neutrophil from control and LysM-GqD treated with DCZ (n = 6-7). Data are presented as mean ± s.e.m. two-way ANOVA Bonferroni multiple comparison test *p < 0.05, **p < 0.01, ***p < 0.001; NS = not significant.

Figure S5

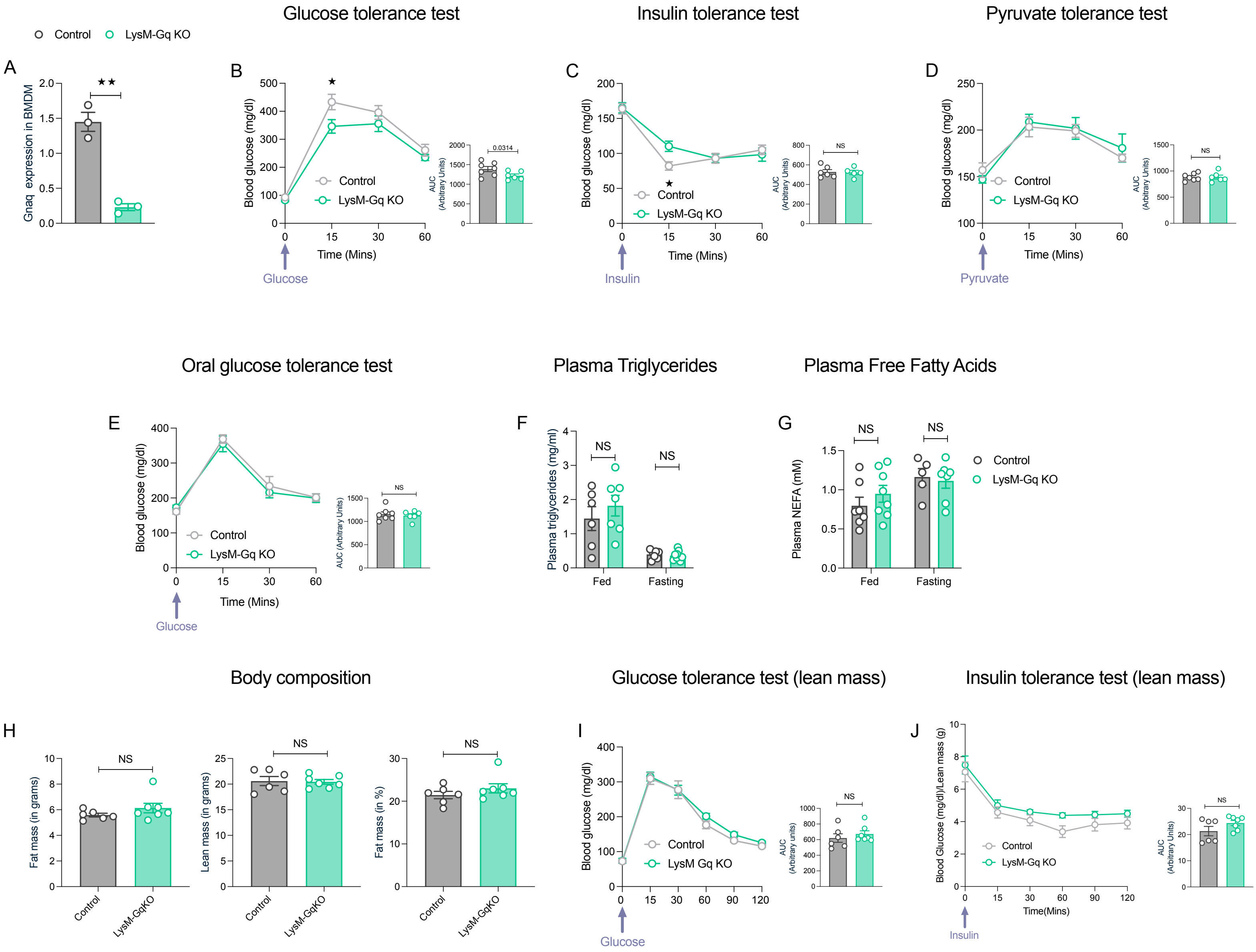
Glucose tolerance test
Insulin tolerance test
Pyruvate tolerance test
Control
LysM-Gq KO
A
Gnaq expression in BMDM
B
Blood glucose (mg/dl)
Time (Mins)
Glucose
AUC (Arbitrary Units)
0.0314
C
Insulin
NS
D
Pyruvate
Oral glucose tolerance test
Plasma Triglycerides
Plasma Free Fatty Acids
E
F
Plasma triglycerides (mg/ml)
Fed
Fasting
G
Plasma NEFA (mM)
Body composition
Glucose tolerance test (lean mass)
Insulin tolerance test (lean mass)
H
Fat mass (in grams)
Lean mass (in grams)
Fat mass (in %)
LysM-GqKO
I
LysM Gq KO
J
Blood Glucose (mg/dl)/Lean mass (g)
Time(Mins)

Supplemental Figure 5. Glucose and lipid metabolic profiling in LysM-Gq KO mice. (**A**) *Gnaq* expression levels in BMDMs from control and LysM-Gq KO mice (n=3). (**B–E**) Glucose tolerance test (GTT) (n=6-7) (**B**), insulin tolerance test (ITT) (n=6-7) (**C**), pyruvate tolerance test (PTT) (n=6-7) (**D**), and oral glucose tolerance test (O-GTT) (n=6-7) (**E**) in control and LysM-Gq KO mice. Knockout mice show modest differences in glucose handling, with largely comparable overall glucose excursions (AUC). (**F–G**) Plasma profiling in fed and fasted states. Plasma triglycerides (n=7-9) (**F**) and free fatty acids (n=7-9) (**G**) in control and LysM-Gq KO mice. (**H**) Body composition analysis of control and LysM-Gq KO mice on RC diet. Fat mass (left), Lean mass (center) and Fat mass percentage (right). (**I**) Intraperitoneal GTT in control and LysM-Gq KO mice on RC diet. Glucose was administered based on lean body mass. (**J**) ITT in control and LysM-Gq KO mice on RC diet. Insulin (0.75 U/kg, i.p.) was administered with dosing normalized to lean body mass. Data are presented as mean ± s.e.m. two-way ANOVA Bonferroni multiple comparison test $^{*}p < 0.05$, $^{**}p < 0.01$, $^{***}p < 0.001$; NS = not significant.

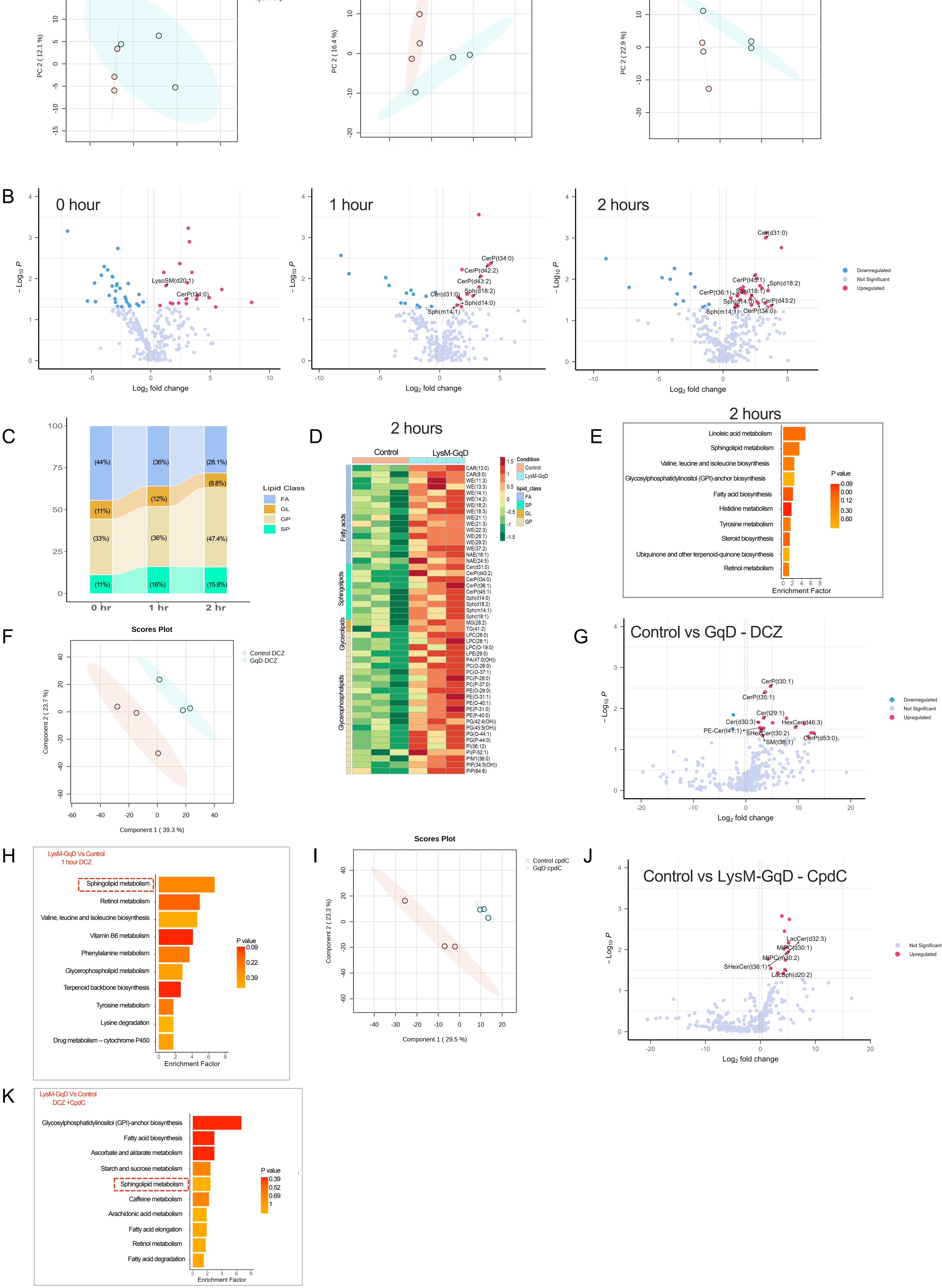
PC 2 (12.1 %)
PC 2 (16.4 %)
PC 2 (22.9 %)
B
0 hour
1 hour
2 hours
Log2 fold change
− Log10 P
LysoSM(d20:1)
CerP(t34:0)
CerP(d42:2)
CerP(d43:2)
Sph(d18:2)
Cer(d31:0)
Sph(d14:0)
Sph(m14:1)
Cer(d31:0)
CerP(t45:1)
Sph(d18:2)
CerP(t36:1)
Sph(t18:1)
Sph(d14:0)
CerP(d43:2)
Sph(m14:1)
CerP(t34:0)
Downregulated
Not Significant
Upregulated
C
Lipid Class
FA
GL
GP
SP
(44%)
(36%)
(28.1%)
(12%)
(8.8%)
(11%)
(33%)
(36%)
(47.4%)
(11%)
(16%)
(15.8%)
0 hr
1 hr
2 hr
D
2 hours
Control
LysM-GqD
Condition
Control
LysM-GqD
lipid_class
FA
SP
GL
GP
Fatty acids
Sphingolipids
Glycerolipids
Glycerophospholipids
CAR(13:0)
CAR(8:0)
WE(11:3)
WE(13:3)
WE(14:1)
WE(14:2)
WE(18:2)
WE(18:3)
WE(21:1)
WE(21:3)
WE(22:3)
WE(26:1)
WE(29:2)
WE(37:2)
NAE(16:1)
NAE(24:5)
Cer(d31:0)
CerP(d43:2)
CerP(t34:0)
CerP(t36:1)
CerP(t45:1)
Sph(d14:0)
Sph(d18:2)
Sph(m14:1)
Sph(t18:1)
MG(28:2)
TG(41:2)
LPC(26:0)
LPC(28:1)
LPC(O-19:0)
LPE(29:0)
PA(47:0(OH))
PC(O-26:0)
PC(O-37:1)
PC(P-28:0)
PC(P-37:0)
PE(O-29:0)
PE(O-31:1)
PE(O-40:1)
PE(P-31:0)
PE(P-40:0)
PG(42:4(OH))
PG(45:5(OH))
PG(O-44:1)
PG(P-44:0)
PI(56:12)
PI(P-52:1)
PIM1(36:0)
PIP(34:5(OH))
PIP(64:6)
E
2 hours
Linoleic acid metabolism
Sphingolipid metabolism
Valine, leucine and isoleucine biosynthesis
Glycosylphosphatidylinositol (GPI)-anchor biosynthesis
Fatty acid biosynthesis
Histidine metabolism
Tyrosine metabolism
Steroid biosynthesis
Ubiquinone and other terpenoid-quinone biosynthesis
Retinol metabolism
P value
Enrichment Factor
F
Scores Plot
Control DCZ
GqD DCZ
Component 1 (39.3 %)
Component 2 (23.7 %)
G
Control vs GqD - DCZ
CerP(t30:1)
CerP(t35:1)
Cer(t29:1)
Cer(d30:3)
HexCer(d46:3)
PE-Cer(t41:1)
SHexCer(t30:2)
CerP(d53:0)
SM(t38:1)
H
LysM-GqD Vs Control
1 hour DCZ
Sphingolipid metabolism
Retinol metabolism
Valine, leucine and isoleucine biosynthesis
Vitamin B6 metabolism
Phenylalanine metabolism
Glycerophospholipid metabolism
Terpenoid backbone biosynthesis
Tyrosine metabolism
Lysine degradation
Drug metabolism – cytochrome P450
I
Scores Plot
Control cpdC
GqD cpdC
Component 1 (29.5 %)
Component 2 (23.3 %)
J
Control vs LysM-GqD - CpdC
LacCer(d32:3)
MIPC(d30:1)
MIPC(m30:2)
SHexCer(t36:1)
LacSph(d20:2)
K
LysM-GqD Vs Control
DCZ +CpdC
Glycosylphosphatidylinositol (GPI)-anchor biosynthesis
Fatty acid biosynthesis
Ascorbate and aldarate metabolism
Starch and sucrose metabolism
Sphingolipid metabolism
Caffeine metabolism
Arachidonic acid metabolism
Fatty acid elongation
Retinol metabolism
Fatty acid degradation

Supplemental Figure 6. Lipidomic profiling reveals dynamic lipid remodeling in LysM-GqD macrophages. (**A–B**) Principal component analysis (PCA) (**A**) and volcano plots (**B**) of cell lysate lipids at 0, 1, and 2 hours after DCZ stimulation, showing distinct clustering and significant lipid changes at 2 hours. (**C**) Distribution of lipid classes contributing to differential regulation. (**D**) Heatmap depicting differentially regulated lipid species at 2 hours in control versus LysM-GqD macrophages. (**E**) Pathway enrichment analysis identifying lipid metabolic pathways significantly modulated upon Gq activation for 2 hours. (**F**) PLS-DA and (**G**) volcano plots of lipid changes in cell supernatants comparing control vs LysM-GqD cells following DCZ stimulation. (**H**) Pathway enrichment analysis showing upregulated pathways in the supernatant of LysM-GqD BMDMs compared to control BMDMs following 1 hour DCZ treatment (n = 3). (**I**) PLS-DA and (**J**) Volcano plot comparing lipid changes between control and LysM-GqD macrophages after CpdC treatment. (**K**) Pathway enrichment analysis showing enriched pathways in BMDMs treated with CpdC during DCZ stimulation from control or LysM-GqD (n = 3). All experiments were performed with three independent biological replicates and two technical replicates per sample.

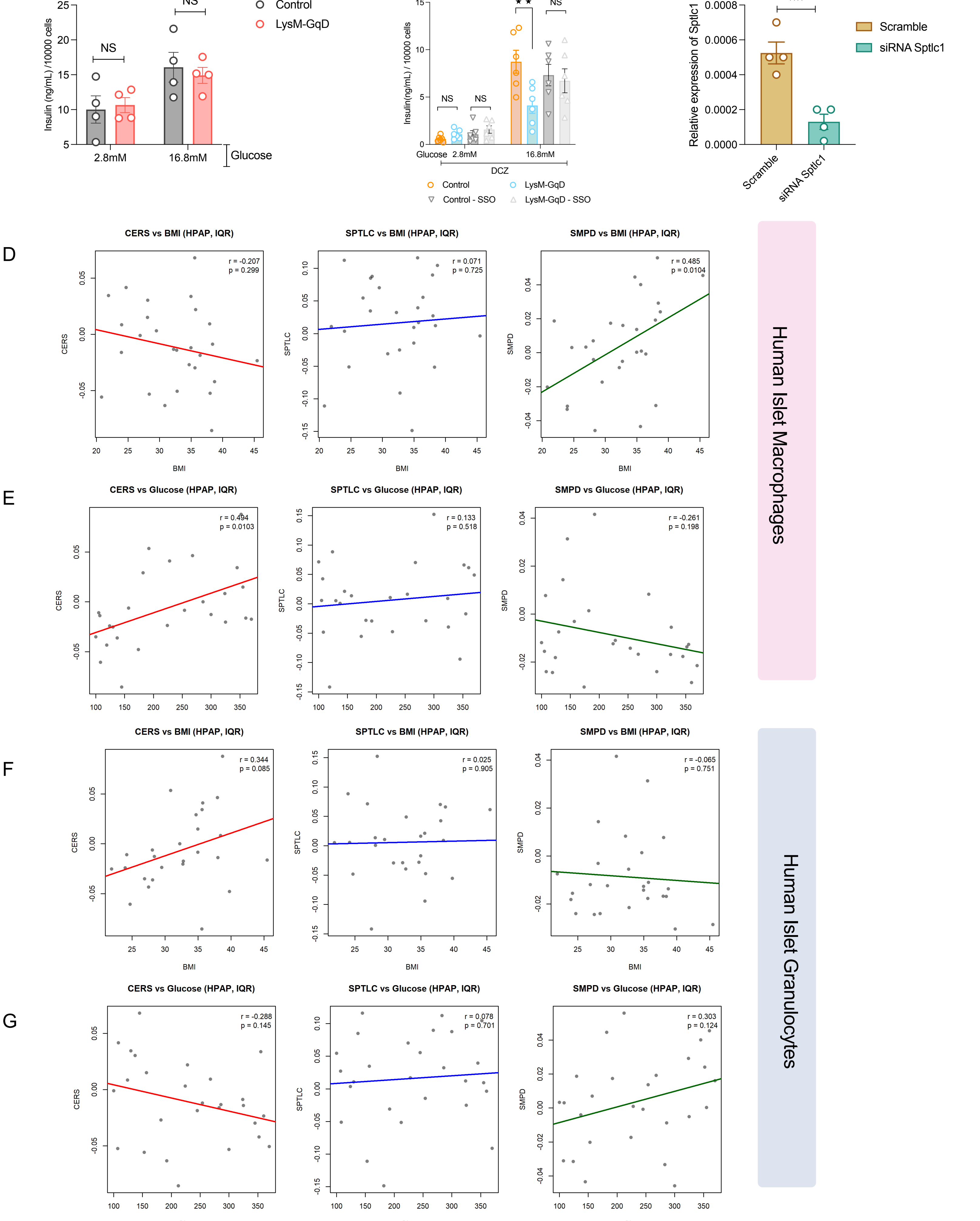

Control
LysM-GqD
Insulin (ng/mL) /10000 cells
NS
NS
2.8mM
16.8mM
Glucose
Insulin(ng/mL) / 10000 cells
NS
NS
NS
Glucose 2.8mM
16.8mM
DCZ
Control
LysM-GqD
Control - SSO
LysM-GqD - SSO
Relative expression of Sptlc1
Scramble
siRNA Sptlc1
D
CERS vs BMI (HPAP, IQR)
r = -0.207
p = 0.299
SPTLC vs BMI (HPAP, IQR)
r = 0.071
p = 0.725
SMPD vs BMI (HPAP, IQR)
r = 0.485
p = 0.0104
CERS
SPTLC
SMPD
BMI
E
CERS vs Glucose (HPAP, IQR)
r = 0.494
p = 0.0103
SPTLC vs Glucose (HPAP, IQR)
r = 0.133
p = 0.518
SMPD vs Glucose (HPAP, IQR)
r = -0.261
p = 0.198
Human Islet Macrophages
F
CERS vs BMI (HPAP, IQR)
r = 0.344
p = 0.085
SPTLC vs BMI (HPAP, IQR)
r = 0.025
p = 0.905
SMPD vs BMI (HPAP, IQR)
r = -0.065
p = 0.751
G
CERS vs Glucose (HPAP, IQR)
r = -0.288
p = 0.145
SPTLC vs Glucose (HPAP, IQR)
r = 0.078
p = 0.701
SMPD vs Glucose (HPAP, IQR)
r = 0.303
p = 0.124
Glucose
Human Islet Granulocytes

Supplemental Figure 7. Circulating and macrophage-derived sphingolipids suppress β-cell function and are associated with metabolic dysfunction in human islet immune cells. (**A**) Insulin secretion in INS-1 cells at 2.8 or 16.8 mM glucose after transfer of 3% blood plasma from control or LysM-GqD animals upon 45 mins stimulation with DCZ (30 nM) (n=4). (**B**) Insulin secretion in INS-1 cells after transfer of supernatants from control or LysM-GqD BMDMs stimulated with DCZ (30 nM) at 2.8 or 16.8 mM glucose, with or without the CD36 inhibitor sulfosuccinimidyl-oleate (SSO; 0.1 mmol/l; n=6). (**C**) Relative expression of *Sptlc1* in scramble and Sptlc1 siRNA treated BMDM for 48 hours (**D-E**) *CerS* (left), *Sptlc* (middle) and *Smpd* (right) gene modules expression vs glucose (**D**), BMI (**E**) in Human Pancreas Analysis Program (HPAP) scRNA-seq human pancreatic islet macrophages. (**F-G**) *CerS* (left), *Sptlc* (middle) and *Smpd* (right) gene modules expression vs glucose (**F**) BMI (**G**) in HPAP scRNA-seq human pancreatic islet granulocytes. Data are presented as mean ± s.e.m. two-way ANOVA Bonferroni multiple comparison test *p < 0.05, **p < 0.01, ***p < 0.001; NS = not significant.

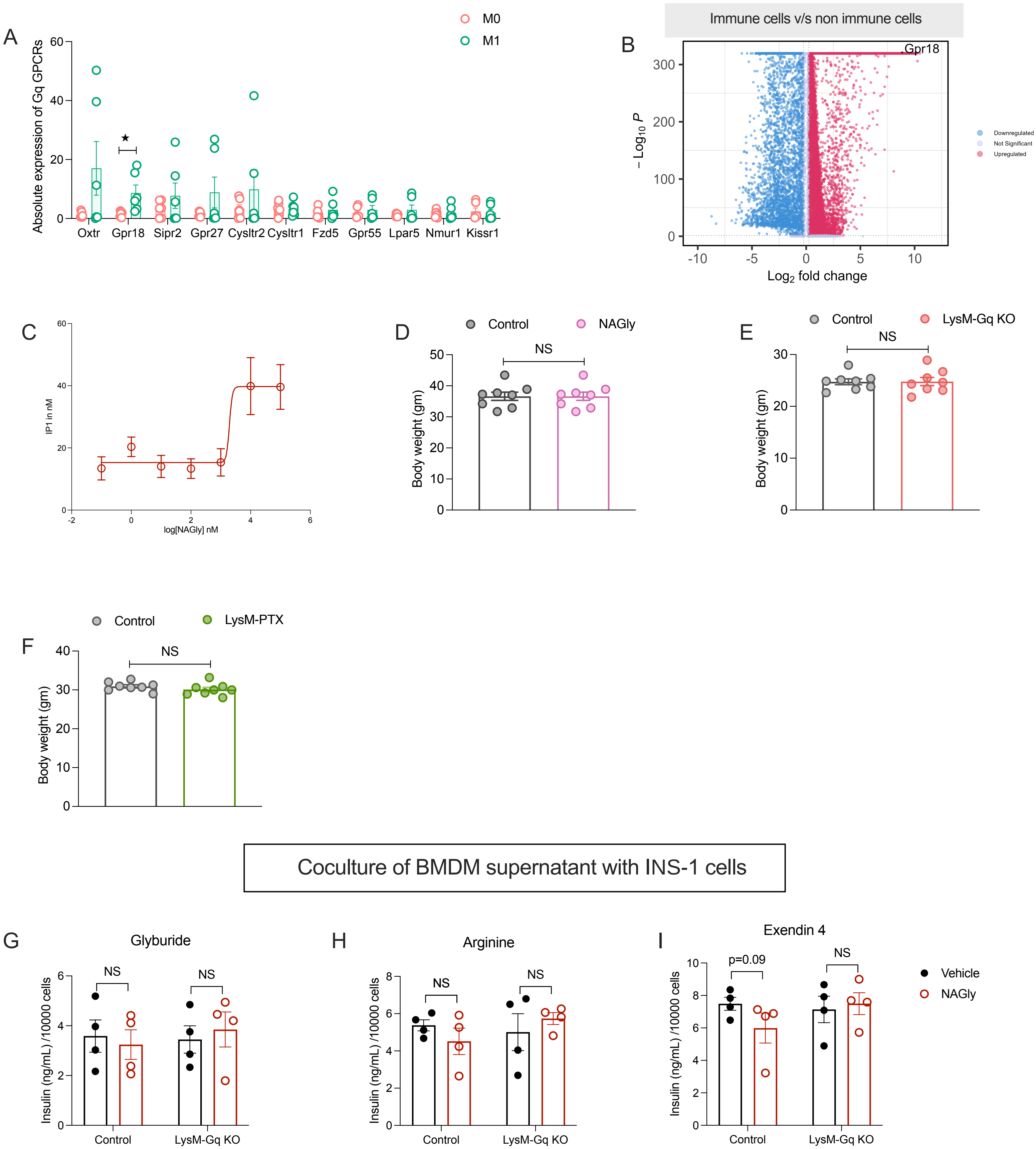
A
M0
M1
Absolute expression of Gq GPCRs
60
40
20
0
★
Oxtr
Gpr18
Sipr2
Gpr27
Cysltr2
Cysltr1
Fzd5
Gpr55
Lpar5
Nmur1
Kissr1
B
Immune cells v/s non immune cells
Gpr18
300
200
100
0
-10
-5
0
5
10
Log2 fold change
– Log10 P
Downregulated
Not Significant
Upregulated
C
IP1 in nM
60
40
20
0
-2
0
2
4
6
log[NAGly] nM
D
Control
NAGly
NS
Body weight (gm)
50
40
30
20
10
0
E
Control
LysM-Gq KO
NS
Body weight (gm)
30
20
10
0
F
Control
LysM-PTX
NS
Body weight (gm)
40
30
20
10
0
Coculture of BMDM supernatant with INS-1 cells
G
Glyburide
NS
NS
Insulin (ng/mL) /10000 cells
6
4
2
0
Control
LysM-Gq KO
H
Arginine
NS
NS
Insulin (ng/mL) /10000 cells
8
6
4
2
0
Control
LysM-Gq KO
I
Exendin 4
p=0.09
NS
Insulin (ng/mL) /10000 cells
10
8
6
4
2
0
Control
LysM-Gq KO
Vehicle
NAGly

Supplemental Figure 8. Gpr18 expression and functional assessment in islet macrophages and LysM-Gq KO models. (**A**) Relative GPCRs expression profiling in murine islet macrophages reveals *Gpr18* enrichment (n=6). (**B**) Volcano plot showing upregulated expression of *Gpr18* in scRNA-seq pancreatic islet immune cells from RC, HFD fed and db/db background mice. (**C**) IP-One assay demonstrating NAGly (GPR18 agonist)–induced, dose-dependent IP1 accumulation in DCZ-stimulated GqD macrophages (n=6). (**D-F**) Body weight analysis showing no significant differences between control and NAGly-treated mice (n=8) (**D**). LysM-Gq KO and control mice (n=8) (**E**), and LysM-PTX and control mice (n=8) (**F**). (**G–I**) Coculture of BMDM and INS-1 cell for insulin secretion assays in control and LysM-Gq KO BMDMs following stimulation with glyburide (n=4) (**G**), arginine (n=4) (**H**), or exendin-4 (n=4) (**I**). Data are presented as mean ± s.e.m. two-way ANOVA Bonferroni multiple comparison test $^{*}p < 0.05$, $^{**}p < 0.01$, $^{***}p < 0.001$; NS = not significant.

Figure S9

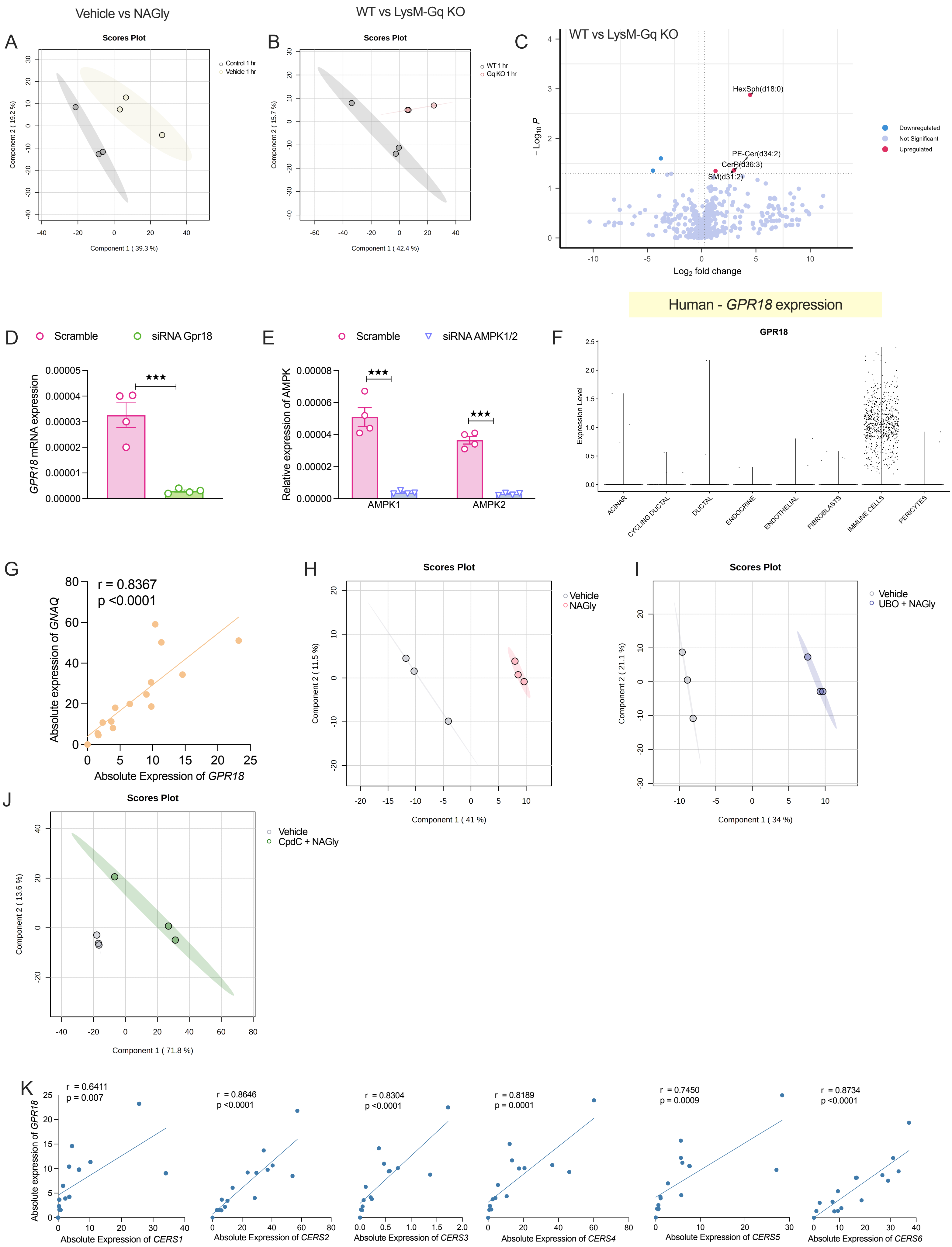

Vehicle vs NAGly
WT vs LysM-Gq KO
A
Scores Plot
Control 1 hr
Vehicle 1 hr
Component 1 (39.3 %)
Component 2 (19.2 %)
B
Scores Plot
WT 1 hr
Gq KO 1 hr
Component 1 (42.4 %)
Component 2 (15.7 %)
C
WT vs LysM-Gq KO
HexSph(d18:0)
PE-Cer(d34:2)
CerP(d36:3)
SM(d31:2)
Downregulated
Not Significant
Upregulated
Log2 fold change
Human - GPR18 expression
D
Scramble
siRNA Gpr18
GPR18 mRNA expression
E
Scramble
siRNA AMPK1/2
Relative expression of AMPK
AMPK1
AMPK2
F
GPR18
Expression Level
ACINAR
CYCLING DUCTAL
DUCTAL
ENDOCRINE
ENDOTHELIAL
FIBROBLASTS
IMMUNE CELLS
PERICYTES
G
r = 0.8367
p <0.0001
Absolute expression of GNAQ
Absolute Expression of GPR18
H
Scores Plot
Vehicle
NAGly
Component 1 (41 %)
Component 2 (11.5 %)
I
Scores Plot
Vehicle
UBO + NAGly
Component 1 (34 %)
Component 2 (21.1 %)
J
Scores Plot
Vehicle
CpdC + NAGly
Component 1 (71.8 %)
Component 2 (13.6 %)
K
r = 0.6411
p = 0.007
r = 0.8646
p <0.0001
r = 0.8304
p <0.0001
r = 0.8189
p = 0.0001
r = 0.7450
p = 0.0009
r = 0.8734
p <0.0001
Absolute expression of GPR18
Absolute Expression of CERS1
Absolute Expression of CERS2
Absolute Expression of CERS3
Absolute Expression of CERS4
Absolute Expression of CERS5
Absolute Expression of CERS6

Supplemental Figure 9. Lipidomic profiling of Control and Gq KO islets following vehicle and NAGly treatments. PLS-DA of (**A**) vehicle-treated vs NAGly-treated for 1 hour in WT mice (**B**) WT vs LysM-Gq KO for 1 hour. (**C)** Volcano plots of differential lipid species ($\log_2$ fold change vs –$\log_{10}$ p-value) after NAGly treatment: WT vs LysM-Gq KO BMDM (**D**) Relative expression of Gpr18 in scramble and Gpr18 siRNA treated BMDM for 48 hours (**E**) Relative expression of AMPK1/2 in scramble and AMPK1/2 siRNA treated BMDM for 48 hours (**F**) Violin plot of scRNA-seq data showing GPR18 expression across pancreatic islet cell types. (**G**) Correlation between Absolute expression of GNAQ and GPR18 in human PBMC. (**H-J**) PLS-DA of (**H**) vehicle vs 1hour NAGly; (**I**) vehicle vs UBO+NAGly; (**J**) vehicle vs CpdC+NAGly in PBMC. (**K**) Correlations between GPR18 and CERS1–6 expression in human PBMCs.
