# Supplementary material for: Acute activation of Gq signaling in pancreatic islet macrophages inhibits insulin secretion through AMPK/sphingolipid axis
Source: J Clin Invest. 2026 Sep 15;136(22):e202344. doi: 10.1172/JCI202344 (PMC13767642; doi:10.1172/JCI202344)

Figure 1.G

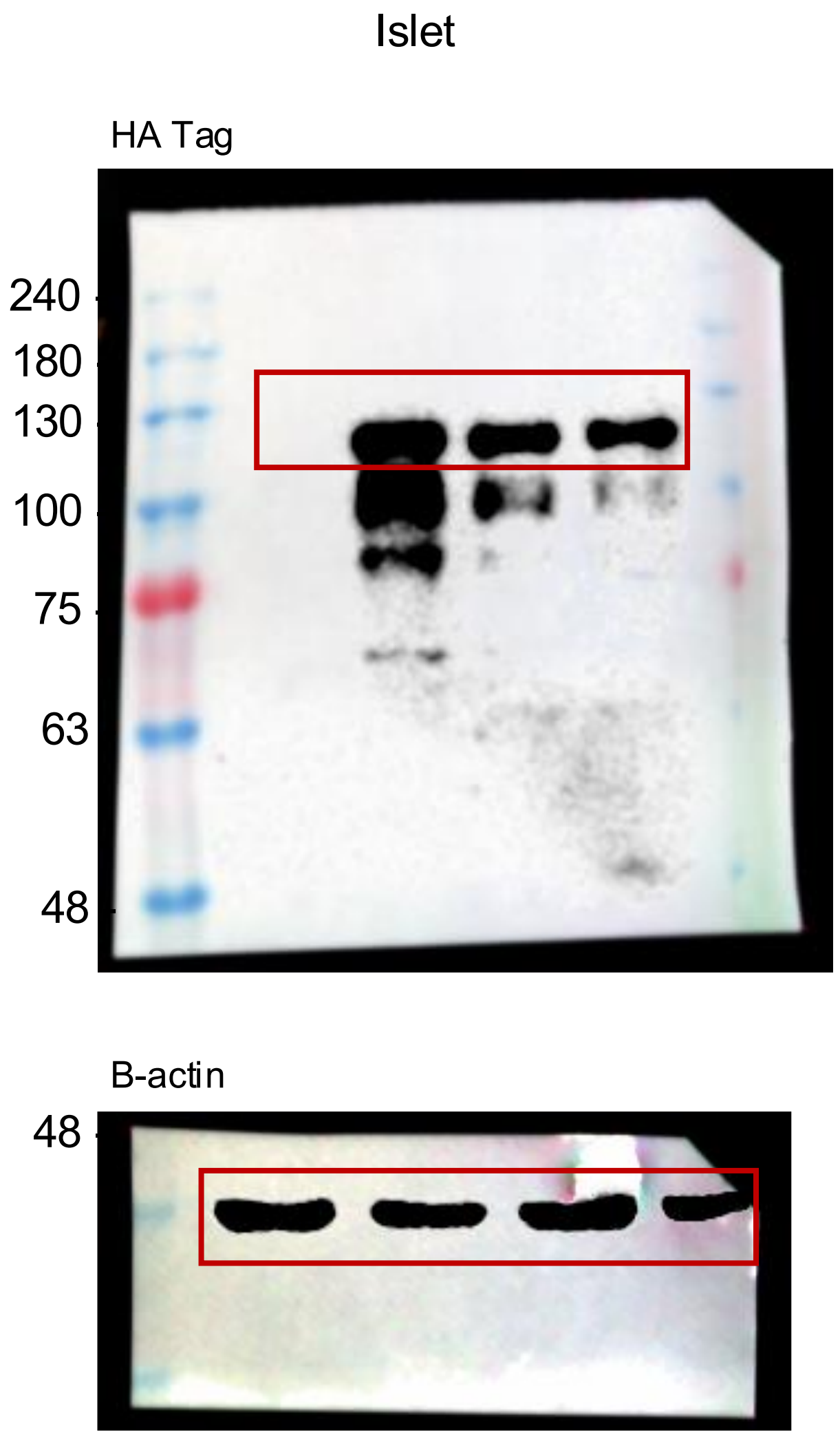

Figure 6.C

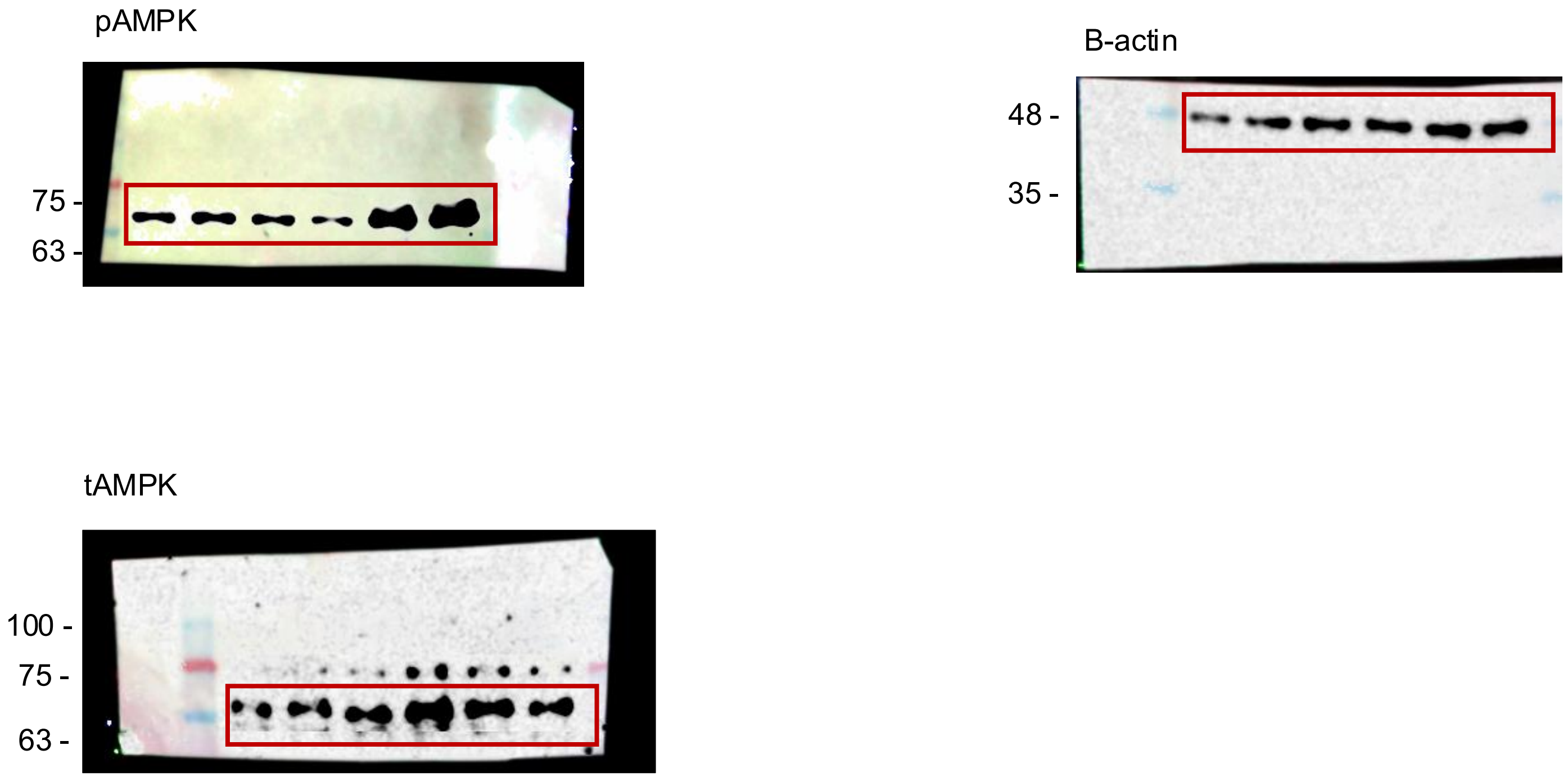

Figure 7.A

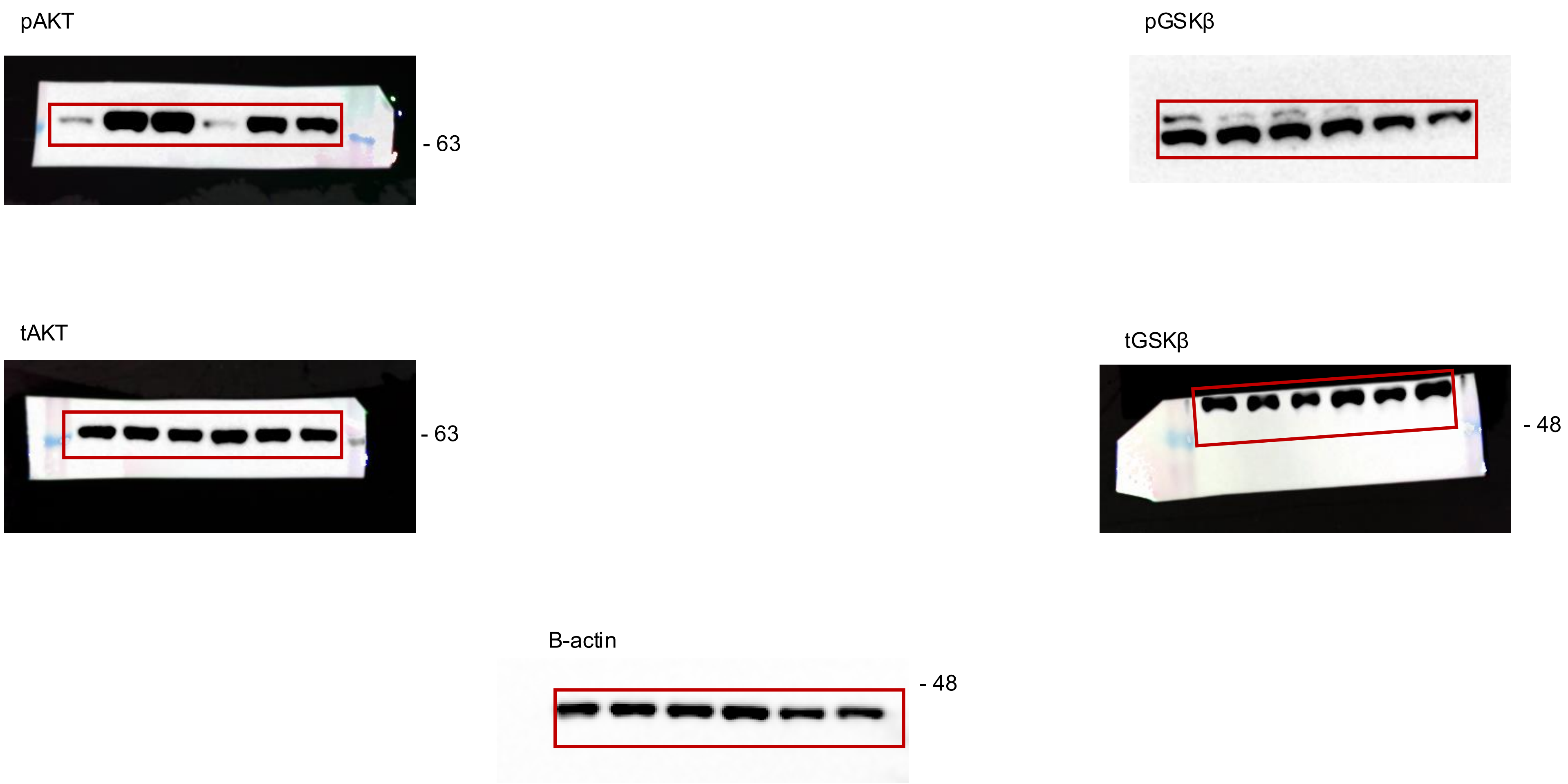

Figure 7.E

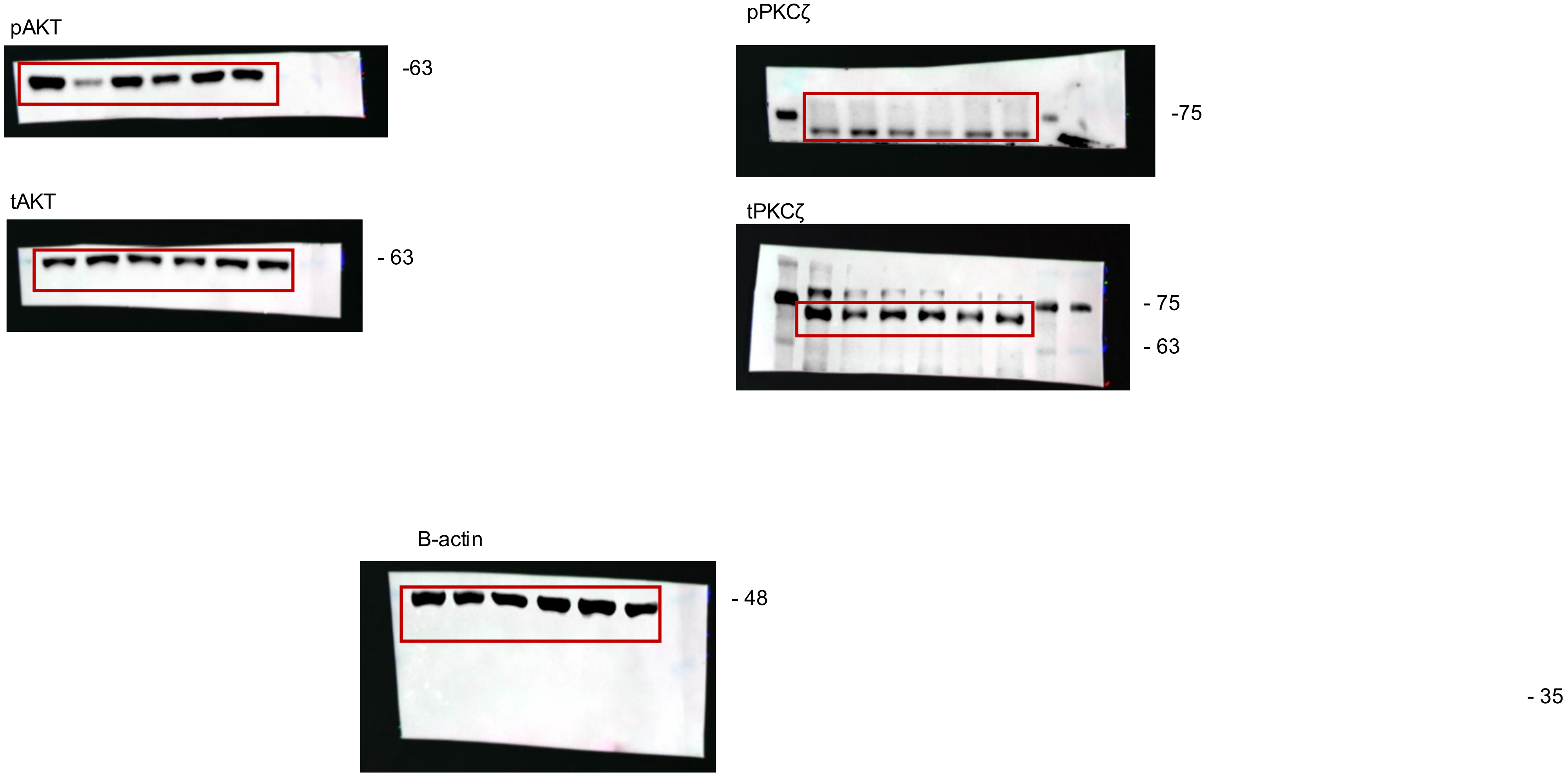

Figure 10.F

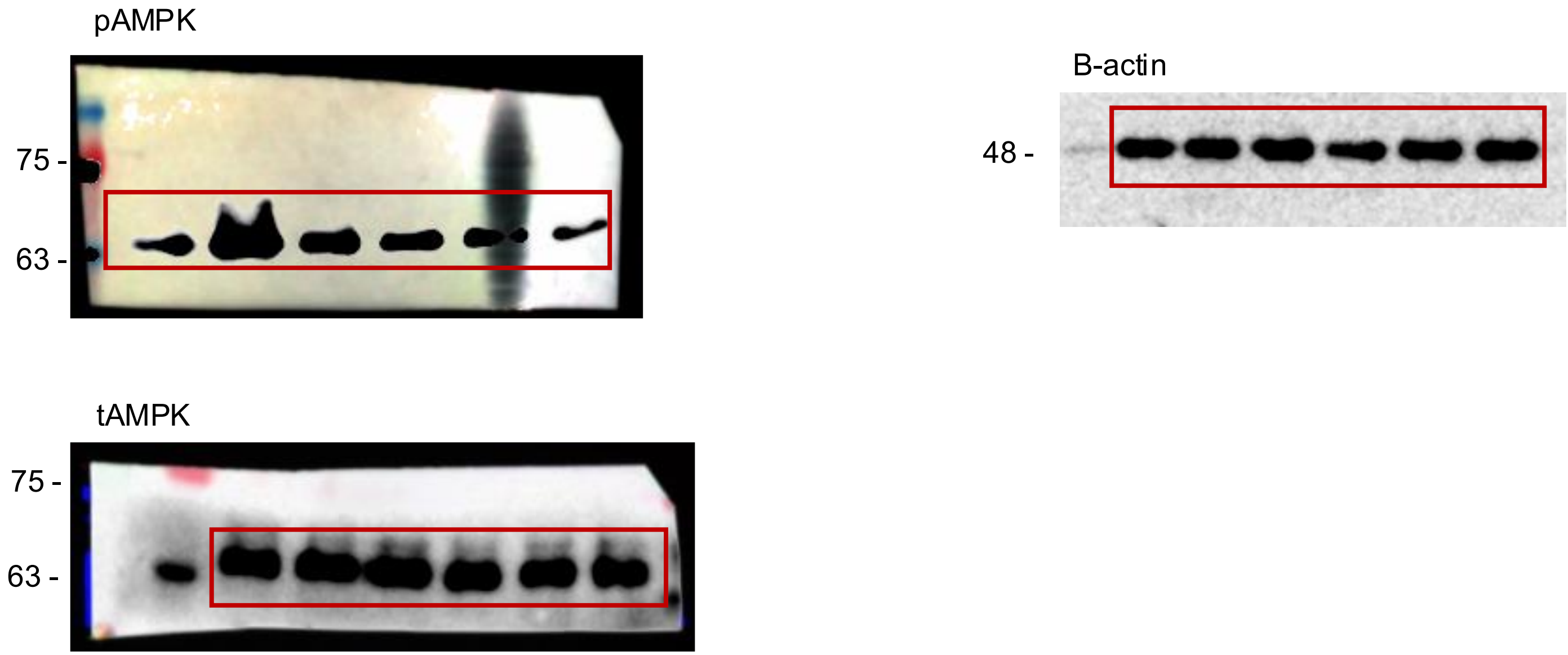

Figure 10.H

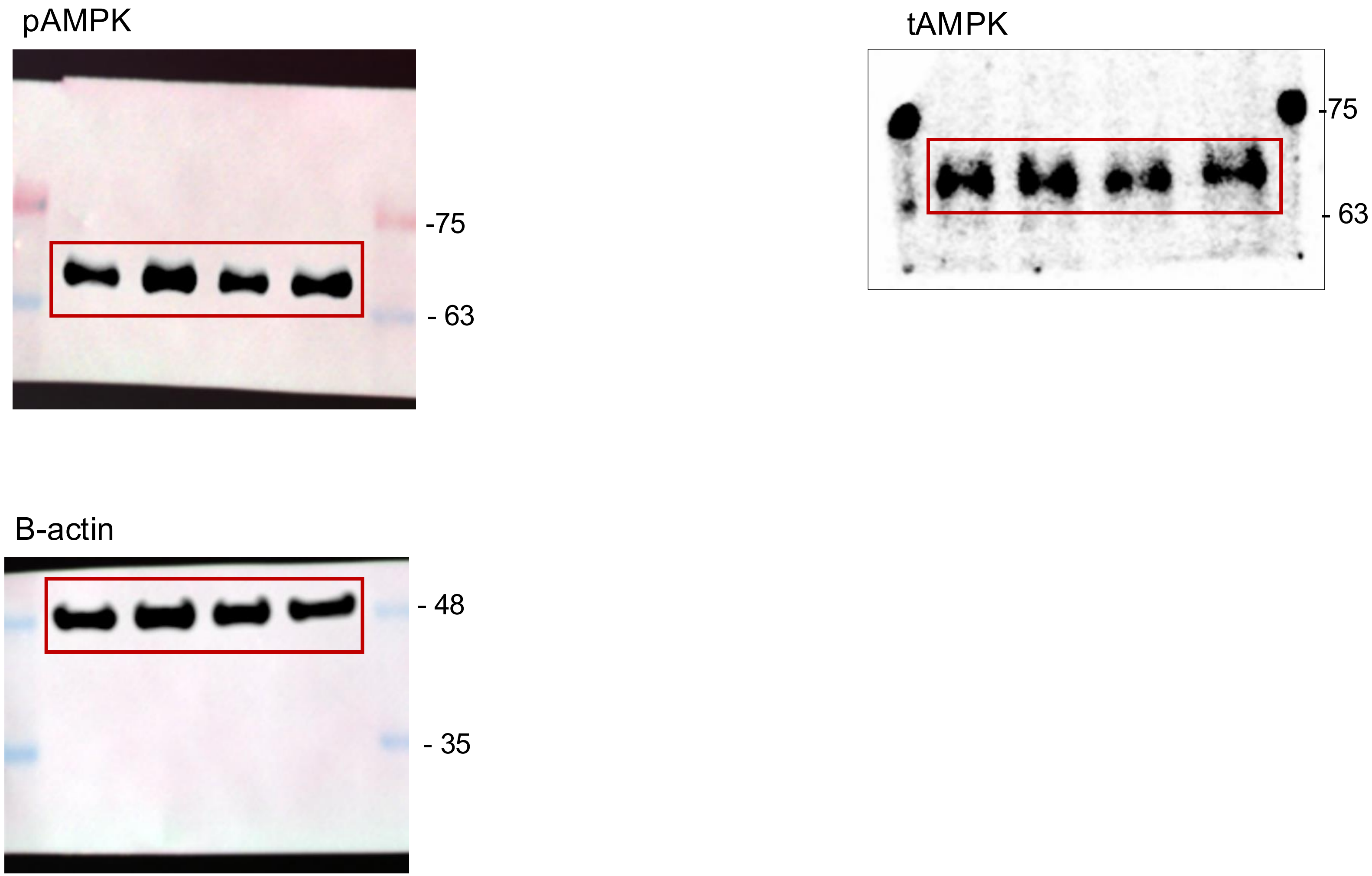

Figure 10.K

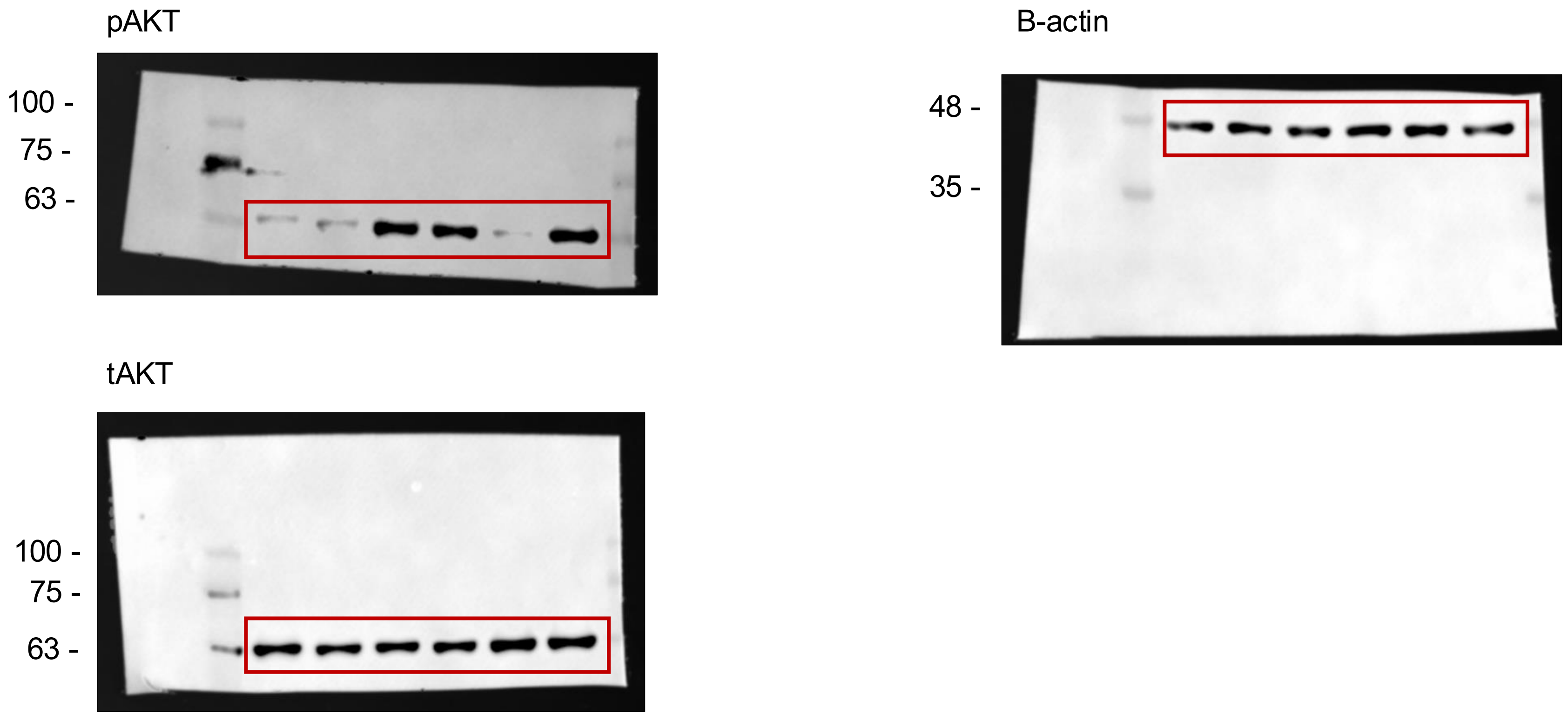

Figure 11.F

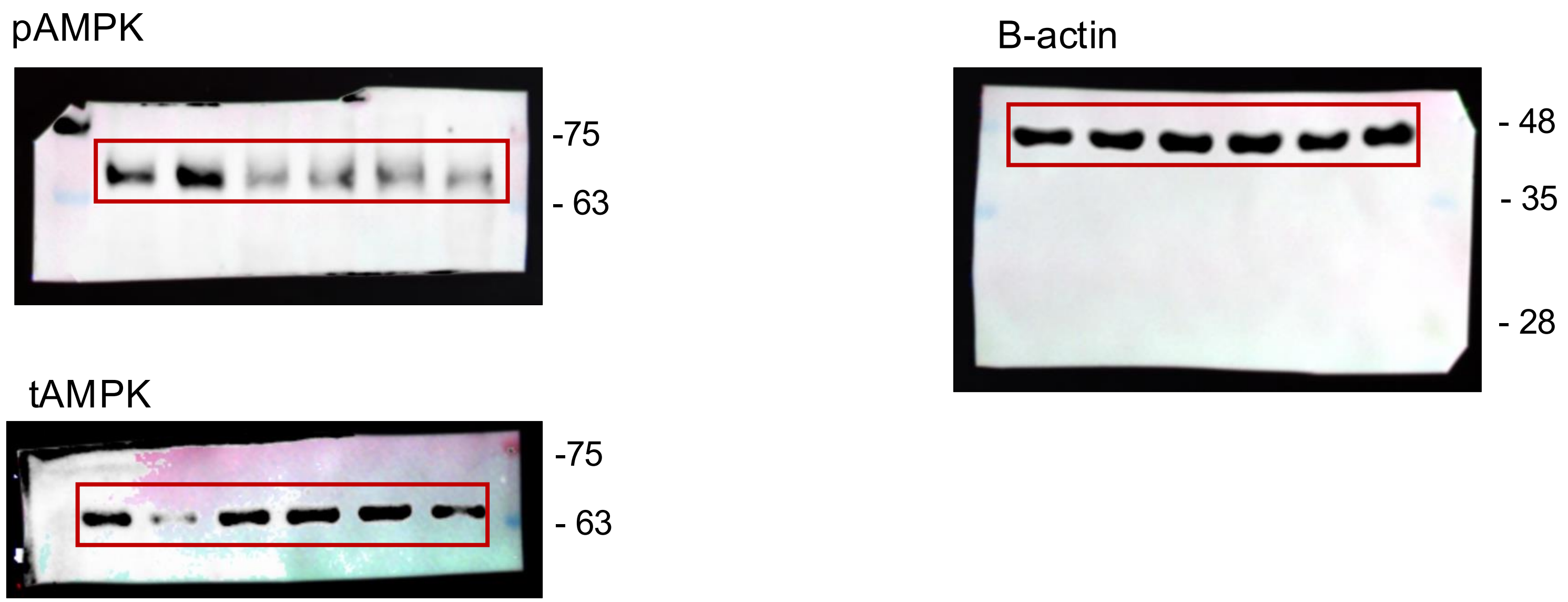

Figure S 1.M

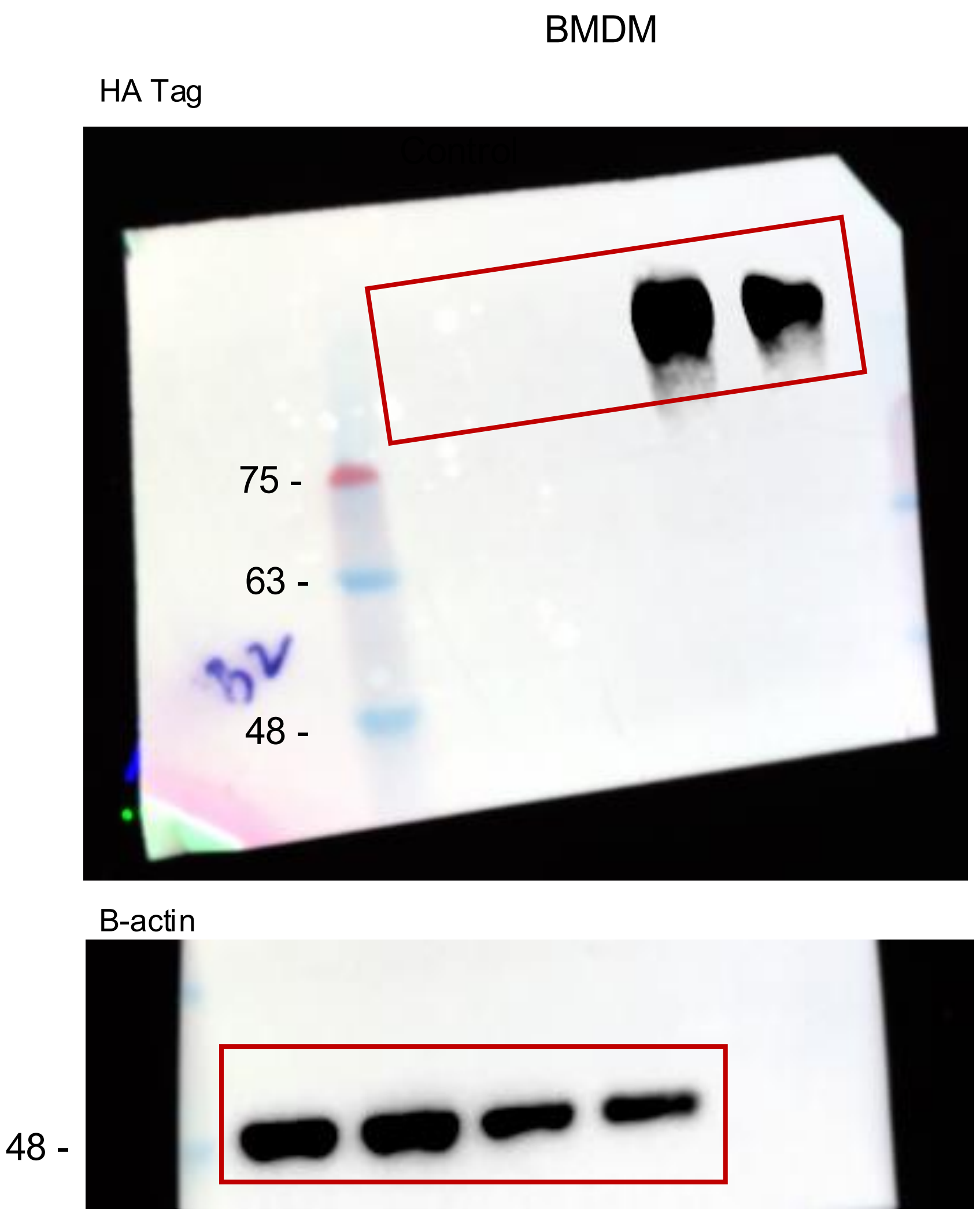

Figure S 5.B

HA Tag

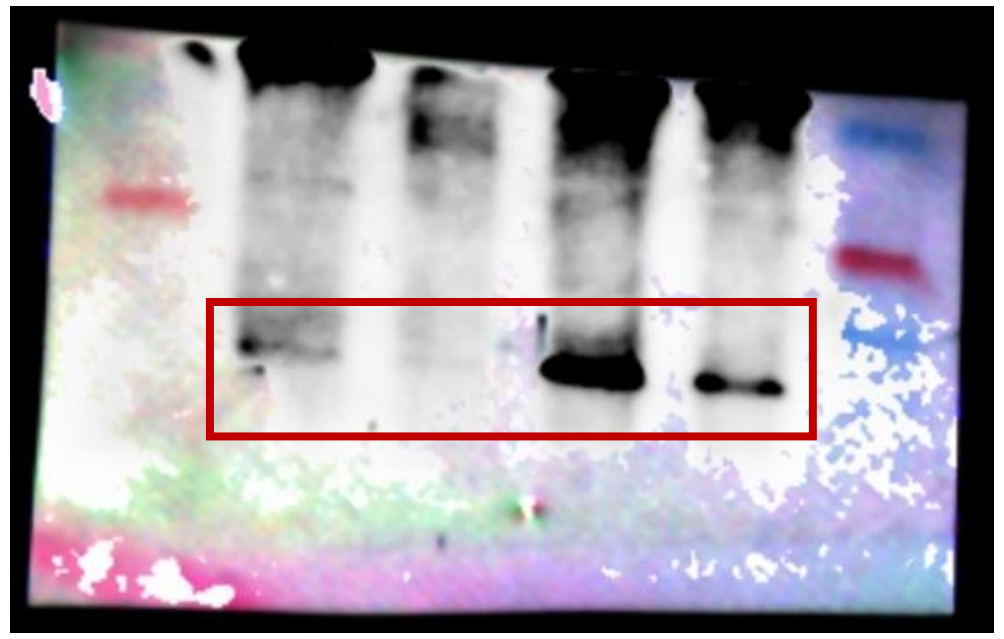

Beta Actin

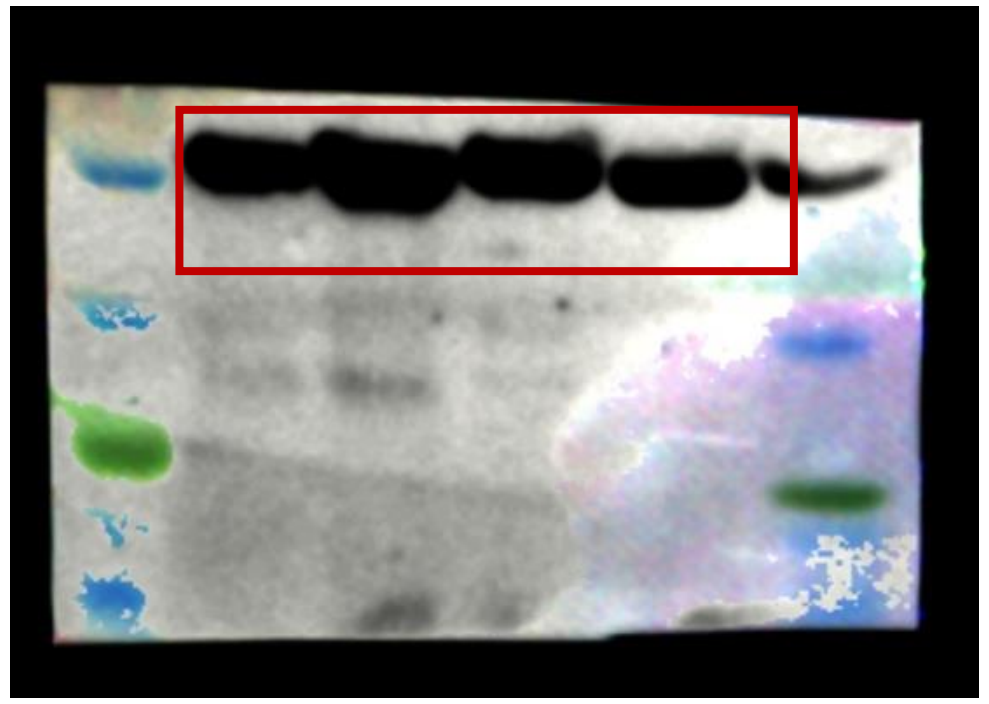

Supplement: Supplementary file 2 [file jci-136-202344-s127.pdf]
